# Checklist of the suborder Terebrantia (Thysanoptera): generic diversity and species composition in Xishuangbanna, Yunnan Province, China

**DOI:** 10.3897/BDJ.9.e72670

**Published:** 2021-11-24

**Authors:** Ntirenganya Elie, Li Yajin, Xie Yanlan, Zhou Yanli, Zhang Hongrui

**Affiliations:** 1 Plant Protection College, Yunnan Agricultural University, Kunming, 650201, China Plant Protection College, Yunnan Agricultural University Kunming, 650201 China; 2 Rwandan Association of Ecologists (ARECO Rwanda), Kigali, Rwanda Rwandan Association of Ecologists (ARECO Rwanda) Kigali Rwanda; 3 Agronomy and Biotechnology College, Yunnan Agricultural University, Kunming, 650201, China Agronomy and Biotechnology College, Yunnan Agricultural University Kunming, 650201 China; 4 Biotechnology and Engineering College, West Yunnan University, Lincang, 677000, China Biotechnology and Engineering College, West Yunnan University Lincang, 677000 China; 5 The Germplasm Bank of Wild Species, Kunming Institute of Botany, Chinese Academy of Sciences, Kunming, 650201, China The Germplasm Bank of Wild Species, Kunming Institute of Botany, Chinese Academy of Sciences Kunming, 650201 China

**Keywords:** thrips, host range, distribution, biodiversity, conservation

## Abstract

**Background:**

Thysanoptera is amongst the most predominant orders of insects in different ecological zones with worldwide distribution. Due to their small size, there is a large gap in their distribution and host range data. To the best of our knowledge, there is no investigation on the thrips distribution and their host range in Xishuangbanna. Currently, a total of 566 species in 155 genera are listed in China, of which 313 species represent Terebrantia.

**New information:**

In this study, a list of 116 species representing 55 genera within the families Aeolothripidae and Thripidae is provided. Two of these, *Dichromomothripsnakahari* Moud, 1976 (subfamily Thripinae) and *Phibalothripsrugosus* Kudo, 1979 (subfamily Panchaetothripinae) are recorded for the first time in China. Thrips species with their host ranges, habits and habitats are provided. Our study aims to contribute to the global biodiversity distribution data-gap of Thysanoptera for conservation purposes, as well as pest species targetting Integrated Pest Management tactics.

## Introduction

Thysanoptera (commonly known as thrips) is a group of small insects with body length ranging from 0.5 to 5.0 mm (except for a few tropical species, which may reach 14 mm). They are characterised by piercing-sucking with the distinction of only one completed left mandible (Hunter and Ullman 1992). Thrips exhibit high potential of sexual or parthenogenetic reproduction with rapid growth (Ramakrishna and Margabandhu 1931, Ananthakrishnan 1969) and dwell in a great diversity of habitats. More than 50% are mycophagous with the remaining phytophagous and predators. Besides, a few species have been recorded to annoy or bite people and cause non-severe skin irritation (Borror 1998).

The world updated checklist of Thysanoptera is totalling about 6,337 extant species representing 786 genera (ThripsWiki 2020). In China, the basic taxonomic work on Thysanoptera comprises two checklists (Zhang and Tong 1993, Mirab-balou et al. 2011), of which about 100 species representing 40 genera were individually studied from Xishuangbanna. However, there is no checklist of Thysanoptera diversity and species composition have been conducted in Xishuangbanna which is the home of a unique evergreen Tropical Rainforest and Botanical Garden and the most biodiversity-rich place in Yunnan Province, harbouring much of the biodiversity of China (Liu et al. 2015). This study aims to investigate the diversity of suborder Terebrantia (Thysanoptera) and its hosts which are important for developing of sustainable biodiversity, conservation and Integrated Pest Management (IPM).

## Materials and methods

### Description of study areas

The study was conducted from May 2015 to August 2020 in three counties of Xishuangbanna Prefecture, Yunnan Province, China. This area is located on the tropical northern edge of the southern tip of the mountainous zone of about 80 hectares. The tropical rainforest is completely similar to the typical equatorial tropical rainforest in fauna and flora characteristics (Cao et al. 2006). The tropical climate with annual average temperature varies from 18 to 21°C, rainfall from 1,100 mm to 1,900 mm, elevation from 477 to 2,429 m a.s.l. and sunshine 1,700 to 2,300 hours and hosting over 301 plant families and 2110 genera (Zhu et al. 2015).

### Sample collection

**Figure 1.**
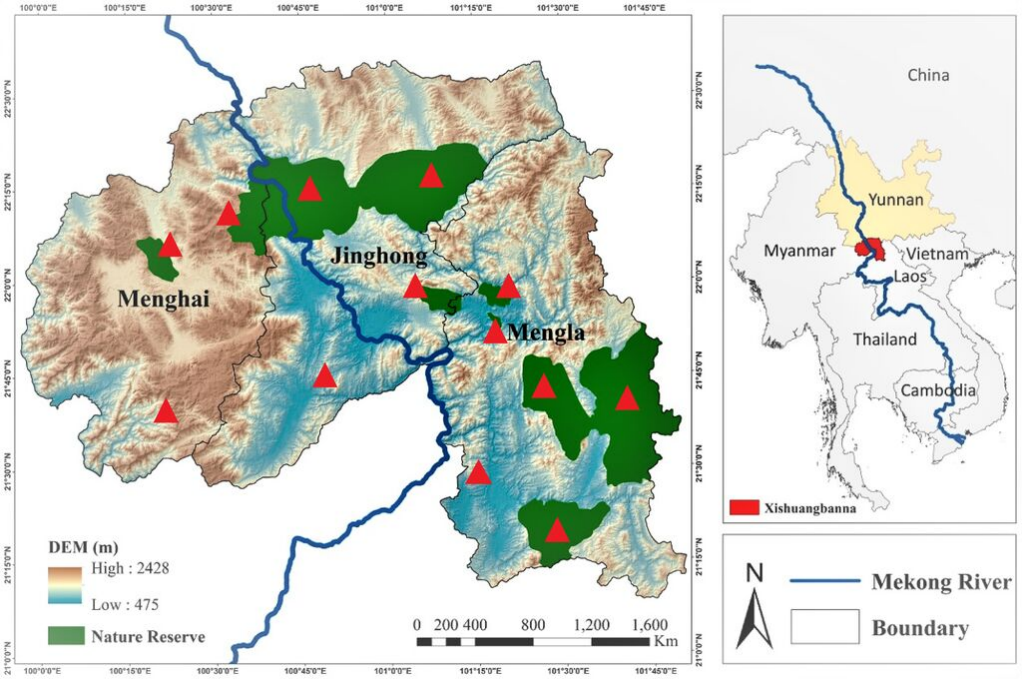
Geographical location of Xishuangbanna Prefecture Yunnan Province, China; the red pyramids show the geographic coverage of the sites of study.

Natural forests, protected areas, agricultural fields, botanical gardens, degraded and disturbed habitats were accessed and thrips samples were collected (Fig. 1), following the methodology as described by Zhang et al. (2006). For soft plants or shrubs foliage or inflorescence exposed to easy damage, the handshaking technique was applied. A fine hairbrush was used to pick thrips from the white tray and transferred to 75% ethanol to be carried to the Laboratory of Insect Taxonomy, College of Plant Protection, Yunnan Agricultural University and frozen for further studies. Besides, the references of a few individuals species, previously studied from Xishuangbanna, were collected.

### Permanent slides preparation

Adult thrips were mounted according to the standard slides preparation techniques (Zhang et al. 2006) and identified following the standard identification keys provided by Mound and Marullo (1996), Masumoto and Okajima (2006) and ThripsWiki (2020). Morphological characters were observed under a compound microscope Olympus BX 41. Photos of species were taken with a CCD Zeiss Microscope. Species distribution used thrips distribution information available (ThripsWiki 2020) and the Zoological Catalogue of Smithers et al. (1997).

Permanent slides were labelled with the site, host plant, date of sampling and collector’s name (left side); specimen ID, genus, species, sex and identified author’s name (right side). All slides were grouped by genus-species in boxes labelled with YAU5082020 (representing institutional code), Tt ((T) representing Thysanoptera and (t) Terebrantia in collection code), No. of slide ID Material is deposited in the Insect Taxonomy laboratory, Plant Protection College at Yunnan Agricultural University, Kunming, China. Some information on individual species was collected from previously-studied references.

## Checklists

### Checklist of the suborder Terebrantia (Thysanoptera) in Xishuangbanna (Yunnan Province, China)

#### Aeolothripidae

C91F71B3-F8CE-5DED-884F-B510E8051C2F

#### Mymarothrips garuda

Ramakrishna & Margabandhu, 1931

FBE2F876-D997-5740-A4F6-8EFB3CECA4F9

https://thrips.info/wiki/Mymarothrips_garuda

Mymarothrips garuda Ramakrishna & Margabandhu, 1931: 1031
Mymarothrips bolus Bhatti, 1967: 3. Synonymised by Mound and Marullo (1998)
Mymarothrips flavidonotus Tong & Zhang, 1995: 39.

##### Materials

- **Type status:** Other material. **Occurrence:** recordedBy: L.H; individualID: 2019-VII-17; individualCount: 2; sex: females; lifeStage: adults; occurrenceID: YAU5082020Tt1; **Taxon:** scientificNameAuthorship: *Mymarothripsgaruda* Ramakrishna & Margabandhu; **Location:** country: China; stateProvince: Yunnan; municipality: Xishuangbanna; locality: Mengla (Menglun), Jinghong; verbatimCoordinateSystem: 21°39'35.16''N, 101°25'43.56''E; decimalLatitude: 30.3427; decimalLongitude: 119.4338; **Identification:** identifiedBy: Liu Hui; dateIdentified: 2019; identificationReferences: (ThripsWiki 2020); **Event:** samplingProtocol: sweeping and shaking; eventDate: 17/07/2019; **Record Level:** collectionID: thrips; institutionCode: YAU5082020; collectionCode: terebrantia; basisOfRecord: preserved specimen

##### Ecological interactions

###### Feeds on

small arthropods.

##### Distribution

Described from India and recorded in southern China.

##### Diagnosis

**Figure 2.**
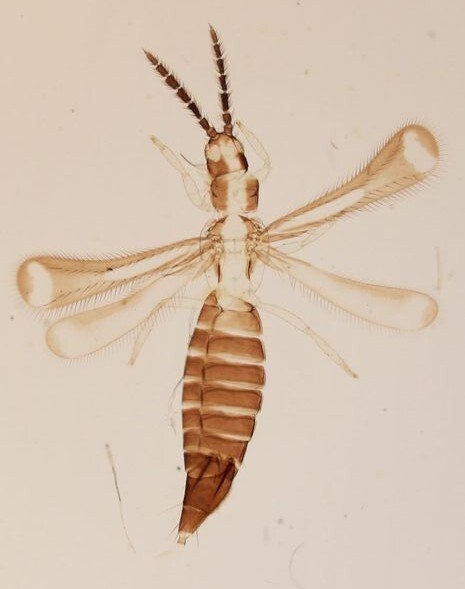
Female of *Mymarothripsgaruda* Ramakrishna & Margarbandhu, 1931.

Female macropterous, body yellowish-brown (Fig. 2), reddish pigments on abdominal segments 3-6, yellow median longitudinal stripe extending from ocellar region to the base of abdomen, cheeks brown; pronotum brown with reddish alongside the yellow median region; head longer than wider; prothorax broader than longer; fore wings with an abroad transversal colourless patch with grey before apex.

#### Thripidae

EC8BCA4E-007F-5141-9D70-80920D342261

#### Panchaetothripinae

0468B53D-E3D7-58DB-B12D-DCFFE6F91692

#### Anisopilothrips venustulus

(Priesner, 1923)

28B15181-7819-5EF6-8E53-6E5B2B4D2B37

https://thrips.info/wiki/Anisopilothrips_venustulus

Heliothrips venustulus Priesner, 1923: 89
Astrothrips angulatus Hood, 1925: 50. Synonymised by Wilson (1975).

##### Materials

- **Type status:** Other material. **Occurrence:** recordedBy: E.N. & X.Y.L; individualID: 2018-V-30|2019-V-18| 2019-V-16; individualCount: 6; sex: 1 male, 5 females; lifeStage: adults; occurrenceID: YAU5082020Tt2; **Taxon:** scientificNameAuthorship: *Anisopilothripsvenustulus* (Priesner); **Location:** country: China; stateProvince: Yunnan; municipality: Xishuangbanna; locality: Mengla (Menglun), Jinghong; decimalLatitude: 30.3427; decimalLongitude: 119.4338; **Identification:** identifiedBy: Xie Yanlan; dateIdentified: 2019; identificationReferences: (ThripsWiki 2020); **Event:** samplingProtocol: sweeping and shaking; eventDate: 30/05/2018, 16-18/05/2019; **Record Level:** collectionID: thrips; institutionCode: YAU5082020; collectionCode: terebrantia; basisOfRecord: preserved specimen

##### Ecological interactions

###### Feeds on

leaves and collected from tea tree, lotus, wide plant varieties.

##### Distribution

Described from Grenada. Recorded from Florida USA, widely around the Caribbean, also Japan, Taiwan, Malaysia, Australia, Fiji (Mound and Marullo 1996) and China.

##### Diagnosis

**Figure 3.**
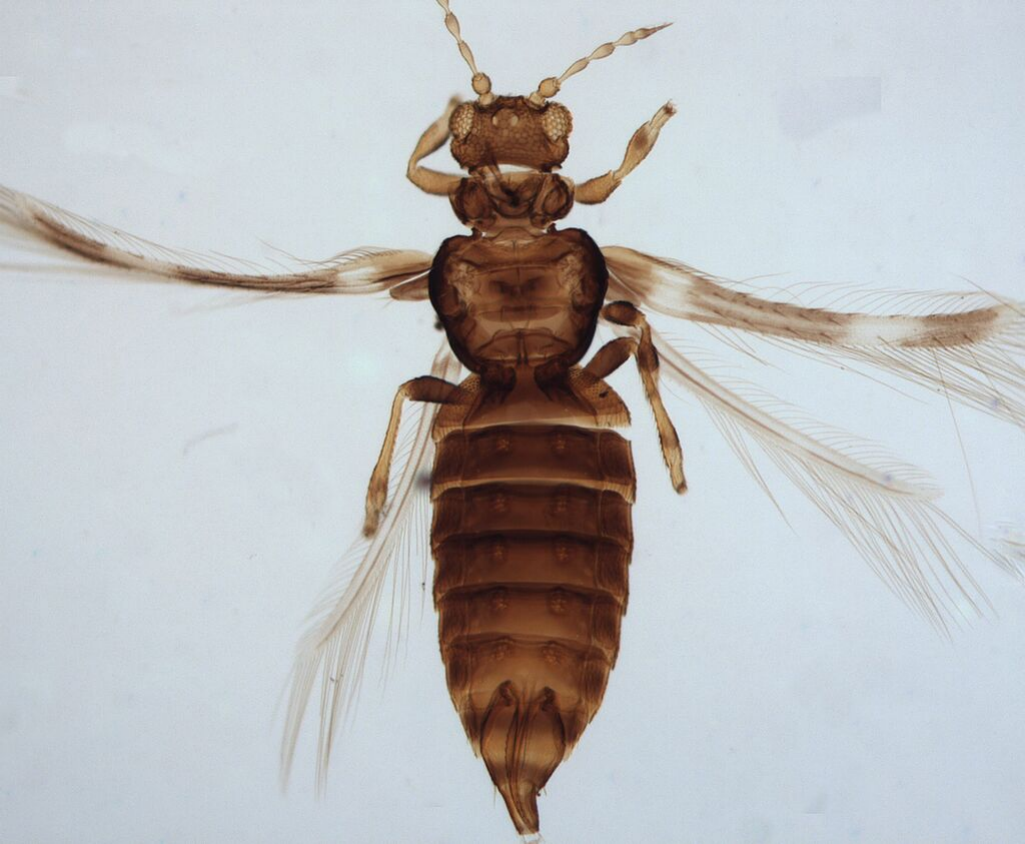
Female of *Anisopilothripsvenustulus* (Priesner, 1923).

Female macropterous; Body brown to yellowish-brown, abdomen often yellow (Fig. 3), antennal segments I-VI largely yellow with brown apices; tarsi yellow, hind tibiae yellow with small brown area, fore wings brown with small pale cross bands sub-basally, medially and at apex; antennae 8-segmented, III and IV with simple sense cone, segment VIII twice as long as VII. Pronotum reticulated; the presence of a complete longitidinal division on the mesonotum; fore wing curved forward at apex with two veins, first vein with about five dark setae and two setae distally, second vein with a row of widely-spaced setae.

#### Araliacothrips daweishanensis

Li, Li & Zhang, 2018

A767F462-D5BA-5D5F-B5EF-14E9459E61D2

https://thrips.info/wiki/Araliacothrips_daweishanensis

##### Materials

- **Type status:** Other material. **Occurrence:** recordedBy: X.Y.L; individualID: 2019-v-16; individualCount: 3; sex: 1 male, 2 females; lifeStage: adults; occurrenceID: YAU5082020Tt3; **Taxon:** scientificNameAuthorship: *Araliacothripsdaweishanensis* Li, Li & Zhang; **Location:** country: China; stateProvince: Yunnan; municipality: Xishuangbanna; locality: Menghai; decimalLatitude: 19.1167; decimalLongitude: 109.05; **Identification:** identifiedBy: Elie N. & Li Yajin; dateIdentified: 2019; identificationReferences: (ThripsWiki 2020); **Event:** samplingProtocol: sweeping and shaking; eventDate: 16/05/2019; **Record Level:** collectionID: thrips; institutionCode: YAU5082020; collectionCode: terebrantia; basisOfRecord: preserved specimen

##### Ecological interactions

###### Feeds on

leaves and collected from Ranunculaceae and Polypodiaceae (ferns).

##### Distribution

Described from Xishuangbanna (Li et al. 2018b) and distributed from southern China.

##### Diagnosis

**Figure 4.**
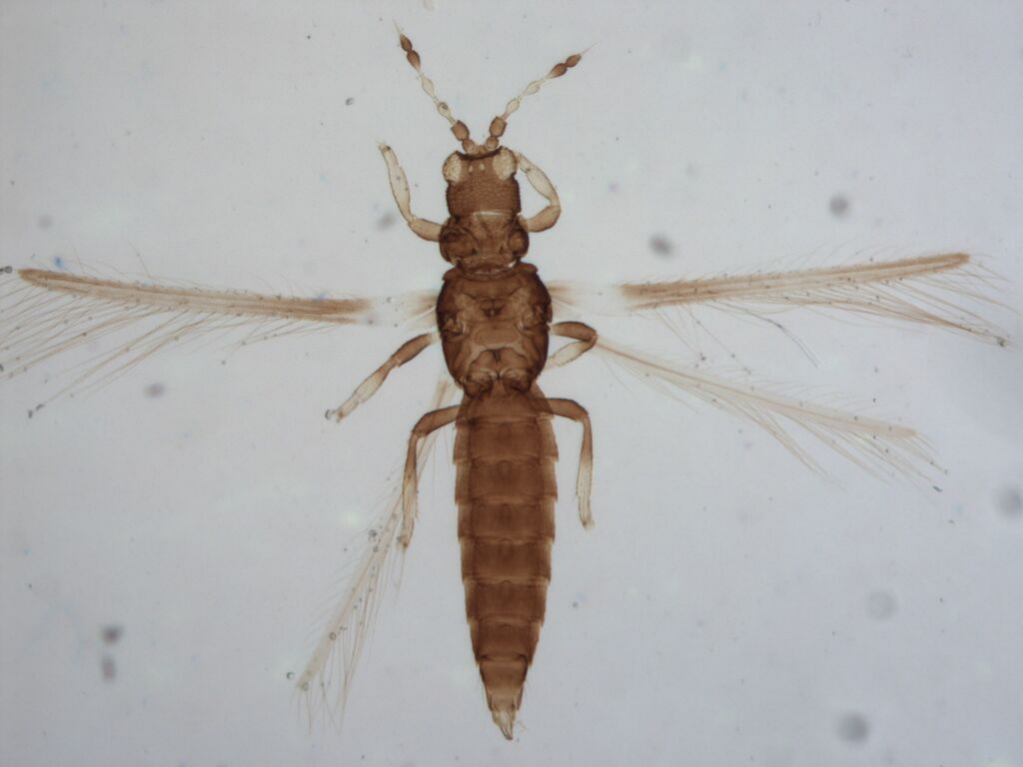
Male of *Araliacothripsdaweishanensis* Li, Li & Zhang, 2018.

Female fully winged; body dark brown and strongly reticulate (Fig. 4); cheeks parallel, constricted behind eyes; maxillary palps 2-segmented; compound eyes with 6 weakly pigmented facets; ocellar setae pair I present, pair III on anterolateral margins of the ocellar triangle; antennae 8-segmented, III and IV with long apical neck, III with sense cone long and forked, IV with one forked and one simple sense cone, VI constricted at base, VIII longer than VII; pronotum reticulated with small setae. Identification details are in the provided link.

#### Astrothrips asiaticus

(Bhatti, 1967)

8151263C-1F8D-58F8-B02B-CEC1B5C0BE11

https://thrips.info/wiki/Astrothrips_asiaticus

Sempothrips asiaticus Bhatti, 1967: 7.

##### Materials

- **Type status:** Other material. **Occurrence:** recordedBy: X.Y.L & Z.H.R; individualID: 2018-vi-1 | 2017-iii-11; individualCount: 3; sex: 1 male, 2 females; lifeStage: adults; occurrenceID: YAU5082020Tt4; **Taxon:** scientificNameAuthorship: *Astrothripsasiaticus* (Bhatti); **Location:** country: China; stateProvince: Yunnan; municipality: Xishuangbanna; locality: Mengla (Menglun); decimalLatitude: 30.3427; decimalLongitude: 119.4338; **Identification:** identifiedBy: Xie Yanlan; dateIdentified: 2018; identificationReferences: (ThripsWiki 2020); **Event:** samplingProtocol: sweeping and shaking; eventDate: 11/03/2017, 01/06/2018; **Record Level:** collectionID: thrips; institutionCode: YAU5082020; collectionCode: terebrantia; basisOfRecord: preserved specimen

##### Ecological interactions

###### Feeds on

leaves and collected from grasses.

##### Distribution

Palaeotropics, from West Africa to northern Australia, Japan and southern China.

##### Diagnosis

**Figure 5.**
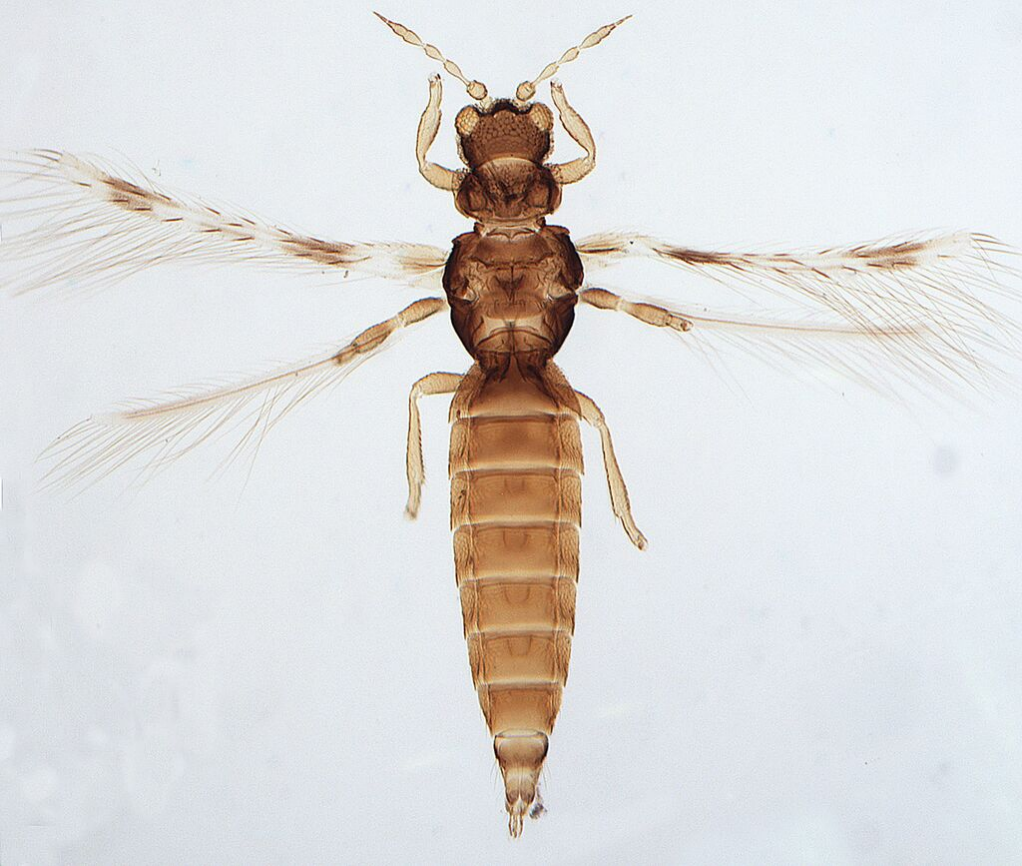
Male of *Astrothripsasiaticus* (Bhatti, 1967).

Macropterous, body dark brown to yellowish with reticulations (Fig. 5); antennal 8-segments, all eight antennal segments are clearly separated, antennal segments III–IV with sense cone simple. Male with U-shaped sternal pore plates.

#### Astrothrips aucubae

Kurosawa, 1932

5F05E675-6BB5-5353-8E81-2441B187DE52

https://thrips.info/wiki/Astrothrips_aucubae

##### Materials

- **Type status:** Other material. **Occurrence:** recordedBy: L.Y.J & X.Y.L.; individualID: 2017-X-21|2017-III-10|2017-VIII-7; individualCount: 7; sex: 3 males, 4 females; lifeStage: adults; occurrenceID: YAU5082020Tt5; **Taxon:** scientificNameAuthorship: *Astrothripsaucubae* Kurosawa; **Location:** country: China; stateProvince: Yunnan; municipality: Xishuangbanna; locality: Mengla (Menglun); decimalLatitude: 22.011754; decimalLongitude: 100.785957; **Identification:** identifiedBy: Xie Yanlan; dateIdentified: 2018; identificationReferences: (ThripsWiki 2020); **Event:** samplingProtocol: sweeping and shaking; eventDate: 10/03/2017, 07/08/2017, 21/10/2017; **Record Level:** collectionID: thrips; institutionCode: YAU5082020; collectionCode: terebrantia; basisOfRecord: preserved specimen

##### Ecological interactions

###### Feeds on

leaves and collected from *Puerarialobata* and *Ficus* tree.

##### Distribution

Described from Japan. Recorded from China (Li et al. 2021).

#### Astrothrips aureolus

Stannard & Mitri, 1962

F56DDA33-274A-545F-B0DF-12A1FEFC9F07

https://thrips.info/wiki/Astrothrips_aureolus

##### Materials

- **Type status:** Other material. **Occurrence:** recordedBy: L.Y.J; individualID: 2017-III-11; individualCount: 3; sex: females; lifeStage: adults; occurrenceID: YAU5082020Tt6; **Taxon:** scientificNameAuthorship: *Astrothripsaureolus* Stannard & Mitri; **Location:** country: China; stateProvince: Yunnan; municipality: Xishuangbanna; locality: Jinghong; decimalLatitude: 22.001969; decimalLongitude: 100.795012; **Identification:** identifiedBy: Xie Yanlan; dateIdentified: 2018; identificationReferences: (ThripsWiki 2020); **Event:** samplingProtocol: sweeping and shaking; eventDate: 11/03/2017; **Record Level:** collectionID: thrips; institutionCode: YAU5082020; collectionCode: terebrantia; basisOfRecord: preserved specimen

##### Ecological interactions

###### Feeds on

leaves and collected from *Ophiopogonjaponicus* (Asparagaceae).

##### Distribution

Described from Peninsular Malaysia. Recorded from northern Australia and southern China.

#### Astrothrips chisinliaoensis

Chen, 1980

AC98D96A-CA5F-58DC-AB65-F633E6B4D0F9

https://thrips.info/wiki/Astrothrips_chisinliaoensis

##### Materials

- **Type status:** Other material. **Occurrence:** recordedBy: L.Y.J; individualID: 2017-III-11; individualCount: 4; sex: females; lifeStage: adults; occurrenceID: YAU5082020Tt7; **Taxon:** scientificNameAuthorship: *Astrothripschisinliaoensis* Chen; **Location:** country: China; stateProvince: Yunnan; municipality: Xishuangbanna; locality: Mengla (Menglun); decimalLatitude: 21.963829; decimalLongitude: 100.64345; **Identification:** identifiedBy: Xie Yanlan; dateIdentified: 2018; identificationReferences: (ThripsWiki 2020); **Event:** samplingProtocol: sweeping and shaking; eventDate: 11/03/2017; **Record Level:** collectionID: thrips; institutionCode: YAU5082020; collectionCode: terebrantia; basisOfRecord: preserved specimen

##### Ecological interactions

###### Feeds on

leaves, collected from ferns and *Ternstroemiapseudoverticillata*.

##### Distribution

Described from Republic of Moldova (Chisinliao). Recorded from southern China and Taiwan.

#### Astrothrips globiceps

(Karny, 1913)

C6CFFBB5-8CB0-5905-90E8-84AA89EBCBFF

https://thrips.info/wiki/Astrothrips_globiceps

Heliothrips globiceps Karny, 1913: 125.

##### Materials

- **Type status:** Other material. **Occurrence:** recordedBy: Z.H.R; individualID: 2016-X-25; individualCount: 2; sex: males; lifeStage: adults; occurrenceID: YAU5082020Tt8; **Taxon:** scientificNameAuthorship: *Astrothripsglobiceps* (Karny); **Location:** country: China; stateProvince: Yunnan; municipality: Xishuangbanna; locality: Jinghong; decimalLatitude: 22.043353; decimalLongitude: 100.917923; **Identification:** identifiedBy: Xie Yanlan; dateIdentified: 2018; identificationReferences: (ThripsWiki 2020); **Event:** samplingProtocol: sweeping and shaking; eventDate: 25/10/2016; **Record Level:** collectionID: thrips; institutionCode: YAU5082020; collectionCode: terebrantia; basisOfRecord: preserved specimen

##### Ecological interactions

###### Feeds on

leaves and collected from onion.

##### Distribution

Described from Indonesia, New Britain, Papua New Guinea. Recorded from China.

#### Astrothrips strasseni

Kudo, 1979

D7CBD7F4-FABA-5F80-98D5-390F31769E2F

https://thrips.info/wiki/Astrothrips_strasseni

##### Materials

- **Type status:** Other material. **Occurrence:** recordedBy: E.N & X.Y.L; individualID: 2019-v-16; individualCount: 6; sex: 1 male, 5 females; lifeStage: adults; occurrenceID: YAU5082020Tt9; **Taxon:** scientificNameAuthorship: *Astrothripsstrasseni* Kudo; **Location:** country: China; stateProvince: Yunnan; municipality: Xishuangbanna; locality: Menghai (Protected Area); decimalLatitude: 22.010116; decimalLongitude: 100.958167; **Identification:** identifiedBy: Xie Yanlan; dateIdentified: 2019; identificationReferences: (ThripsWiki 2020); **Event:** samplingProtocol: sweeping and shaking; eventDate: 16/05/2019; **Record Level:** collectionID: thrips; institutionCode: YAU5082020; collectionCode: terebrantia; basisOfRecord: preserved specimen

##### Ecological interactions

###### Feeds on

leaves and collected from bamboo.

##### Distribution

Described from Myanmar and recorded from southern China.

#### Astrothrips tumiceps

Karny, 1923

4BFB83B6-AF86-5F2C-AE4C-75634C525CC9

https://thrips.info/wiki/Astrothrips_tumiceps

##### Materials

- **Type status:** Other material. **Occurrence:** recordedBy: X.Y.L & Z.H.R; individualID: 2018-vi-1|2017-iii-11; individualCount: 7; sex: 1 male, 6 females; lifeStage: adults; occurrenceID: YAU5082020Tt10; **Taxon:** scientificNameAuthorship: *Astrothripstumiceps* Karny; **Location:** country: China; stateProvince: Yunnan; municipality: Xishuangbanna; locality: Mengla (Menglun); decimalLatitude: 22.004755; decimalLongitude: 100.922522; **Identification:** identifiedBy: Xie Yanlan; dateIdentified: 2018; identificationReferences: (ThripsWiki 2020); **Event:** samplingProtocol: sweeping and shaking; eventDate: 11/03/2017, 01/06/2018; **Record Level:** collectionID: thrips; institutionCode: YAU5082020; collectionCode: terebrantia; basisOfRecord: preserved specimen

##### Ecological interactions

###### Feeds on

leaves and collected from Moringa and Smilacaceae.

##### Distribution

Described from Indonesia. Recorded from India, Philippines, northern Australia and China.

##### Diagnosis

**Figure 6.**
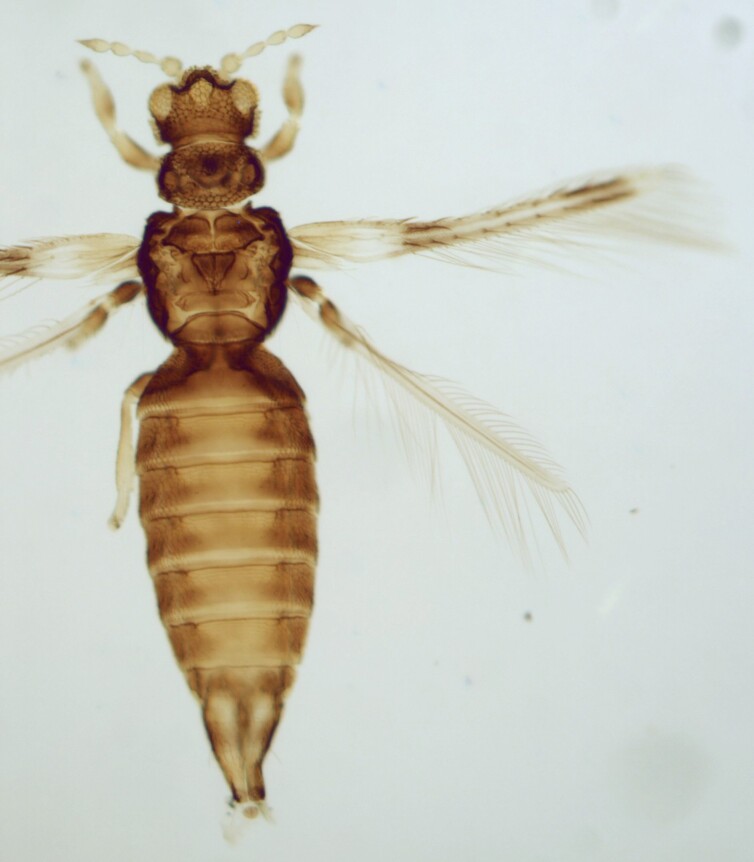
Female of *Astrothripstumiceps* Karny, 1923.

This species differs from *A.asiaticus* by antennae with 5 to 7 segments; metanotum triangle of reticulation sharply defined; mesonotum anterior third fully divided with no sculptured reticulate connection (Fig. 6); male with no sternal pore plates.

#### Caliothrips tongi

Mound, Zhang & Bei, 2011

F18A0D01-D4D0-54EA-A38E-F47ECEF4D3E1

https://thrips.info/wiki/Caliothrips_tongi

##### Materials

- **Type status:** Other material. **Occurrence:** recordedBy: L.H; individualID: 2017-viii-10; individualCount: 6; sex: 2 males, 4 females; lifeStage: adults; occurrenceID: YAU5082020Tt11; **Taxon:** scientificNameAuthorship: *Caliothripstongi* Mound Zhang & Bei; **Location:** country: China; stateProvince: Yunnan; municipality: Xishuangbanna; locality: Mengla (Menglun); decimalLatitude: 21.995104; decimalLongitude: 100.879979; **Identification:** identifiedBy: Xie Yanlan; dateIdentified: 2018; identificationReferences: (ThripsWiki 2020); **Event:** samplingProtocol: sweeping and shaking; eventDate: 10/07/2017; **Record Level:** collectionID: thrips; institutionCode: YAU5082020; collectionCode: terebrantia; basisOfRecord: preserved specimen

##### Ecological interactions

###### Feeds on

leaves and collected from a wide range of host plants.

##### Distribution

Described from Zhejiang Province, China (Mound et al. 2011).

#### Copidothrips octarticulatus

(Schmutz, 1913)

C30DBC32-C4D9-5B70-B058-0E2426E9BD2F

https://thrips.info/wiki/Copidothrips_octarticulatus

Heliothrips (Parthenothrips) octarticulata Schmutz, 1913: 993
Copidothrips formosus Hood, 1954: 190
Mesostenothrips kraussi Stannard & Mitri, 1962: 211.

##### Materials

- **Type status:** Other material. **Occurrence:** recordedBy: L.Y.J & X.Y.L.; individualID: 2017-X-25|2018-v-31|2018-vi-1; individualCount: 27; sex: 1 male, 26 female; lifeStage: adults; occurrenceID: YAU5082020Tt12; **Taxon:** scientificNameAuthorship: *Copidothripsoctarticulatus* (Schmutz); **Location:** country: China; stateProvince: Yunnan; municipality: Xishuangbanna; locality: Mengla (Tropical Forest); decimalLatitude: 21.973654; decimalLongitude: 100.942069; **Identification:** identifiedBy: Xie Yanlan; dateIdentified: 2018; identificationReferences: (ThripsWiki 2020); **Event:** samplingProtocol: sweeping and shaking; eventDate: 25/10/2017, 01/05/2018, 31/05/2018; **Record Level:** collectionID: thrips; institutionCode: YAU5082020; collectionCode: terebrantia; basisOfRecord: preserved specimen

##### Ecological interactions

###### Feeds on

leaves and collected from asparagus and ferns.

##### Distribution

Described from Sri Lanka. Recorded from Taiwan, Gilbert Island, Kiribati, Seychelles, Thailand, northern Australia and southern China.

##### Diagnosis

**Figure 7.**
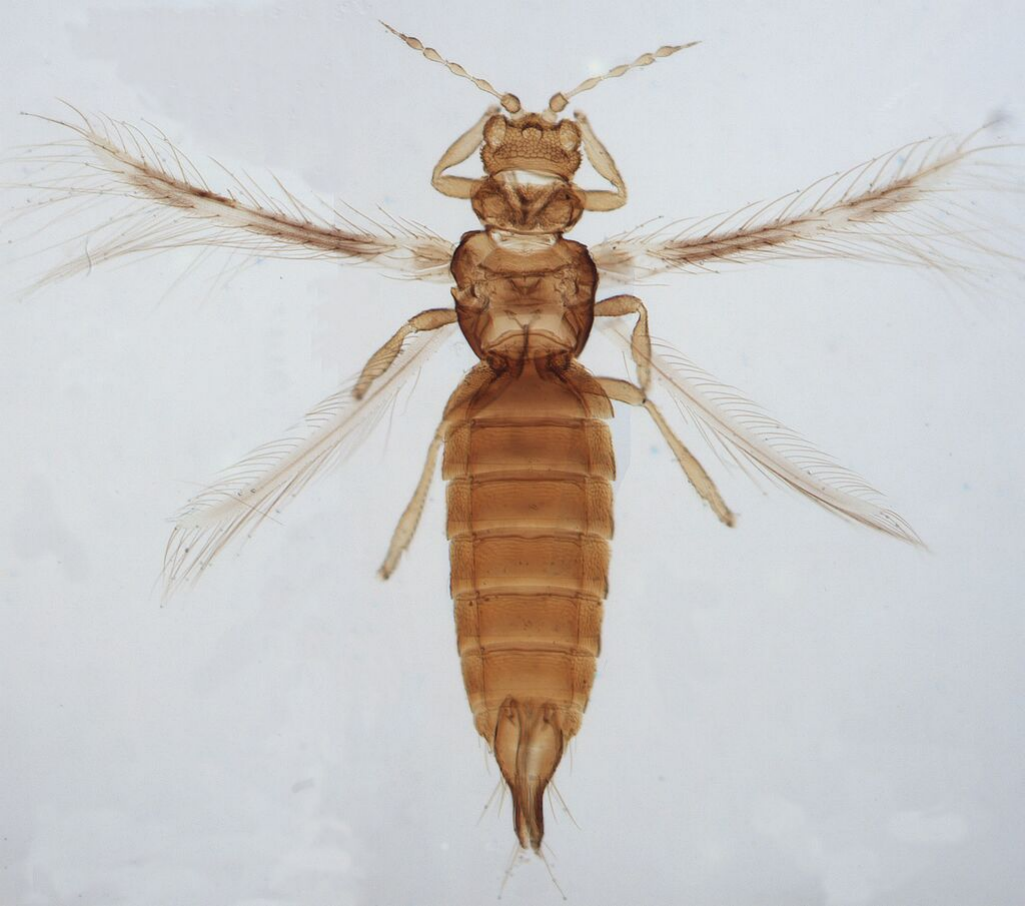
Female of *Copidothripsoctarticulatus* (Schmutz, 1913).

Female fully winged; body yellowish-brown (Fig. 7), pterothorax darker laterally; antennal segments I, III–V and basal half of VI yellow, II, VII and apex of VI brown; tarsi and tibiae yellow; fore wing brown with transverse white bands sub-basally, medially and at the apex; head with cheeks convex and constricted at base; ocellar region elevated; antennae 8-segmented, III and IV with simple sensorium, VIII twice as long as VII. Pronotum with six pairs of large setae.

#### Helionothrips brunneipennis

Bagnall, 1915

4D994E0D-128C-5ADC-BEDC-8F90DDB91D0C

https://thrips.info/wiki/Helionothrips_brunneipennis

##### Materials

- **Type status:** Other material. **Occurrence:** recordedBy: X.Y.L; individualID: 2018-v-30; individualCount: 1; sex: female; lifeStage: adults; occurrenceID: YAU5082020Tt13; **Taxon:** scientificNameAuthorship: *Helionothripsbrunneipennis* Bagnall; **Location:** country: China; stateProvince: Yunnan; municipality: Xishuangbanna; locality: Mengla (Menglun); decimalLatitude: 22.005827; decimalLongitude: 100.924822; **Identification:** identifiedBy: Xie Yanlan; dateIdentified: 2018; identificationReferences: (ThripsWiki 2020); **Event:** samplingProtocol: sweeping and shaking; eventDate: 30/05/2018; **Record Level:** collectionID: thrips; institutionCode: YAU5082020; collectionCode: terebrantia; basisOfRecord: preserved specimen

##### Ecological interactions

###### Feeds on

leaves and collected from *Reinwardtiaindica* (Lauraceae).

##### Distribution

Described from Sri Lanka and recorded from China.

#### Helionothrips cephalicus

Hood, 1954

069A8A93-828A-563E-8DD3-70B6557C3E6C

https://thrips.info/wiki/Helionothrips_cephalicus

##### Materials

- **Type status:** Other material. **Occurrence:** recordedBy: L.Y.J. & Z.H.R; individualID: 2017-iii-11; individualCount: 5; sex: 2 males, 3 females; lifeStage: adults; occurrenceID: YAU5082020Tt14; **Taxon:** scientificNameAuthorship: *Helionothripscephalicus* Hood; **Location:** country: China; stateProvince: Yunnan; municipality: Xishuangbanna; locality: Mengla (Tropical Forest); decimalLatitude: 22.109802; decimalLongitude: 100.87078; **Identification:** identifiedBy: Xie Yanlan; dateIdentified: 2018; identificationReferences: (ThripsWiki 2020); **Event:** samplingProtocol: sweeping and shaking; eventDate: 11/03/2017; **Record Level:** collectionID: thrips; institutionCode: YAU5082020; collectionCode: terebrantia; basisOfRecord: preserved specimen

##### Ecological interactions

###### Feeds on

leaves and collected from Anacardiaceae and Poaceae.

##### Distribution

Described from Japan and recorded from China (Sichuan, Taiwan and Yunnan).

##### Diagnosis

**Figure 8.**
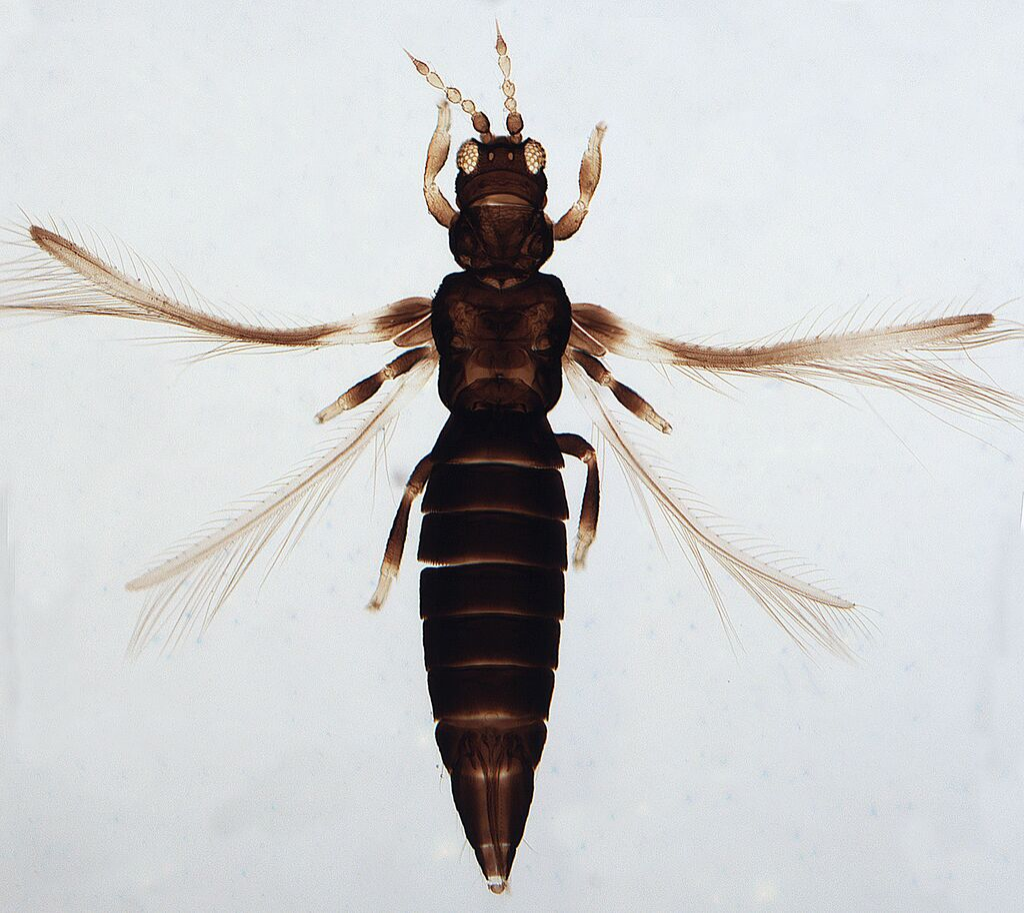
Female of *Helionothripscephalicus* Hood, 1954.

Female fully winged; body dark brown (Fig. 8); head wider than long, strongly reticulate, not projecting in front of eyes; ocellar region elevated, occipital ridge present; two pairs of postocular setae; maxillary palps 2-segmented; antennal segment II prominently darker than VI; male pore glands on sternite VIII only.

#### Helionothrips mube

Kudo, 1992

AD196B39-D356-5E86-931A-868A0B0F6F43

https://thrips.info/wiki/Helionothrips_mube

##### Materials

- **Type status:** Other material. **Occurrence:** recordedBy: Z.H.R; individualID: 2017-iii-10; individualCount: 5; sex: 2 males, 3 females; lifeStage: adults; occurrenceID: YAU5082020Tt15; **Taxon:** scientificNameAuthorship: *Helionothripsmube* Kudo; **Location:** country: China; stateProvince: Yunnan; municipality: Xishuangbanna; locality: Jinghong (Protected Area); decimalLatitude: 22.177291; decimalLongitude: 100.890327; **Identification:** identifiedBy: Xie Yanlan; dateIdentified: 2018; identificationReferences: (ThripsWiki 2020); **Event:** samplingProtocol: sweeping and shaking; eventDate: 10/03/2017; **Record Level:** collectionID: thrips; institutionCode: YAU5082020; collectionCode: terebrantia; basisOfRecord: preserved specimen

##### Ecological interactions

###### Feeds on

leaves and collected from taro, papaya, Poaceae, vines.

##### Distribution

Described from Japan (Nagasaki, Inasayama) and recorded from China (Shanxi, Taiwan and Yunnan).

#### Helionothrips parvus

Bhatti, 1968

900C80D2-9A10-531B-AC55-A1207019C798

https://thrips.info/wiki/Helionothrips_parvus

##### Materials

- **Type status:** Other material. **Occurrence:** recordedBy: L.Y.J; individualID: 2017-iii-11; individualCount: 7; sex: 2 males, 6 females; lifeStage: adults; occurrenceID: YAU5082020Tt16; **Taxon:** scientificNameAuthorship: *Helionothripsparvus* Bhatti; **Location:** country: China; stateProvince: Yunnan; municipality: Xishuangbanna; locality: Mengla (Menglun); decimalLatitude: 22.177291; decimalLongitude: 100.861869; **Identification:** identifiedBy: Xie Yanlan; dateIdentified: 2018; identificationReferences: (ThripsWiki 2020); **Event:** samplingProtocol: sweeping and shaking; eventDate: 11/03/2017; **Record Level:** collectionID: thrips; institutionCode: YAU5082020; collectionCode: terebrantia; basisOfRecord: preserved specimen

##### Ecological interactions

###### Feeds on

leaves, collected from Poaceae and Ferns.

##### Distribution

Described from India and recorded from China (Guangxi, Yunnan).

#### Helionothrips rugatus

Mirab-balou & Tong, 2016

A60CF5FA-7563-5F40-8DAF-29DDF757130C

https://thrips.info/wiki/Helionothrips_rugatus

##### Materials

- **Type status:** Other material. **Occurrence:** recordedBy: E.N, L.Y.J, X.Y.L & Z.H.R; individualID: 2018-vi-1|2018-v-31|2017-iii-24|2017-x-24; individualCount: 24; sex: 6 males, 18 female; lifeStage: adults; occurrenceID: YAU5082020Tt17; **Taxon:** scientificNameAuthorship: *Helionothripsrugatus* Mirab-balou & Tong; **Location:** country: China; stateProvince: Yunnan; municipality: Xishuangbanna; locality: Jinghong (Nabanhe Protected Area), Mengla (Menglun); decimalLatitude: 22.179968; decimalLongitude: 100.849508; **Identification:** identifiedBy: Xie Yanlan; dateIdentified: 2018; identificationReferences: (ThripsWiki 2020); **Event:** samplingProtocol: sweeping and shaking; eventDate: 24/03/2017, 24/10/2017, 31/05/2018, 01/06/2018; **Record Level:** collectionID: thrips; institutionCode: YAU5082020; collectionCode: terebrantia; basisOfRecord: preserved specimen

##### Ecological interactions

###### Feeds on

leaves and collected from Poaceae, Cornaceae, blackberries, ferns, *Lophatherumgracile* and *Pueraria*.

##### Distribution

Described from Guangdong, distributed from Guangxi and Yunnan Provinces of China.

#### Helionothrips shennongjiaensis

Feng, Yang & Zhang, 2007

380490CE-3EB7-5A2F-B941-E5FF649DED2F

https://thrips.info/wiki/Helionothrips_shennongjiaensis

##### Materials

- **Type status:** Other material. **Occurrence:** recordedBy: X.Y.L; individualID: 2017-x-24; individualCount: 13; sex: 5 males, 8 females; lifeStage: adults; occurrenceID: YAU5082020Tt18; **Taxon:** scientificNameAuthorship: *Helionothripsshennongjiaensis* Feng, Yang & Zhang; **Location:** country: China; stateProvince: Yunnan; municipality: Xishuangbanna; locality: Mengla (Tropical Rain Forest); decimalLatitude: 21.922967; decimalLongitude: 101.184971; **Identification:** identifiedBy: Xie Yanlan; dateIdentified: 2018; identificationReferences: (ThripsWiki 2020); **Event:** samplingProtocol: sweeping and shaking; eventDate: 24/10/2017; **Record Level:** collectionID: thrips; institutionCode: YAU5082020; collectionCode: terebrantia; basisOfRecord: preserved specimen

##### Ecological interactions

###### Feeds on

leaves, collected from wide host plants.

##### Distribution

Described from Hubai China and distributed from Yunnan, Guangdong and Hainan Provinces (Feng et al. 2007).

#### Heliothrips longisensibilis

Xie, Mound & Zhang, 2019

8748008E-5DB1-5920-870D-DA5138C69E53

https://thrips.info/wiki/Heliothrips_longisensibilis

##### Materials

- **Type status:** Other material. **Occurrence:** recordedBy: L.H & X.Y.L; individualID: 2017-iii-11|2018-v-30|2017-x-24; individualCount: 12; sex: 3 males, 9 females; lifeStage: adults; occurrenceID: YAU5082020Tt19; **Taxon:** scientificNameAuthorship: *Heliothripslongisensibilis* Xie, Mound & Zhang; **Location:** country: China; stateProvince: Yunnan; municipality: Xishuangbanna; locality: Mengla (Menglun); decimalLatitude: 21.918541; decimalLongitude: 101.184828; **Identification:** identifiedBy: Xie Yanlan; dateIdentified: 2018; identificationReferences: (ThripsWiki 2020); **Event:** samplingProtocol: sweeping and shaking; eventDate: 11/03/2017, 24/03/2017, 30/05/2018; **Record Level:** collectionID: thrips; institutionCode: YAU5082020; collectionCode: terebrantia; basisOfRecord: preserved specimen

##### Ecological interactions

###### Feeds on

leaves, collected from tea, *Pinus*, ferns, *Tecomastans*.

##### Distribution

Described from Xishuangbanna, Yunnan Province, China (Xie et al. 2019).

#### Panchaetothrips bifurcus

Mirab-balou & Tong, 2016

1E2A577C-0B0E-5A4E-B2FB-BFAEC9D763DA

https://thrips.info/wiki/Panchaetothrips_bifurcus

##### Materials

- **Type status:** Other material. **Occurrence:** recordedBy: X.Y.L & E.N; individualID: 2018-Vi-2; individualCount: 9; sex: 3 males, 6 females; lifeStage: adults; occurrenceID: YAU5082020Tt20; **Taxon:** scientificNameAuthorship: *Panchaetothripsbifurcus* Mirab-balou & Tong; **Location:** country: China; stateProvince: Yunnan; municipality: Xishuangbanna; locality: Mengla (Tropical Rain Forest); decimalLatitude: 21.914651; decimalLongitude: 101.186983; **Identification:** identifiedBy: Xie Yanlan; dateIdentified: 2018; identificationReferences: (ThripsWiki 2020); **Event:** samplingProtocol: sweeping and shaking; eventDate: 02/06/2018; **Record Level:** collectionID: thrips; institutionCode: YAU5082020; collectionCode: terebrantia; basisOfRecord: preserved specimen

##### Ecological interactions

###### Feeds on

leaves, collected from Poaceae, Cornaceae, blackberries, ferns and *Pueraria*.

##### Distribution

Described from Hainan, distributed from Guangdong and Jiangxi Provinces, China.

##### Diagnosis

**Figure 9.**
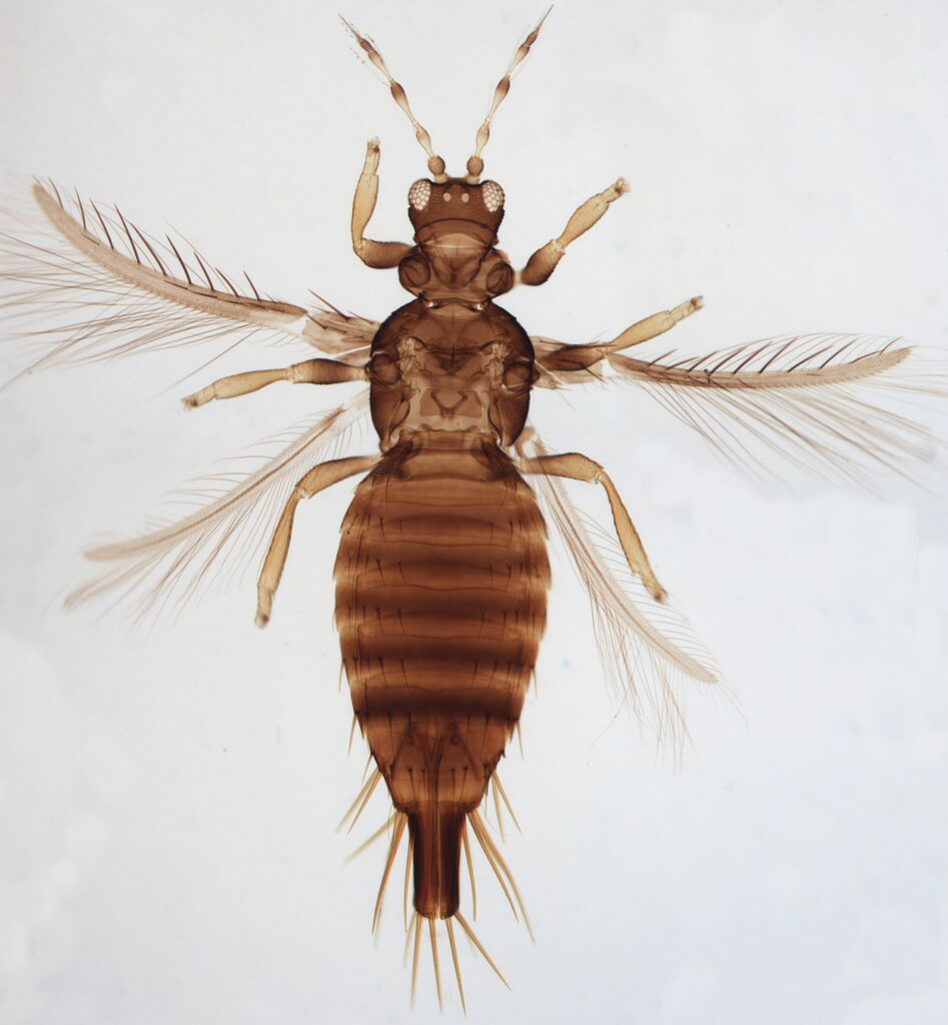
Female of *Panchaetothripsbifurcus* Mirab-balou & Tong, 2016.

Female fully winged; body dark brown (Fig. 9); head longer than wide, strongly reticulate, not projecting in front of eyes; two pairs of postocular setae; maxillary palps 2-segmented. Antennae 8-segmented, segment I without paired dorso-apical setae; III and IV with simple sense cones or forked, IV with or without extra simple sense cone. Pronotum transversely reticulate, no long setae; mesonotum reticulate without anteromedian campaniform sensilla.

#### Panchaetothrips indicus

Bagnall, 1912

6977E42E-F865-547E-B359-E08F8541262F

https://thrips.info/wiki/Panchaetothrips_indicus

##### Materials

- **Type status:** Other material. **Occurrence:** recordedBy: X.Y.L & S.J; individualID: 2011-ix-30; individualCount: 18; sex: females; lifeStage: adults; occurrenceID: YAU5082020Tt21; **Taxon:** scientificNameAuthorship: *Panchaetothripsindicus* Bagnall; **Location:** country: China; stateProvince: Yunnan; municipality: Xishuangbanna; locality: Jinghong (Protected Area); decimalLatitude: 21.916663; decimalLongitude: 101.193451; **Identification:** identifiedBy: Xie Yanlan; dateIdentified: 2018; identificationReferences: (ThripsWiki 2020); **Event:** samplingProtocol: sweeping and shaking; eventDate: 30/09/2011; **Record Level:** collectionID: thrips; institutionCode: YAU5082020; collectionCode: terebrantia; basisOfRecord: preserved specimen

##### Ecological interactions

###### Feeds on

leaves and flowers, collected from coffee trees, bananas and bamboo.

##### Distribution

Described from India and recorded from China.

#### Phibalothrips peringueyi

(Faure, 1925)

B02B2801-0572-583A-9C51-89D40848D16C

https://thrips.info/wiki/Phibalothrips_peringueyi

Reticulothrips peringueyi Faure, 1925: 145.

##### Materials

- **Type status:** Other material. **Occurrence:** recordedBy: T.X.L; individualID: 2018-viii-2|2018-v-31; individualCount: 3; sex: females; lifeStage: adults; occurrenceID: YAU5082020Tt22; **Taxon:** scientificNameAuthorship: *Phibalothripsperingueyi* (Faure); **Location:** country: China; stateProvince: Yunnan; municipality: Xishuangbanna; locality: Mengla (Menglun); decimalLatitude: 21.923101; decimalLongitude: 101.200925; **Identification:** identifiedBy: Xie Yanlan; dateIdentified: 2018; identificationReferences: (ThripsWiki 2020); **Event:** samplingProtocol: sweeping and shaking; eventDate: 31/05/2018, 02/08/2018; **Record Level:** collectionID: thrips; institutionCode: YAU5082020; collectionCode: terebrantia; basisOfRecord: preserved specimen

##### Ecological interactions

###### Feeds on

leaves and flowers, collected from Poaceae and bamboo.

##### Distribution

Described from South Africa, recorded from Taiwan and south China.

#### Phibalothrips rugosus

Kudo, 1979

E1BC5E64-C044-56D8-98A0-6F9557D796B8

https://thrips.info/wiki/Phibalothrips_rugosus

##### Materials

- **Type status:** Other material. **Occurrence:** recordedBy: E.N. & Z.H.R; individualID: 2018-viii-2|2018-v-3; individualCount: 6; sex: 2 males, 4 females; lifeStage: adults; occurrenceID: YAU5082020Tt23; **Taxon:** scientificNameAuthorship: *Phibalothripsrugosus* Kudo; **Location:** country: China; stateProvince: Yunnan; municipality: Xishuangbanna; locality: Mengla (Tropical Botanical Garden); decimalLatitude: 21.919882; decimalLongitude: 101.191583; **Identification:** identifiedBy: Li Yajin; dateIdentified: 2018; identificationReferences: (ThripsWiki 2020); **Event:** samplingProtocol: sweeping and shaking; eventDate: 03/05/2018, 02/08/2018; **Record Level:** collectionID: thrips; institutionCode: YAU5082020; collectionCode: terebrantia; basisOfRecord: preserved specimen

##### Ecological interactions

###### Feeds on

leaves, collected from Poaceae and bamboo.

##### Distribution

Described from Malaysia and recorded from Japan and China (Xishuangbanna).

##### Notes

Newly recorded for China.

##### Diagnosis

**Figure 10.**
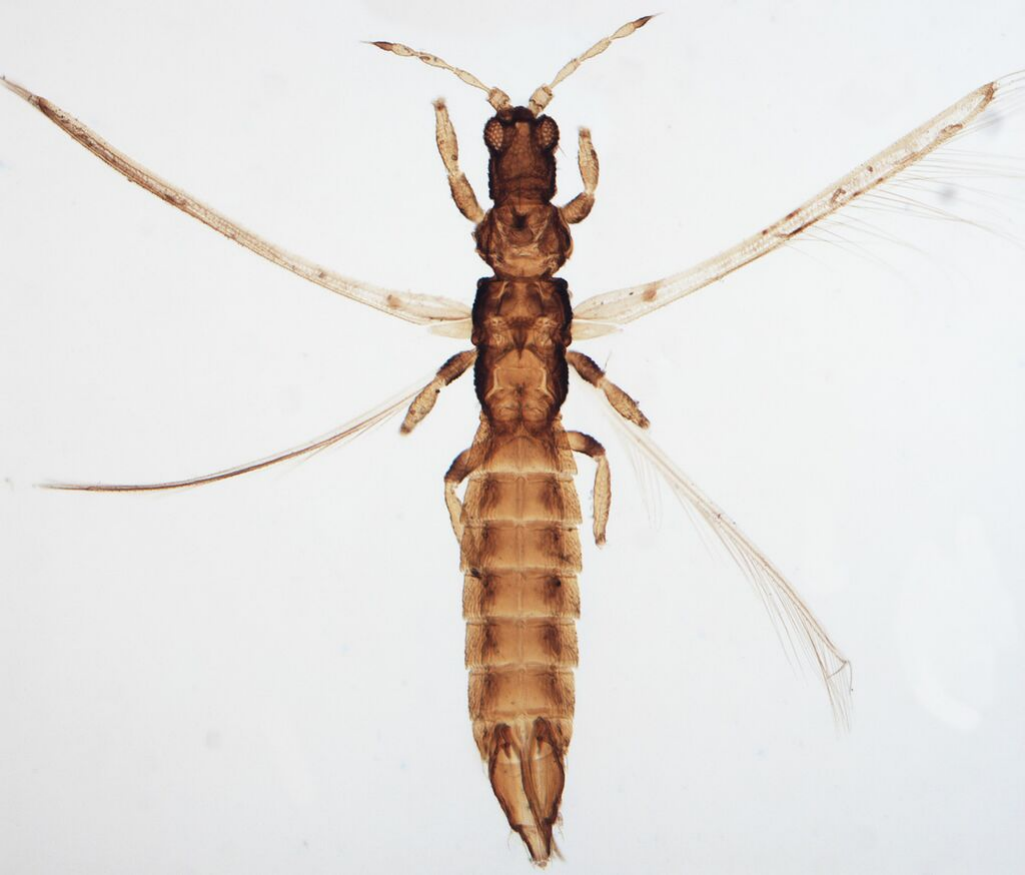
Female of *Phibalothripsrugosus* Kudo, 1979.

Female macropterous; body bicoloured (Fig. 10), head and thorax dark brown and constricted behind eye, abdomen yellow; legs and antennal segments III–V yellow; fore wing slender, uniformly pale or a little darker at base, with no long setae; antennae 6-segmented, V–VII form single unit; sensoria on III and IV slender, each with one simple sensorium. Male similar to female, but smaller, without pore plate at sternites III–VII. The distinctive feature of this genus is an elongated head that is strongly irregular, circular reticulated and constricted behind the eyes.

#### Rhipiphorothrips concoloratus

Zhang & Tong, 1993

8255B3E4-8B1E-56F9-87A3-97275BAB91E4

https://thrips.info/wiki/Rhipiphorothrips_concoloratus

##### Materials

- **Type status:** Other material. **Occurrence:** recordedBy: Z.W.Q; individualID: 1987-iv-11; individualCount: 2; sex: 1male, 1 female; lifeStage: adults; occurrenceID: YAU5082020Tt24; **Taxon:** scientificNameAuthorship: *Rhipiphorothripsconcoloratus* Zhang & Tong; **Location:** country: China; stateProvince: Yunnan; municipality: Xishuangbanna; locality: Mengla (Menglun); decimalLatitude: 21.935842; decimalLongitude: 101.248356; **Identification:** identifiedBy: Xie Yanlan; dateIdentified: 2018; identificationReferences: (ThripsWiki 2020); **Event:** samplingProtocol: sweeping and shaking; eventDate: 11/04/1987; **Record Level:** collectionID: thrips; institutionCode: YAU5082020; collectionCode: terebrantia; basisOfRecord: preserved specimen

##### Ecological interactions

###### Feeds on

leaves collected from hibiscus and grape.

##### Distribution

Described from southern China (Xishuangbanna).

#### Rhipiphorothrips cruentatus

Hood, 1919

C8C7676A-08F8-5753-9F12-07463C8414FC

https://thrips.info/wiki/Rhipiphorothrips_cruentatus

Rhipiphorothrips cruentatus Hood, 1919: 94
Rhipiphorothrips Karna Ramakrishna, 1928: 252.

##### Materials

- **Type status:** Other material. **Occurrence:** recordedBy: W.Q.L; individualID: 1998-ix-22; individualCount: 1; sex: female; lifeStage: adults; occurrenceID: YAU5082020Tt25; **Taxon:** scientificNameAuthorship: *Rhipiphorothripscruentatus* Hood; **Location:** country: China; stateProvince: Yunnan; municipality: Xishuangbanna; locality: Mengla (Tropical Botanical Garden); decimalLatitude: 21.926454; decimalLongitude: 101.254249; **Identification:** identifiedBy: Xie Yanlan; dateIdentified: 2018; identificationReferences: (ThripsWiki 2020); **Event:** samplingProtocol: sweeping and shaking; eventDate: 22/09/1998; **Record Level:** collectionID: thrips; institutionCode: YAU5082020; collectionCode: terebrantia; basisOfRecord: preserved specimen

##### Ecological interactions

###### Feeds on

leaves and collected from grape.

##### Distribution

Described from India and recorded from China (Xishuangbanna).

##### Diagnosis

**Figure 11.**
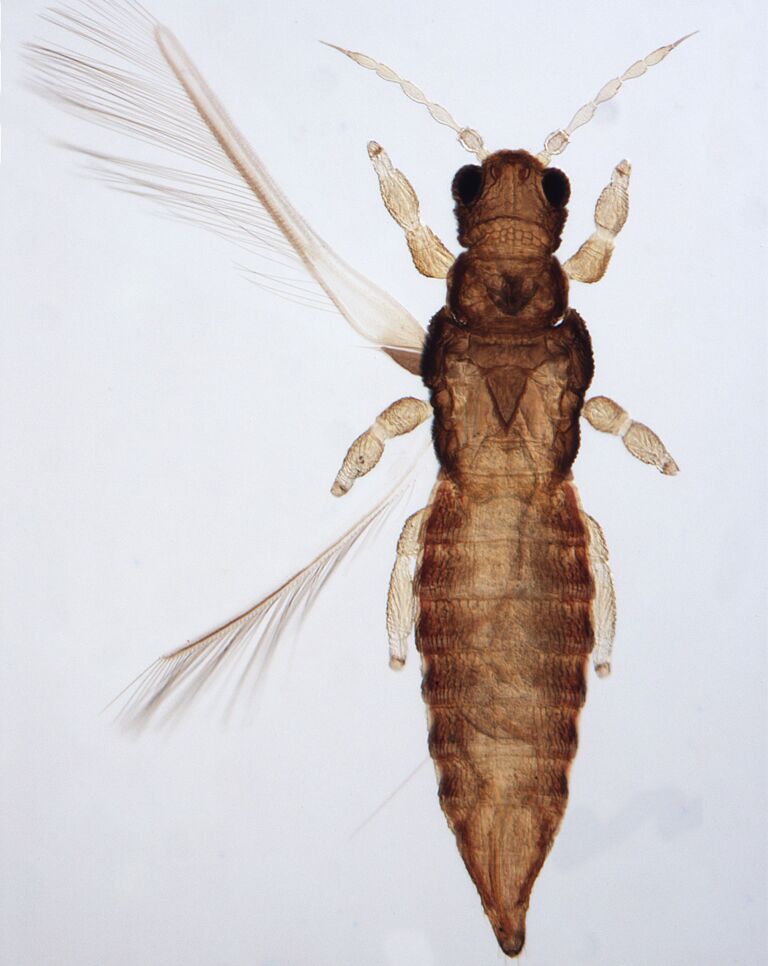
Female of *Rhipiphorothripscruentacus* Hood, 1919.

Female macropterous; body dark brown (Fig. 11), antennae and legs largely yellow, fore wing pale with yellow veins; Head with complex irregular sculpture, cheeks sharply incut behind eyes and constricted to basal neck; antennae 8-segmented, segments III & IV with simple sensorium; VIII more than twice as long as VII. Pronotum without long setae; mesonotum with complete longitudinal division, metanotum with well developed reticulate triangle with a pair of minute setae near posterior and a pair of campaniform sensilla. Fore wing rounded at apex with 2 slender cilia; Abdominal tergites III–VIII with grooved medially, with 1 pair of strong median setae; tergites strongly sculptured laterally. Male similar to the female, but smaller with small circular pore plate on anterior margin of sternites III–VII.

#### Rhipiphorothrips pulchellus

Morgan, 1913

58FDAE71-7923-54D5-9125-8B00EADB5311

https://thrips.info/wiki/Rhipiphorothrips_pulchellus

Rhipiphorothrips pulchellus Morgan, 1913: 17
Retithrips bicolor Bagnall, 1913: 290.

##### Materials

- **Type status:** Other material. **Occurrence:** recordedBy: X.Y.L; individualID: 2018-xi-17; individualCount: 10; sex: 4 males, 6 females; lifeStage: adults; occurrenceID: YAU5082020Tt26; **Taxon:** scientificNameAuthorship: *Rhipiphorothripspulchellus* Morgan; **Location:** country: China; stateProvince: Yunnan; municipality: Xishuangbanna; locality: Mengla (Tropical Botanical Garden); decimalLatitude: 21.930611; decimalLongitude: 101.258129; **Identification:** identifiedBy: Xie Yanlan; dateIdentified: 2018; identificationReferences: (ThripsWiki 2020); **Event:** samplingProtocol: sweeping and shaking; eventDate: 17/11/2018; **Record Level:** collectionID: thrips; institutionCode: YAU5082020; collectionCode: terebrantia; basisOfRecord: preserved specimen

##### Ecological interactions

###### Feeds on

leaves and collected from grape and a wide range of fruits.

##### Distribution

Described from Sri Lanka and the Philippines. Recorded from India, Indonesia and China (Xishuangbanna).

##### Diagnosis

**Figure 12.**
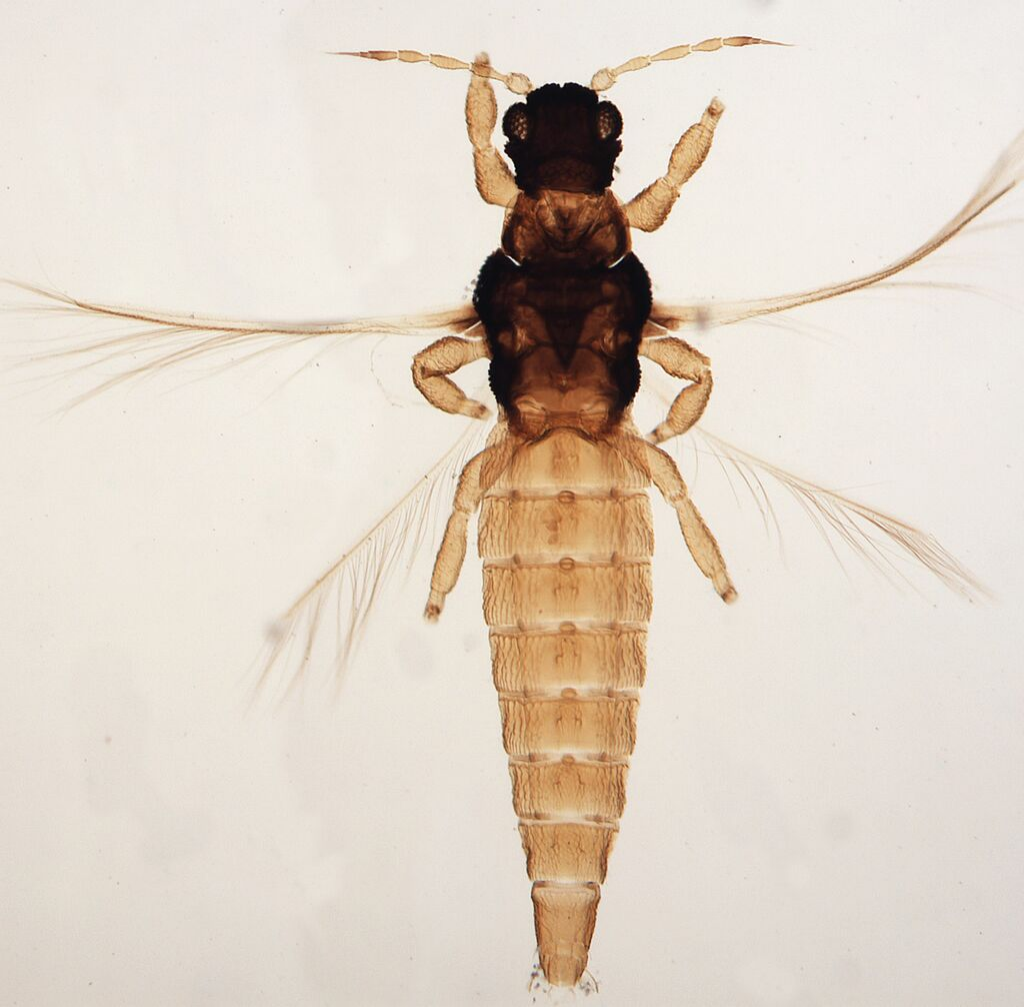
Male of *Rhipiphorothripspulchellus* Morgan, 1913.

*R.pulchellus* differs from other species of the same genus by the yellow pronotum and abdominal segments (Fig. 12) and the male does not have a tubercle laterally on abdominal segment IV.

#### Selenothrips rubrocinctus

(Giard, 1901)

213C2216-8B25-56C1-A448-44CDD46966F4

https://thrips.info/wiki/Selenothrips_rubrocinctus

Physopus rubrocincta Giard, 1901: 264 | Heliothrips (Selenothrips) decolor Karny, 1911: 179 | Heliothrips (Selenothrips) mendax Schmutz, 1913: 994
Brachyurothrips indicus Bagnall, 1926: 98.

##### Materials

- **Type status:** Other material. **Occurrence:** recordedBy: L.Y.J; individualID: 2017-x-24; individualCount: 4; sex: females; lifeStage: adults; occurrenceID: YAU5082020Tt27; **Taxon:** scientificNameAuthorship: *Selenothripsrubrocinctus* (Giard); **Location:** country: China; stateProvince: Yunnan; municipality: Xishuangbanna; locality: Mengla (Menglun); decimalLatitude: 21.937451; decimalLongitude: 101.257842; **Identification:** identifiedBy: Xie Yanlan; dateIdentified: 2018; identificationReferences: (ThripsWiki 2020); **Event:** samplingProtocol: sweeping and shaking; eventDate: 24/10/2017; **Record Level:** collectionID: thrips; institutionCode: YAU5082020; collectionCode: terebrantia; basisOfRecord: preserved specimen

##### Ecological interactions

###### Feeds on

leaves and collected from Rosaceae.

##### Distribution

Described from Sri Lanka and recorded in West Papua, Indonesia, India, China and widely distributed from Subtropical zone.

##### Diagnosis

**Figure 13.**
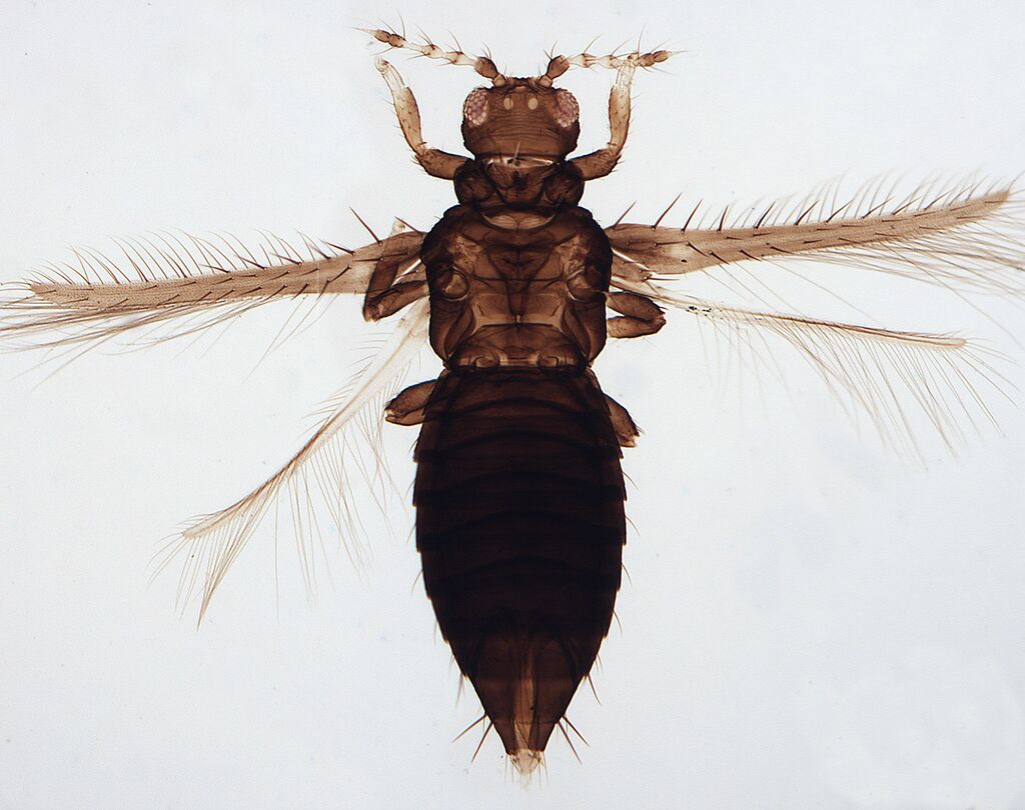
Female of *Selenothripsrubrocinctus* Giard, 1901.

Both sexes macropterous; female about 1.2 mm in length (Fig. 13); dark brown to black body underlain by red pigment chiefly in the first three abdominal segments; the anal segments retain a reddish-black colour and the wings are dark, male similar to female, but smaller.

#### Zaniothrips ricini

Bhatti, 1967

C4E6ADB9-E461-5ED4-9180-F2C4D8D89D8B

https://thrips.info/wiki/Zaniothrips_ricini

##### Materials

- **Type status:** Other material. **Occurrence:** recordedBy: E.N & X.Y.L; individualID: 2019-v-12; individualCount: 3; sex: females; lifeStage: adults; occurrenceID: YAU5082020Tt28; **Taxon:** scientificNameAuthorship: *Zaniothripsricini* Bhatti; **Location:** country: China; stateProvince: Yunnan; municipality: Xishuangbanna; locality: Mengla (Menglun); decimalLatitude: 21.932623; decimalLongitude: 101.271352; **Identification:** identifiedBy: Xie Yanlan; dateIdentified: 2019; identificationReferences: (ThripsWiki 2020); **Event:** samplingProtocol: sweeping and shaking; eventDate: 12/05/2019; **Record Level:** collectionID: thrips; institutionCode: YAU5082020; collectionCode: terebrantia; basisOfRecord: preserved specimen

##### Ecological interactions

###### Feeds on

leaves and collected from Moraceae.

##### Distribution

Described in India and recorded from China (Xishuangbanna).

##### Diagnosis

**Figure 14.**
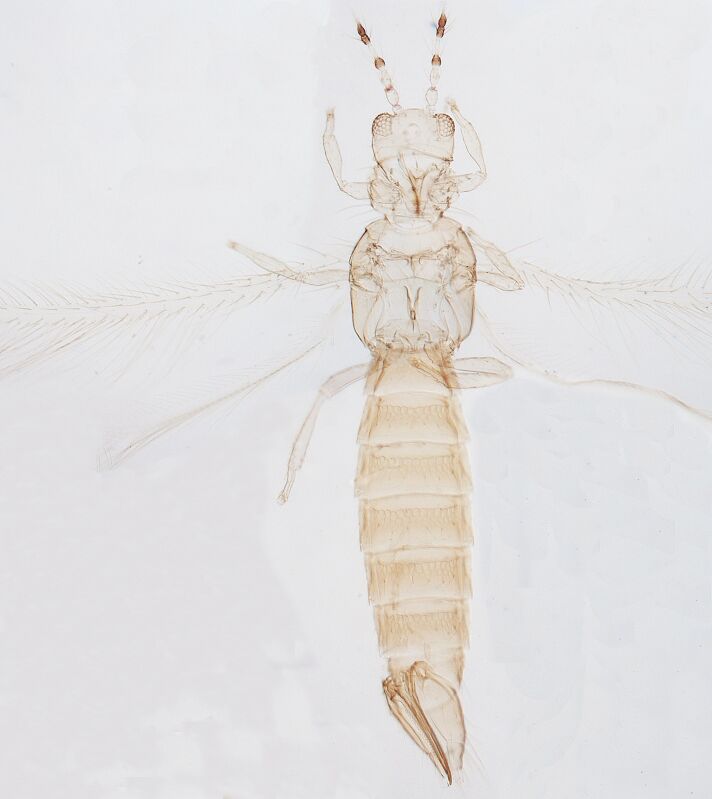
Female of *Zaniothripsricini* Bhatti, 1978.

Female macropterous, body brown to paler (Fig. 14); head wider than long, weakly reticulated with broad reticulate posterior collar; ocellar setae I present, II longer than III; three pairs of well-developed postocular setae; maxillary palps 2-segmented; antennae 8- segmented, segment I without paired dorso-apical setae; III with sense cone forked, IV with one forked and one simple sense cones. Pronotal with or without weak sculptures; metanotum weakly reticulate, median setae close to posterior margin, campaniform sensilla present; fore wing with anterior margin fringe cilia shorter than costa setae.

#### Dendrothripinae

EC5B338C-3A3B-5C0E-BD0C-CA117818E1DC

#### Dendrothrips minowai

Priesner, 1935

3318694F-6512-5B4C-A1B5-31F50DD9E600

https://thrips.info/wiki/Dendrothrips_minowai

Dendrothrips schimae Kudo, 1989: 42. Synonymised by Wang et al. (2019).

##### Materials

- **Type status:** Other material. **Occurrence:** recordedBy: J.M.M; individualID: 2009-XII-5|2009-XII-2|2009-IV-6; individualCount: 100; sex: 12 males, 88 females; lifeStage: adults; occurrenceID: YAU5082020Tt29; **Taxon:** scientificNameAuthorship: *Dendrothripsminowai* Priesner; **Location:** country: China; stateProvince: Yunnan; municipality: Xishuangbanna; locality: Menghai (Protected area), Mengla (Menglun); decimalLatitude: 21.923369; decimalLongitude: 101.276527; **Identification:** identifiedBy: Li Yajin; dateIdentified: 2018; identificationReferences: (ThripsWiki 2020); **Event:** samplingProtocol: sweeping and shaking; eventDate: 06/04/2009, 02/12/2009, 05/12/2009; startDayOfYear: |2009-XII-2|2009-IV-6; **Record Level:** collectionID: thrips; institutionCode: YAU5082020; collectionCode: terebrantia; basisOfRecord: preserved specimen

##### Ecological interactions

###### Feeds on

leaves, collected from tea leaves, Poaceae and *Spatholobussuberectus* Dunn.

##### Distribution

Described from Japan. Recorded from Nepal and China (Xishuangbanna).

#### Dendrothrips strasseni

Bhatti, 1971

388518BE-D74A-545D-BEFB-0F788442FE8C

https://thrips.info/wiki/Dendrothrips_strasseni

##### Materials

- **Type status:** Other material. **Occurrence:** recordedBy: S.S.Q; individualID: 2009-III-15; individualCount: 9; sex: females; lifeStage: adults; occurrenceID: YAU5082020Tt30; **Taxon:** scientificNameAuthorship: *Dendrothripsstrasseni* Bhatti; **Location:** country: China; stateProvince: Yunnan; municipality: Xishuangbanna; locality: Jinghong (Nabanhe Protected Area); decimalLatitude: 21.915456; decimalLongitude: 101.275664; **Identification:** identifiedBy: Li Yajin; dateIdentified: 2018; identificationReferences: (ThripsWiki 2020); **Event:** samplingProtocol: sweeping and shaking; eventDate: 03-15-09; **Record Level:** collectionID: thrips; institutionCode: YAU5082020; collectionCode: terebrantia; basisOfRecord: preserved specimen

##### Ecological interactions

###### Feeds on

leaves and collected from *Populustremula* (‎Salicaceae).

##### Distribution

Described from India and recorded from China (Xishuangbanna).

#### Filicopsothrips pulcher

Li, Yuan & Zhang, 2020

E56D565B-E8C1-56B9-A0C7-FF4776F3315D

https://thrips.info/wiki/Filicopsothrips_pulcher

##### Materials

- **Type status:** Other material. **Occurrence:** recordedBy: L.H & E.N; individualID: 2018-v-21; individualCount: 1; sex: female; lifeStage: adults; occurrenceID: YAU5082020Tt31; **Taxon:** scientificNameAuthorship: *Filicopsothripspulcher* Li, Yuan & Zhang; **Location:** country: China; stateProvince: Yunnan; municipality: Xishuangbanna; locality: Mengla (Menglun); decimalLatitude: 21.911298; decimalLongitude: 101.285581; **Identification:** identifiedBy: Li Yajin; dateIdentified: 2018; identificationReferences: (ThripsWiki 2020); **Event:** samplingProtocol: sweeping and shaking; eventDate: 21/05/2018; **Record Level:** collectionID: thrips; institutionCode: YAU5082020; collectionCode: terebrantia; basisOfRecord: preserved specimen

##### Ecological interactions

###### Feeds on

leaves and collected from grasses (Poaceae).

##### Distribution

Described from China (Xishuangbanna) (Li et al. 2020).

##### Diagnosis

**Figure 15.**
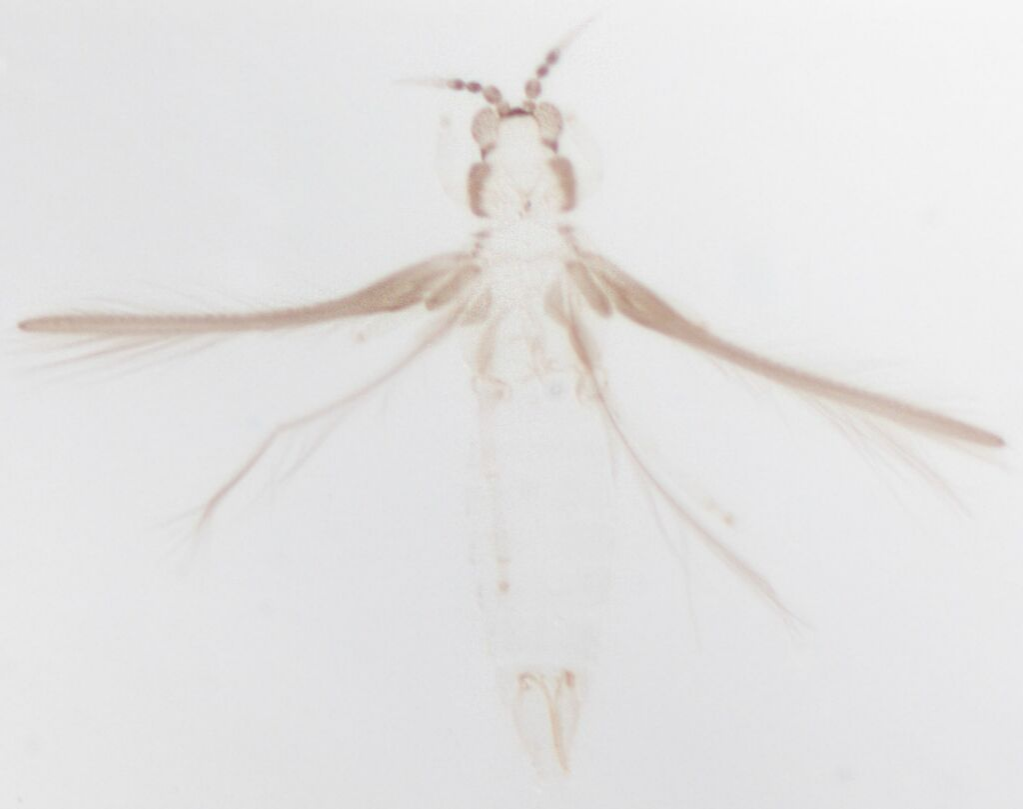
Female of *Filicopsothripspulcher* Li, Yuan & Zhang, 2020.

Female fully winged, body bicoloured (Fig. 15); head brown laterally and at anterior, white medially; antennal segments I–IV brown, V–IX paler, VI with sense cone on basal half; pronotum shaded on lateral margins, with two longitudinal brown markings laterally; abdominal tergites paler; fore wing brown and all legs paler.

#### Pseudodendrothrips bhattii

Kudo, 1984

8D5C5E1B-D164-5AC9-8B3E-FEA1A04FFBAF

https://thrips.info/wiki/Pseudodendrothrips_bhattii

##### Materials

- **Type status:** Other material. **Occurrence:** recordedBy: L.Y.J; individualID: 2017-X-24; individualCount: 2; sex: females; lifeStage: adults; occurrenceID: YAU5082020Tt32; **Taxon:** scientificNameAuthorship: *Pseudodendrothripsbhattii* Kudo; **Location:** country: China; stateProvince: Yunnan; municipality: Xishuangbanna; locality: Mengla (Tropical Botanical Garden); decimalLatitude: 21.914786; decimalLongitude: 101.291474; **Identification:** identifiedBy: Li Yajin; dateIdentified: 2018; identificationReferences: (ThripsWiki 2020); **Event:** samplingProtocol: sweeping and shaking; eventDate: 24/10/2017; **Record Level:** collectionID: thrips; institutionCode: YAU5082020; collectionCode: terebrantia; basisOfRecord: preserved specimen

##### Ecological interactions

###### Feeds on

leaves and collected from *Senegaliapennata* (Fabaceae).

##### Distribution

Described from Japan and recorded from China (Xishuangbanna).

#### Pseudodendrothrips mori

(Niwa, 1908)

C037CEE3-7772-5EEA-9581-589C22F348DF

https://thrips.info/wiki/Pseudodendrothrips_mori

Belothrips mori Niwa, 1908: 180
Graphidothrips stuardoi Moulton, 1930: 273.

##### Materials

- **Type status:** Other material. **Occurrence:** recordedBy: S.S.Q; individualID: 2010-IV-29|2017-X-24; individualCount: 11; sex: 4 males, 7 females; lifeStage: adults; occurrenceID: YAU5082020Tt33; **Taxon:** scientificNameAuthorship: *Pseudodendrothripsmori* (Niwa); **Location:** country: China; stateProvince: Yunnan; municipality: Xishuangbanna; locality: Menghai (Protected Area); decimalLatitude: 21.92404; decimalLongitude: 101.28515; **Identification:** identifiedBy: Li Yajin; dateIdentified: 2018; identificationReferences: (ThripsWiki 2020); **Event:** samplingProtocol: sweeping and shaking; eventDate: 29/04/2010, 24/10/2017; **Record Level:** collectionID: thrips; institutionCode: YAU5082020; collectionCode: terebrantia; basisOfRecord: preserved specimen

##### Ecological interactions

###### Feeds on

leaves and collected from *Senegaliapennata* (Fabaceae).

##### Distribution

Described from Japan. Recorded from China, Korea, Taiwan, Australia, Italy, Chile and USA (California, Georgia, Maryland, Illinois).

#### Pseudodendrothrips pueraria

Zhang & Tong, 1990

E835FBF7-9C16-5D68-AFD4-11C5F361B36D

https://thrips.info/wiki/Pseudodendrothrips_puerariae

##### Materials

- **Type status:** Other material. **Occurrence:** recordedBy: Z.W.Q; individualID: 1987-IV-7; individualCount: 1; sex: female; lifeStage: adults; occurrenceID: YAU5082020Tt34; **Taxon:** scientificNameAuthorship: *Pseudodendrothripspueraria* Zhang & Tong; **Location:** country: China; stateProvince: Yunnan; municipality: Xishuangbanna; locality: Mengla (Menglun); decimalLatitude: 21.926856; decimalLongitude: 101.317346; **Identification:** identifiedBy: Li Yajin; dateIdentified: 2018; identificationReferences: (ThripsWiki 2020); **Event:** samplingProtocol: sweeping and shaking; eventDate: 07/04/1987; **Record Level:** collectionID: thrips; institutionCode: YAU5082020; collectionCode: terebrantia; basisOfRecord: preserved specimen

##### Ecological interactions

###### Feeds on

leaves and collected from *Puerariamontana* (Fabaceae).

##### Distribution

Described from China (Xishuangbanna).

#### Sericothripinae

3F69225E-BCE6-53EC-9367-561066EA6DDA

#### Hydatothrips aureus

Bhatti, 1973

15A68917-0262-5524-AD78-02AA0DCB67AB

https://thrips.info/wiki/Hydatothrips_aureus

##### Materials

- **Type status:** Other material. **Occurrence:** recordedBy: L.H; individualID: 2017-X-24; individualCount: 5; sex: 2 males, 3 females; lifeStage: adults; occurrenceID: YAU5082020Tt35; **Taxon:** scientificNameAuthorship: *Hydatothripsaureus* Bhatti; **Location:** country: China; stateProvince: Yunnan; municipality: Xishuangbanna; locality: Mengla (Tropical Botanical Garden); decimalLatitude: 21.927891; decimalLongitude: 101.311502; **Identification:** identifiedBy: Li Yajin; dateIdentified: 2018; identificationReferences: (ThripsWiki 2020); **Event:** samplingProtocol: sweeping and shaking; eventDate: 24/10/2017; **Record Level:** collectionID: thrips; institutionCode: YAU5082020; collectionCode: terebrantia; basisOfRecord: preserved specimen

##### Ecological interactions

###### Feeds on

flowers and collected from *Calleryadielsiana* (Papilionaceae).

##### Distribution

Described from India and worldwide distributed.

#### Hydatothrips dorax

Bhatti, 1973

00A8A5A0-E758-57A7-AF30-9BFCE40D3EE6

https://thrips.info/wiki/Hydatothrips_dorax

##### Materials

- **Type status:** Other material. **Occurrence:** recordedBy: L.Y.J; individualID: 2017-II-10; individualCount: 4; sex: 2 males, 2 females; lifeStage: adults; occurrenceID: YAU5082020Tt36; **Taxon:** scientificNameAuthorship: *Hydatothripsdorax* Bhatti; **Location:** country: China; stateProvince: Yunnan; municipality: Xishuangbanna; locality: Mengla (Menglun); decimalLatitude: 21.959907; decimalLongitude: 100.463502; **Identification:** identifiedBy: Li Yajin; dateIdentified: 2018; identificationReferences: (ThripsWiki 2020); **Event:** samplingProtocol: sweeping and shaking; eventDate: 10/02/2017; **Record Level:** collectionID: thrips; institutionCode: YAU5082020; collectionCode: terebrantia; basisOfRecord: preserved specimen

##### Ecological interactions

###### Feeds on

flowers and collected from grasses (Poaceae).

##### Distribution

Described from India and recorded from China (Xishuangbanna).

#### Hydatothrips flavidus

Wang, 2007

032BE16B-EC03-5FD9-8AFD-9C842FE8914A

https://thrips.info/wiki/Hydatothrips_flavidus

##### Materials

- **Type status:** Other material. **Occurrence:** recordedBy: L.Y.J; individualID: 2017-III-11; individualCount: 2; sex: females; lifeStage: adults; occurrenceID: YAU5082020Tt37; **Taxon:** scientificNameAuthorship: *Hydatothripsflavidus* Wang; **Location:** country: China; stateProvince: Yunnan; municipality: Xishuangbanna; locality: Mengla (Tropical Botanical Garden); decimalLatitude: 21.959857; decimalLongitude: 100.46016; **Identification:** identifiedBy: Li Yajin; dateIdentified: 2018; identificationReferences: (ThripsWiki 2020); **Event:** samplingProtocol: sweeping and shaking; eventDate: 11/03/2017; **Record Level:** collectionID: thrips; institutionCode: YAU5082020; collectionCode: terebrantia; basisOfRecord: preserved specimen

##### Ecological interactions

###### Feeds on

flowers and collected from *Litchichinensis* (Sapindaceae).

##### Distribution

Described from Taiwan and distributed from China (Yunnan Province).

#### Neohydatothrips plynopygus

(Karny, 1925)

3BEE6EED-A884-5525-BBD6-3872FEE36533

https://thrips.info/wiki/Neohydatothrips_plynopygus

Anaphothrips plynopygus Karny, 1925: 29
Zonothrips luridus Ananthakrishnan, 1968: 115.

##### Materials

- **Type status:** Other material. **Occurrence:** recordedBy: L.Y.J; individualID: 2017-II-11; individualCount: 8; sex: 2 males, 6 females; lifeStage: adults; occurrenceID: YAU5082020Tt38; **Taxon:** scientificNameAuthorship: *Neohydatothripsplynopygus* (Karny); **Location:** country: China; stateProvince: Yunnan; municipality: Xishuangbanna; locality: Jinghong (Naban He Protected Area); decimalLatitude: 21.963879; decimalLongitude: 100.457142; **Identification:** identifiedBy: Li Yajin; dateIdentified: 2018; identificationReferences: (ThripsWiki 2020); **Event:** samplingProtocol: sweeping and shaking; eventDate: 11/02/2017; **Record Level:** collectionID: thrips; institutionCode: YAU5082020; collectionCode: terebrantia; basisOfRecord: preserved specimen

##### Ecological interactions

###### Feeds on

flowers and leaves, collected from raspberry and congo grass.

##### Distribution

Described from Indonesia and India. Recorded from Singapore, Taiwan, China (Yunnan Province) and Australia.

#### Neohydatothrips Samayunkur

(Kudo, 1995)

BBA8D708-8975-5D70-8E8D-9DFDDAC78A2A

https://thrips.info/wiki/Neohydatothrips_samayunkur

Hydatothrips (Neohydatothrips) samayunkur Kudo, 1995: 169.

##### Materials

- **Type status:** Other material. **Occurrence:** recordedBy: X.Y.H; individualID: 2011-X-2; individualCount: 12; sex: females; lifeStage: adults; occurrenceID: YAU5082020Tt39; **Taxon:** scientificNameAuthorship: *NeohydatothripsSamayunkur* (Kudo); **Location:** country: China; stateProvince: Yunnan; municipality: Xishuangbanna; locality: Mengla (Tropical Botanical Garden), Jinghong (Nabanhe Protected Area); decimalLatitude: 21.954225; decimalLongitude: 100.44859; **Identification:** identifiedBy: Li Yajin; dateIdentified: 2018; identificationReferences: (ThripsWiki 2020); **Event:** samplingProtocol: sweeping and shaking; eventDate: 02/10/2011; **Record Level:** collectionID: thrips; institutionCode: YAU5082020; collectionCode: terebrantia; basisOfRecord: preserved specimen

##### Ecological interactions

###### Feeds on

flowers and leaves, collected from *Tageteserecta* (Asteraceae).

##### Distribution

Described from Japan. Recorded in Hawaii, Florida, Australia, Kenya, South Africa, Mauritius, New Zealand, Mexico, Taiwan and China (Yunnan Province).

#### Thripinae

7A43C3BB-6033-545E-AF33-329A101321E0

#### Amomothrips associatus

(Priesner, 1938)

3F6E5DF4-0C1F-50DC-8B80-91D8B5379CFF

https://thrips.info/wiki/Amomothrips_associatus

Taeniothrips associatus Priesner, 1938: 483.

##### Materials

- **Type status:** Other material. **Occurrence:** recordedBy: X.Y.H; individualID: 2011-IX-30; individualCount: 6; sex: 2 males, 4 females; lifeStage: adults; occurrenceID: YAU5082020Tt40; **Taxon:** scientificNameAuthorship: *Amomothripsassociatus* (Priesner); **Location:** country: China; stateProvince: Yunnan; municipality: Xishuangbanna; locality: Mengla (Tropical Botanical Garden); decimalLatitude: 21.962539; decimalLongitude: 100.446218; **Identification:** identifiedBy: Li Yajin; dateIdentified: 2018; identificationReferences: (ThripsWiki 2020); **Event:** samplingProtocol: sweeping and shaking; eventDate: 30/09/2011; **Record Level:** collectionID: thrips; institutionCode: YAU5082020; collectionCode: terebrantia; basisOfRecord: preserved specimen

##### Ecological interactions

###### Feeds on

flowers and collected from *Alpiniavittata* (Zingiberaceae).

##### Distribution

Described from Malaysia and China (Yunnan Province).

##### Diagnosis

**Figure 16.**
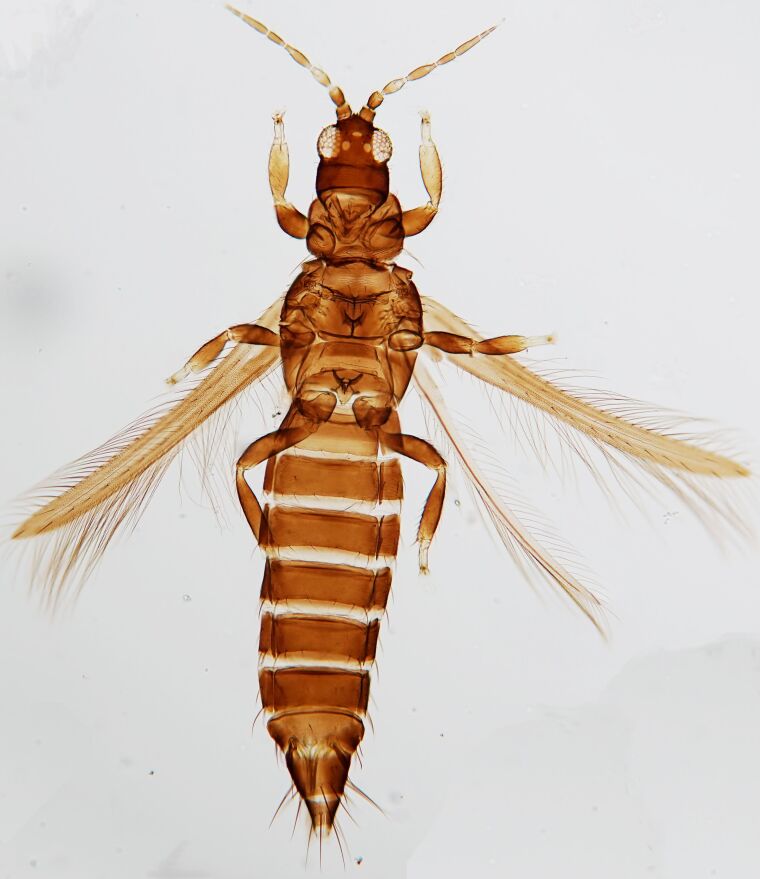
Female of *Amomothripsassociatus* (Bhatti, 1978).

Female fully-winged; body dark brown (Fig. 16), all legs brown, except yellow apicals of tibia and tarsi; antenna segments I–II dark brown, III brown with apex light brown, segments IV–VIII brown; fore wing brown, head longer than wider with sculpture, close striates behind eyes, cheeks slightly constricted; antenna 8-segmented, segment I without dorso-apical setae, III & IV with long and forked sensoria, III with pedicel; pronotum wider than long, sculptured with close transverse striations with four pairs of posteromaginal setae. Male similar to female, but smaller.

#### Anaphothrips floralis

Karny, 1922

B1597AD9-85C4-5AEF-A077-FA198843B35E

https://thrips.info/wiki/Anaphothrips_floralis

##### Materials

- **Type status:** Other material. **Occurrence:** recordedBy: S.S.Q; individualID: 2009-IV-17; individualCount: 1; sex: female; lifeStage: adults; occurrenceID: YAU5082020Tt41; **Taxon:** scientificNameAuthorship: *Anaphothripsfloralis* Karny; **Location:** country: China; stateProvince: Yunnan; municipality: Xishuangbanna; locality: Mengla (Tropical Botanical Garden); decimalLatitude: 21.963879; decimalLongitude: 100.359622; **Identification:** identifiedBy: Li Yajin; dateIdentified: 2018; identificationReferences: (ThripsWiki 2020); **Event:** samplingProtocol: sweeping and shaking; eventDate: 17/04/2009; **Record Level:** collectionID: thrips; institutionCode: YAU5082020; collectionCode: terebrantia; basisOfRecord: preserved specimen

##### Ecological interactions

###### Feeds on

flowers and collected from dandelion and allium (Fabaceae and Liliaceae).

##### Distribution

Described from Vietnam and recorded from China (Xishuangbanna).

#### Anaphothrips incertus

(Girault, 1929)

E5D19DCD-E896-5EA3-8060-A776DF48A64B

https://thrips.info/wiki/Anaphothrips_incertus

Limothrips incertus Girault, 1929: 3.

##### Materials

- **Type status:** Other material. **Occurrence:** recordedBy: L.Y.J; individualID: 2018-V-28; individualCount: 1; sex: female; lifeStage: adults; occurrenceID: YAU5082020Tt42; **Taxon:** scientificNameAuthorship: *Anaphothripsincertus* (Girault); **Location:** country: China; stateProvince: Yunnan; municipality: Xishuangbanna; locality: Mengla (Tropical Botanical Garden); decimalLatitude: 22.045911; decimalLongitude: 100.479779; **Identification:** identifiedBy: Li Yajin; dateIdentified: 2018; identificationReferences: (ThripsWiki 2020); **Event:** samplingProtocol: sweeping and shaking; eventDate: 28/05/2018; **Record Level:** collectionID: thrips; institutionCode: YAU5082020; collectionCode: terebrantia; basisOfRecord: preserved specimen

##### Ecological interactions

###### Feeds on

flowers and collected from *Kyllingabrevifolia* (Poaceae).

##### Distribution

Described from Queensland. Recorded from Australia and China (Xishuangbanna).

#### Anascirtothrips discordiae

Chen & Lu, 1994

320D3225-D42E-55A2-B513-A4778C5E18DD

https://thrips.info/wiki/Anascirtothrips_discordiae

##### Materials

- **Type status:** Other material. **Occurrence:** recordedBy: X.Y.L; individualID: 2017-VI-24; individualCount: 1; sex: female; lifeStage: adults; occurrenceID: YAU5082020Tt43; **Taxon:** scientificNameAuthorship: *Anascirtothripsdiscordiae* Chen & Lu; **Location:** country: China; stateProvince: Yunnan; municipality: Xishuangbanna; locality: Mengla (Menglun); decimalLatitude: 21.917631; decimalLongitude: 100.407825; **Identification:** identifiedBy: Li Yajin; dateIdentified: 2018; identificationReferences: (ThripsWiki 2020); **Event:** samplingProtocol: sweeping and shaking; eventDate: 24/06/2017; **Record Level:** collectionID: thrips; institutionCode: YAU5082020; collectionCode: terebrantia; basisOfRecord: preserved specimen

##### Ecological interactions

###### Feeds on

leaves, collected from ficus, *Vitisamurensis*.

##### Distribution

Described from Taiwan and recorded from China (Xishuangbanna).

#### Arorathrips mexicanus

(Crawford DL, 1909)

567DA6B1-F7B7-5236-872E-6F35CB06C6CB

https://thrips.info/wiki/Arorathrips_mexicanus

Chirothrips mexicana Crawford DL, 1909: 114
Chirothrips floridensis Watson, 1920: 22
Chirothrips catchingsi Watson, 1924: 76. Chirothrips saltensis Tapia, 1952: 109

##### Materials

- **Type status:** Other material. **Occurrence:** recordedBy: L.Y.J; individualID: 2017-X-22; individualCount: 2; sex: females; lifeStage: adults; occurrenceID: YAU5082020Tt44; **Taxon:** scientificNameAuthorship: *Arorathripsmexicanus* (Crawford DL); **Location:** country: China; stateProvince: Yunnan; municipality: Xishuangbanna; locality: Mengla (Tropical Forest Park), Jinghong (Nabanhe Protected Area, Ye Xianggu); decimalLatitude: 21.961529; decimalLongitude: 100.462693; **Identification:** identifiedBy: Li Yajin; dateIdentified: 2018; identificationReferences: (ThripsWiki 2020); **Event:** samplingProtocol: sweeping and shaking; eventDate: 22/10/2017; **Record Level:** collectionID: thrips; institutionCode: YAU5082020; collectionCode: terebrantia; basisOfRecord: preserved specimen

##### Ecological interactions

###### Feeds on

Flower and collected from grasses (Poaceae).

##### Distribution

Described from Mexico. Recorded from New Orleans, Louisiana, Florida, Argentina and China.

#### Aroidothrips longistylus

Ananthakrishnan, 1960

36F995E6-2BB6-579B-846E-0DFCD25A2FD8

https://thrips.info/wiki/Aroidothrips_longistylus

##### Materials

- **Type status:** Other material. **Occurrence:** recordedBy: L.Y.J, X.Y.L & Z.H.R; individualID: 2017-III-11|2017-III-11|2017-III-25|2017-X-22|2018-V-27|2018-V-27; individualCount: 8; sex: 1 males, 7 females; lifeStage: adults; occurrenceID: YAU5082020Tt45; **Taxon:** scientificNameAuthorship: *Aroidothripslongistylus* Ananthakrishnan; **Location:** country: China; stateProvince: Yunnan; municipality: Xishuangbanna; locality: Mengla (Tropical Botanical Garden); decimalLatitude: 21.96147; decimalLongitude: 100.462909; **Identification:** identifiedBy: Li Yajin; dateIdentified: 2018; identificationReferences: (ThripsWiki 2020); **Event:** samplingProtocol: sweeping and shaking; eventDate: 11/03/2017, 22/10/2017, 25/03/2017, 27/05/2018; **Record Level:** collectionID: thrips; institutionCode: YAU5082020; collectionCode: terebrantia; basisOfRecord: preserved specimen

##### Ecological interactions

###### Feeds on

Further researches are needed to identify the species feeding habits. Collected from Chloranthaceae, *Ficusvasculosa*, *Pittosporopsiskerrii* (Alseuosmiaceae).

##### Distribution

Described from India and recorded from China (Xishuangbanna).

#### Ayyaria chaetophora

Karny, 1926

3816B2B0-0988-539F-A408-D8FE85619093

https://thrips.info/wiki/Ayyaria_chaetophora

Bussothrips claratibia Moulton, 1935: 475
Parafrankliniella fasciatus Kurosawa, 1937: 271
Parafrankliniella subfasciatus Kurosawa, 1968: 24.

##### Materials

- **Type status:** Other material. **Occurrence:** recordedBy: L.Y.J, S.S.Q, & E.N; individualID: 2019-II-16|2017-III-11; individualCount: 2; sex: females; lifeStage: adults; occurrenceID: YAU5082020Tt46; **Taxon:** scientificNameAuthorship: *Ayyariachaetophora* Karny; **Location:** country: China; stateProvince: Yunnan; municipality: Xishuangbanna; locality: Mengla (Tropical Botanical Garden); decimalLatitude: 21.959819; decimalLongitude: 100.460016; **Identification:** identifiedBy: Li Yajin; dateIdentified: 2018; identificationReferences: (ThripsWiki 2020); **Event:** samplingProtocol: sweeping and shaking; eventDate: 11/03/2017, 16/02/2019; **Record Level:** collectionID: thrips; institutionCode: YAU5082020; collectionCode: terebrantia; basisOfRecord: preserved specimen

##### Ecological interactions

###### Feeds on

leaves, collected from *Litchichinensis* and mango.

##### Distribution

Described from India. Recorded from Japan and China (Xishuangbanna).

#### Bathrips jasminae

Ananthakrishnan, 1968

4B388818-4F44-51BC-B7DE-8FD251605C10

https://thrips.info/wiki/Bathrips_jasminae

##### Materials

- **Type status:** Other material. **Occurrence:** recordedBy: L.Y.J; individualID: 2017-III-11; individualCount: 7; sex: 1 males, 6 females; lifeStage: adults; occurrenceID: YAU5082020Tt47; **Taxon:** scientificNameAuthorship: *Bathripsjasminae* Ananthakrishnan; **Location:** country: China; stateProvince: Yunnan; municipality: Xishuangbanna; locality: Mengla (Tropical Botanical Garden), Jinghong (Nabanhe Protected area + Botanical Garden); decimalLatitude: 21.959811; decimalLongitude: 100.463996; **Identification:** identifiedBy: Li Yajin; dateIdentified: 2018; identificationReferences: (ThripsWiki 2020); **Event:** samplingProtocol: sweeping and shaking; eventDate: 11/03/2017; **Record Level:** collectionID: thrips; institutionCode: YAU5082020; collectionCode: terebrantia; basisOfRecord: preserved specimen

##### Ecological interactions

###### Feeds on

leaves, collected from golden privet, tea, jasmine, *Osmanthusfragrans*.

##### Distribution

Described from India and recorded from southern China.

#### Bathrips melanicornis

(Shumsher, 1946)

01BB7DCF-E666-59D8-A973-62A749A2D332

https://thrips.info/wiki/Bathrips_melanicornis

Taeniothrips melanicornis Shumsher, 1946: 179
Taeniothrips ipomoeae Zhang, 1981: 324. Synonymised by Mirab-Balou et al. (2012).

##### Materials

- **Type status:** Other material. **Occurrence:** recordedBy: L.Y.J; individualID: 2017-III-11; individualCount: 4; sex: 2 males, 2 females; lifeStage: adults; occurrenceID: YAU5082020Tt48; **Taxon:** scientificNameAuthorship: *Bathripsmelanicornis* (Shumsher); **Location:** country: China; stateProvince: Yunnan; municipality: Xishuangbanna; locality: Mengla (Tropical Botanical Garden); decimalLatitude: 21.957163; decimalLongitude: 100.46193; **Identification:** identifiedBy: Li Yajin; dateIdentified: 2018; identificationReferences: (ThripsWiki 2020); **Event:** samplingProtocol: sweeping and shaking; eventDate: 11/03/2017; **Record Level:** collectionID: thrips; institutionCode: YAU5082020; collectionCode: terebrantia; basisOfRecord: preserved specimen

##### Ecological interactions

###### Feeds on

leaves, collected from sweet potato, *Lantanacamara*, mango tree, beans and eggplant.

##### Distribution

Described from Myanmar, China (Guangdong & Yunnan Province). Recorded from India, Malaysia, Indonesia, Thailand, East Timor, Australia and Iran.

#### Bolacothrips graminis

Priesner, 1930

8C2882B2-0DCF-53F1-90F3-8E102D0D2DA4

https://thrips.info/wiki/Bolacothrips_graminis

##### Materials

- **Type status:** Other material. **Occurrence:** recordedBy: L.Y.J; individualID: 2011-XII-27; individualCount: 4; sex: 2 males, 2 females; lifeStage: adults; occurrenceID: YAU5082020Tt49; **Taxon:** scientificNameAuthorship: *Bolacothripsgraminis* Priesner; **Location:** country: China; stateProvince: Yunnan; municipality: Xishuangbanna; locality: Mengla (Tropical Botanical Garden); decimalLatitude: 21.957615; decimalLongitude: 100.462244; **Identification:** identifiedBy: Li Yajin; dateIdentified: 2018; identificationReferences: (ThripsWiki 2020); **Event:** samplingProtocol: sweeping and shaking; eventDate: 27/11/2011; **Record Level:** collectionID: thrips; institutionCode: YAU5082020; collectionCode: terebrantia; basisOfRecord: preserved specimen

##### Ecological interactions

###### Feeds on

leaves and collected from Poaceae.

##### Distribution

Described from Egypt and recorded from China (Xishuangbanna).

#### Bolacothrips striatopennata

(Schmutz, 1913)

1B0DF840-F5A6-522B-822E-D40894E999B6

https://thrips.info/wiki/Bolacothrips_striatopennatus

Thrips striatopennata Schmutz, 1913: 1002
Bolacothrips orientalis Priesner, 1935: 359
Bolacidothrips orizae Moulton, 1942: 10.

##### Materials

- **Type status:** Other material. **Occurrence:** recordedBy: Z.H.R; individualID: 2017-X-21|2017-X-22; individualCount: 2; sex: females; lifeStage: adults; occurrenceID: YAU5082020Tt50; **Taxon:** scientificNameAuthorship: *Bolacothripsstriatopennata* (Schmutz); **Location:** country: China; stateProvince: Yunnan; municipality: Xishuangbanna; locality: Mengla (Tropical Botanical Garden + Bubang Village); decimalLatitude: 21.957733; decimalLongitude: 100.459298; **Identification:** identifiedBy: Li Yajin; dateIdentified: 2018; identificationReferences: (ThripsWiki 2020); **Event:** samplingProtocol: sweeping and shaking; eventDate: 21/10/2017, 22/10/2017; **Record Level:** collectionID: thrips; institutionCode: YAU5082020; collectionCode: terebrantia; basisOfRecord: preserved specimen

##### Ecological interactions

###### Feeds on

leaves and collected from Poaceae.

##### Distribution

Described from Guam Island (USA). Recorded from Taiwan, China (Yunnan Province), Sri Lanka and Japan.

#### Bregmatothrips sinensis

Wang & Tong, 2016

8D1E311F-EBDE-5243-B1ED-E91577245436

https://thrips.info/wiki/Bregmatothrips_sinensis

##### Materials

- **Type status:** Other material. **Occurrence:** recordedBy: E.N & L.H; individualID: 2018-VIII-2; individualCount: 3; sex: females; lifeStage: adults; occurrenceID: YAU5082020Tt51; **Taxon:** scientificNameAuthorship: *Bregmatothripssinensis* Wang & Tong; **Location:** country: China; stateProvince: Yunnan; municipality: Xishuangbanna; locality: Mengla (Tropical Botanical Garden + Mansha Village), Jinghong (Nabanhe Protected Area + Botanical Garden); decimalLatitude: 21.957733; decimalLongitude: 100.459298; **Identification:** identifiedBy: Li Yajin; dateIdentified: 2018; identificationReferences: (ThripsWiki 2020); **Event:** samplingProtocol: sweeping and shaking; eventDate: 02/08/2018; **Record Level:** collectionID: thrips; institutionCode: YAU5082020; collectionCode: terebrantia; basisOfRecord: preserved specimen

##### Ecological interactions

###### Feeds on

leaves and collected from Poaceae.

##### Distribution

Described from Guangdong, China (Wang et al. 2016) and recorded in Xishuangbanna, Yunnan Province.

#### Chaetanaphothrips longisetis

Nonaka & Okajima, 1992

CAF0B2CB-D4C1-5425-A5D7-828ECC5B414B

https://thrips.info/wiki/Chaetanaphothrips_longisetis

##### Materials

- **Type status:** Other material. **Occurrence:** recordedBy: L.Y.J; individualID: 2017-III-11; individualCount: 2; sex: 1 male, 1 female; lifeStage: adults; occurrenceID: YAU5082020Tt52; **Taxon:** scientificNameAuthorship: *Chaetanaphothripslongisetis* Nonaka & Okajima; **Location:** country: China; stateProvince: Yunnan; municipality: Xishuangbanna; locality: Mengla (Bubang Village); decimalLatitude: 21.993361; decimalLongitude: 100.958531; **Identification:** identifiedBy: Li Yajin; dateIdentified: 2018; identificationReferences: (ThripsWiki 2020); **Event:** samplingProtocol: sweeping and shaking; eventDate: 11/03/2017; **Record Level:** collectionID: thrips; institutionCode: YAU5082020; collectionCode: terebrantia; basisOfRecord: preserved specimen

##### Ecological interactions

###### Feeds on

leaves and collected from oak tree.

##### Distribution

Described from Taiwan and distributed from China (Yunnan Province).

#### Chaetanaphothrips orchidii

(Moulton, 1907)

0105752E-B774-5FDD-90CA-37D8719C3948

https://thrips.info/wiki/Chaetanaphothrips_orchidii

Euthrips orchidii Moulton, 1907: 52
Euthrips marginemtorquens Karny, 1914: 362.

##### Materials

- **Type status:** Other material. **Occurrence:** recordedBy: L.Y.J. & Z.H.R; individualID: 2017-III-10|2017-III-12|2017-X-22; individualCount: 5; sex: females; lifeStage: adults; occurrenceID: YAU5082020Tt53; **Taxon:** scientificNameAuthorship: *Chaetanaphothripsorchidii* (Moulton); **Location:** country: China; stateProvince: Yunnan; municipality: Xishuangbanna; locality: Mengla (Tropical Botanical Garden + Mansha Village), Jinghong (Nabanhe Protected area+ Botanical garden); decimalLatitude: 21.978615; decimalLongitude: 100.942433; **Identification:** identifiedBy: Li Yajin; dateIdentified: 2018; identificationReferences: (ThripsWiki 2020); **Event:** samplingProtocol: sweeping and shaking; eventDate: 10/03/2017, 12/03/2017, 22/10/2017; **Record Level:** collectionID: thrips; institutionCode: YAU5082020; collectionCode: terebrantia; basisOfRecord: preserved specimen

##### Ecological interactions

###### Feeds on

leaves and collected from *Puerariamontana*.

##### Distribution

Described from Indonesia and widespread around the world.

#### Chaetanaphothrips querci

Kudo, 1985

2FF2ED9A-CE0E-529C-96F3-D612F7E23BA4

https://thrips.info/wiki/Chaetanaphothrips_querci

##### Materials

- **Type status:** Other material. **Occurrence:** recordedBy: L.H; individualID: 2017-X-24; individualCount: 1; sex: female; lifeStage: adults; occurrenceID: YAU5082020Tt54; **Taxon:** scientificNameAuthorship: *Chaetanaphothripsquerci* Kudo; **Location:** country: China; stateProvince: Yunnan; municipality: Xishuangbanna; locality: Mengla (Tropical Botanical Garden); decimalLatitude: 21.978615; decimalLongitude: 100.942433; **Identification:** identifiedBy: Li Yajin; dateIdentified: 2018; identificationReferences: (ThripsWiki 2020); **Event:** samplingProtocol: sweeping and shaking; eventDate: 24/10/2017; **Record Level:** collectionID: thrips; institutionCode: YAU5082020; collectionCode: terebrantia; basisOfRecord: preserved specimen

##### Ecological interactions

###### Feeds on

leaves and collected from tea leaves.

##### Distribution

Described from Japan and recorded from China.

#### Chaetanaphothrips theiperdus

(Karny, 1921)

1A00CDBE-4D2B-59C7-BEDE-BB4547614B26

https://thrips.info/wiki/Chaetanaphothrips_theiperdus

Anaphothrips theiperdus Karny, 1921: 69
Chaetanaphothrips taiwanus Sakimura, 1974: 319.

##### Materials

- **Type status:** Other material. **Occurrence:** recordedBy: L.H; individualID: 2017-X-24; individualCount: 1; sex: female; lifeStage: adults; occurrenceID: YAU5082020Tt56; **Taxon:** scientificNameAuthorship: *Chaetanaphothripstheiperdus* (Karny); **Location:** country: China; stateProvince: Yunnan; municipality: Xishuangbanna; locality: Mengla (Tropical Botanical Garden); decimalLatitude: 22.027942; decimalLongitude: 100.891984; **Identification:** identifiedBy: Li Yajin; dateIdentified: 2018; identificationReferences: (ThripsWiki 2020); **Event:** samplingProtocol: sweeping and shaking; eventDate: 24/10/2017; **Record Level:** collectionID: thrips; institutionCode: YAU5082020; collectionCode: terebrantia; basisOfRecord: preserved specimen

##### Ecological interactions

###### Feeds on

leaves and collected from tea leaves.

##### Distribution

Described from Indonesia (Java). Recorded from Japan (Kannanzan), Taiwan and China (Yunnan Province).

#### Cricothrips bourbonensis

(Bournier & Bournier, 1988)

548BC7E0-95A6-5F1F-A618-AC1DF571A05B

https://thrips.info/wiki/Cricothrips_bourbonensis

Moundiella bourbonensis Bournier & Bournier, 1988: 68.

##### Materials

- **Type status:** Other material. **Occurrence:** recordedBy: Z.C.H; individualID: 2017-III-10|2018-X-8; individualCount: 4; sex: 2 males, 2 females; lifeStage: adults; occurrenceID: YAU5082020Tt55; **Taxon:** scientificNameAuthorship: *Cricothripsbourbonensis* (Bournier & Bournier); **Location:** country: China; stateProvince: Yunnan; municipality: Xishuangbanna; locality: Mengla (Tropical Botanical Garden); decimalLatitude: 21.978615; decimalLongitude: 100.942433; **Identification:** identifiedBy: Li Yajin; dateIdentified: 2018; identificationReferences: (ThripsWiki 2020); **Event:** samplingProtocol: sweeping and shaking; eventDate: 10/03/2017, 08/10/2018; **Record Level:** collectionID: thrips; institutionCode: YAU5082020; collectionCode: terebrantia; basisOfRecord: preserved specimen

##### Ecological interactions

###### Feeds on

leaves and collected from mosses.

##### Distribution

Described from Reunion Island (Mare Longue) and recorded from southern China.

#### Danothrips theivorus

(Karny, 1921)

21C52117-2864-53BA-A448-D757099FE247

https://thrips.info/wiki/Danothrips_theivorus

Anaphothrips theivorus Karny, 1921: 75
Danothrips dianellae Zhang & Tong, 1991: 465.

##### Materials

- **Type status:** Other material. **Occurrence:** recordedBy: S.B & Z.H.R; individualID: 2017-III-10|2017-X-22; individualCount: 4; sex: females; lifeStage: adults; occurrenceID: YAU5082020Tt57; **Taxon:** scientificNameAuthorship: *Danothripstheivorus* (Karny); **Location:** country: China; stateProvince: Yunnan; municipality: Xishuangbanna; locality: Mengla (Tropical Botanical Garden); decimalLatitude: 22.03357; decimalLongitude: 100.933953; **Identification:** identifiedBy: Li Yajin; dateIdentified: 2018; identificationReferences: (ThripsWiki 2020); **Event:** samplingProtocol: sweeping and shaking; eventDate: 10/03/2017, 22/10/2018; **Record Level:** collectionID: thrips; institutionCode: YAU5082020; collectionCode: terebrantia; basisOfRecord: preserved specimen

##### Ecological interactions

###### Feeds on

flowers, young fruits, leaves. Collected from banana and camphor tree.

##### Distribution

Described from Indonesia and recorded from China (Guangdong and Yunnan Provinces).

#### Dendrothripoides poni

Kudo, 1977

99CB1252-21F4-5B4F-8C25-6D29BE3CFCC8

https://thrips.info/wiki/Dendrothripoides_poni

##### Materials

- **Type status:** Other material. **Occurrence:** recordedBy: L.Y.J; individualID: 2018-V-28; individualCount: 7; sex: 2 males, 5 females; lifeStage: adults; occurrenceID: YAU5082020Tt58; **Taxon:** scientificNameAuthorship: *Dendrothripoidesponi* Kudo; **Location:** country: China; stateProvince: Yunnan; municipality: Xishuangbanna; locality: Menghai, Jinghong; decimalLatitude: 21.99068; decimalLongitude: 101.00438; **Identification:** identifiedBy: Li Yajin; dateIdentified: 2018; identificationReferences: (ThripsWiki 2020); **Event:** samplingProtocol: sweeping and shaking; eventDate: 28/05/2018; **Record Level:** collectionID: thrips; institutionCode: YAU5082020; collectionCode: terebrantia; basisOfRecord: preserved specimen

##### Ecological interactions

###### Feeds on

leaves, collected from Poaceae and Sterculiaceae.

##### Distribution

Described from Thailand. Recorded from Malaysia and southern China.

#### Dendrothripoides innoxius

(Karny, 1914)

58DDD448-587F-5C7E-AB1E-4065CCB1B630

https://thrips.info/wiki/Dendrothripoides_innoxius

Euthrips innoxius Karny, 1914: 359
Dendrothripoides ipomoeae Bagnall, 1923: 625
Tryphactothrips mediosignatus Karny, 1925: 34
Tryphactothrips mundus Karny, 1926: 190 | Heliothrips ipomeae Bondar, 1930: 18
Scirtothrips gladiiseta Girault, 1933: 2.

##### Materials

- **Type status:** Other material. **Occurrence:** recordedBy: L.Y.J & Y.X.Q; individualID: 2018-V-28|2017-III-11|2017-X-24; individualCount: 12; sex: 3 males, 9 females; lifeStage: adults; occurrenceID: YAU5082020Tt59; **Taxon:** scientificNameAuthorship: *Dendrothripoidesinnoxius* (Karny); **Location:** country: China; stateProvince: Yunnan; municipality: Xishuangbanna; locality: Jinghong; decimalLatitude: 21.996042; decimalLongitude: 101.000931; **Identification:** identifiedBy: Li Yajin; dateIdentified: 2018; identificationReferences: (ThripsWiki 2020); **Event:** samplingProtocol: sweeping and shaking; eventDate: 1/03/2017, 24/10/2017, 28/05/2018; **Record Level:** collectionID: thrips; institutionCode: YAU5082020; collectionCode: terebrantia; basisOfRecord: preserved specimen

##### Ecological interactions

###### Feeds on

leaves and collected from potato leaves.

##### Distribution

Described from Indonesia. Recorded from India, China, Brazil, Australia and Nepal. Widespread in the Oriental and Pacific Regions.

#### Dichromothrips nakahari

Mound, 1976

198E1756-7F3C-5A44-A9F4-25AB72136D0F

https://thrips.info/wiki/Dichromothrips_nakahari

##### Materials

- **Type status:** Other material. **Occurrence:** recordedBy: S.S.Q; individualID: 2011-X-2; individualCount: 3; sex: females; lifeStage: adults; occurrenceID: YAU5082020Tt60; **Taxon:** scientificNameAuthorship: *Dichromothripsnakahari* Mound; **Location:** country: China; stateProvince: Yunnan; municipality: Xishuangbanna; locality: Xishuangbanna (Different sites); decimalLatitude: 21.980492; decimalLongitude: 100.999494; **Identification:** identifiedBy: Li Yajin; dateIdentified: 2018; identificationReferences: (ThripsWiki 2020); **Event:** samplingProtocol: sweeping and shaking; eventDate: 02/10/2011; **Record Level:** collectionID: thrips; institutionCode: YAU5082020; collectionCode: terebrantia; basisOfRecord: preserved specimen

##### Ecological interactions

###### Feeds on

flowers and collected from dendrobium.

##### Distribution

Described from India. Recorded from China and USA.

##### Notes

New record for China

##### Diagnosis

**Figure 17.**
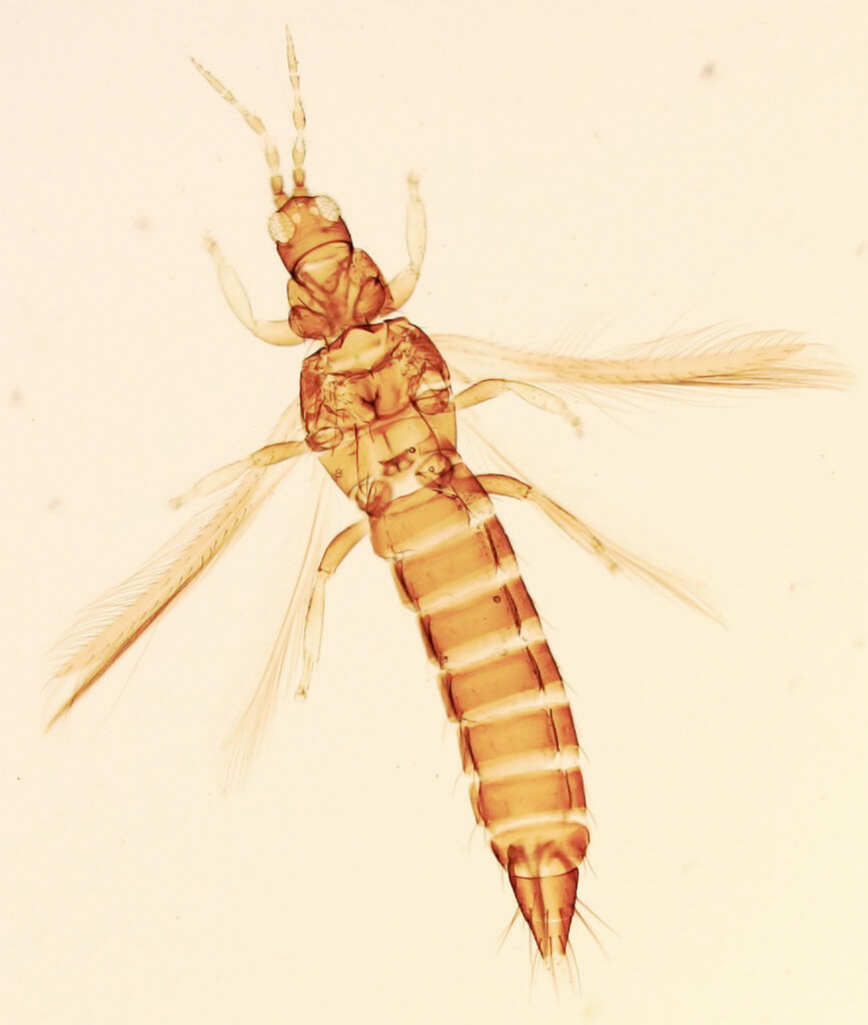
Female of *Dichromothripsnakahari* Mound, 1976.

Female macropterous; body dark brown (Fig. 17); head weakly prolonged in front of eyes; antennae 8-segmented, segment I without paired dorso-apical setae, III and IV with long apical neck and long forked sense-cones, III–V with some microtrichial rows on both surfaces; pronotum with two pairs of long postero-angular setae sub-equally in length; posterolateral margins of tergites with well-developed michrotrichia; metanotum weakly reticulated; abdominal tergites I-VIII without sculpture medially.

#### Dichromothrips smithi

(Zimmermann, 1900)

8EC795B7-5382-5A7B-85F2-A12B3CB4AEAA

https://thrips.info/wiki/Dichromothrips_smithi

Physopus smithi Zimmermann, 1900: 10.

##### Materials

- **Type status:** Other material. **Occurrence:** recordedBy: X.Y.L; individualID: 2017-X-25; individualCount: 9; sex: 4 males, 5 females; lifeStage: adults; occurrenceID: YAU5082020Tt61; **Taxon:** scientificNameAuthorship: *Dichromothripssmithi* (Zimmermann); **Location:** country: China; stateProvince: Yunnan; municipality: Xishuangbanna; locality: Jinghong (Mansha Village); decimalLatitude: 21.967621; decimalLongitude: 100.806322; **Identification:** identifiedBy: Li Yajin; dateIdentified: 2018; identificationReferences: (ThripsWiki 2020); **Event:** samplingProtocol: sweeping and shaking; eventDate: 25/10/2017; **Record Level:** collectionID: thrips; institutionCode: YAU5082020; collectionCode: terebrantia; basisOfRecord: preserved specimen

##### Ecological interactions

###### Feeds on

flowers, young fruits, leaves. Collected from dendrobium.

##### Distribution

Described from Indonesia. Recorded from India, Malaysia, Solomon Islands, Tokyo, Japan, Taiwan and China (Yunnan Province).

#### Echinothrips americanus

Morgan, 1913

6421E7D0-271B-5AA5-8DB4-EDB26679B353

https://thrips.info/wiki/Echinothrips_americanus

Dictyothrips floridensis Watson, 1919: 2.

##### Materials

- **Type status:** Other material. **Occurrence:** recordedBy: X.Y.L; individualID: 2017-III-9|2017-X-25; individualCount: 11; sex: 2 males, 9 females; lifeStage: adults; occurrenceID: YAU5082020Tt62; **Taxon:** scientificNameAuthorship: *Echinothripsamericanus* Morgan; **Location:** country: China; stateProvince: Yunnan; municipality: Xishuangbanna; locality: Xishuangbanna (Different sites); decimalLatitude: 21.920687; decimalLongitude: 101.186341; **Identification:** identifiedBy: Li Yajin; dateIdentified: 2018; identificationReferences: (ThripsWiki 2020); **Event:** samplingProtocol: sweeping and shaking; eventDate: 09/03/2017, 25/10/2017; **Record Level:** collectionID: thrips; institutionCode: YAU5082020; collectionCode: terebrantia; basisOfRecord: preserved specimen

##### Ecological interactions

###### Feeds on

leaves and collected from *Alocasiamacrorrhizos* (Araceae).

##### Distribution

Described in Florida. Introduced and widespread from different parts of the world.

#### Ernothrips immsi

(Bagnall, 1926)

617D1E79-DCC2-5039-B9E6-65648056917D

https://thrips.info/wiki/Ernothrips_immsi

Physothrips immsi Bagnall, 1926: 106.

##### Materials

- **Type status:** Other material. **Occurrence:** recordedBy: S.S.Q; individualID: 2009-III-08; individualCount: 5; sex: females; lifeStage: adults; occurrenceID: YAU5082020Tt63; **Taxon:** scientificNameAuthorship: *Ernothripsimmsi* (Bagnall); **Location:** country: China; stateProvince: Yunnan; municipality: Xishuangbanna; locality: Mengla (Menglun); decimalLatitude: 21.923772; decimalLongitude: 101.195684; **Identification:** identifiedBy: Li Yajin; dateIdentified: 2018; identificationReferences: (ThripsWiki 2020); **Event:** samplingProtocol: sweeping and shaking; eventDate: 08/03/2009; **Record Level:** collectionID: thrips; institutionCode: YAU5082020; collectionCode: terebrantia; basisOfRecord: preserved specimen

##### Ecological interactions

###### Feeds on

flowers and leaves, collected from citrus.

##### Distribution

Described from India and recorded from China.

#### Ernothrips lobatus

(Bhatti, 1967)

93F0F02D-38FF-5CDB-AA90-BE97A2576AD9

https://thrips.info/wiki/Ernothrips_lobatus

Thrips immsi Bagnall, 1926: 110 | Thrips (Ernothrips) lobatus Bhatti, 1967: 18.

##### Materials

- **Type status:** Other material. **Occurrence:** recordedBy: J.M.M & S.S.Q; individualID: 2010-I-23|2009-III-24|2009-X-11; individualCount: 11; sex: females; lifeStage: adults; occurrenceID: YAU5082020Tt64; **Taxon:** scientificNameAuthorship: *Ernothripslobatus* (Bhatti); **Location:** country: China; stateProvince: Yunnan; municipality: Xishuangbanna; locality: Xishuangbanna (Different sites); decimalLatitude: 21.923503; decimalLongitude: 101.201002; **Identification:** identifiedBy: Li Yajin; dateIdentified: 2018; identificationReferences: (ThripsWiki 2020); **Event:** samplingProtocol: sweeping and shaking; eventDate: 24/03/2009, 11/10/2009, 23/01/2010; **Record Level:** collectionID: thrips; institutionCode: YAU5082020; collectionCode: terebrantia; basisOfRecord: preserved specimen

##### Ecological interactions

###### Feeds on

flowers and leaves, collected from tea tree and chinese rose.

##### Distribution

Described from India. Recorded from China, Taiwan, Indonesia, Thailand, Japan and Malaysia.

##### Notes

This species has reported to be a successful pollinator in Dioscorea (Dioscoreaceae)（Li et al. 2014).

#### Ernothrips longitudinalis

Zhou, Zhang & Feng, 2008

725382CC-4023-5244-B063-023505DCDAED

https://thrips.info/wiki/Ernothrips_longitudinalis

##### Materials

- **Type status:** Other material. **Occurrence:** recordedBy: S.S.Q; individualID: 2009-II-16|2009-III-24; individualCount: 2; sex: females; lifeStage: adults; occurrenceID: YAU5082020Tt65; **Taxon:** scientificNameAuthorship: *Ernothripslongitudinalis* Zhou, Zhang & Feng; **Location:** country: China; stateProvince: Yunnan; municipality: Xishuangbanna; locality: Mengla (Tropical Botanical Garden); decimalLatitude: 21.926454; decimalLongitude: 101.257487; **Identification:** identifiedBy: Li Yajin; dateIdentified: 2018; identificationReferences: (ThripsWiki 2020); **Event:** samplingProtocol: sweeping and shaking; eventDate: 16/02/2009, 24/03/2009; **Record Level:** collectionID: thrips; institutionCode: YAU5082020; collectionCode: terebrantia; basisOfRecord: preserved specimen

##### Ecological interactions

###### Feeds on

flowers and leaves, collected from Brassicaceae.

##### Distribution

Described from Henan and distributed from Yunnan, China (Zhou et al. 2008).

#### Frankliniella fusca

(Hinds, 1902)

07D2281D-AE70-5DC6-873F-0A44170BAF63

https://thrips.info/wiki/Frankliniella_fusca

Euthrips fusca Hinds, 1902: 154
Euthrips nicotianae Hinds, 1905: 198
Scirtothrips owreyi Watson, 1924: 51.

##### Materials

- **Type status:** Other material. **Occurrence:** recordedBy: Z.H.R.; individualID: 2011-IX-15; individualCount: 9; sex: 2 males, 7 females; lifeStage: adults; occurrenceID: YAU5082020Tt66; **Taxon:** scientificNameAuthorship: *Frankliniellafusca* (Hinds); vernacularName: Tobacco thrips; **Location:** country: China; stateProvince: Yunnan; municipality: Xishuangbanna; locality: Mengla (Tropical Botanical Garden + Bubang Village), Jinghong (Nabanhe Protected Area + Botanical Garden); decimalLatitude: 21.923235; decimalLongitude: 101.25605; **Identification:** identifiedBy: Li Yajin; dateIdentified: 2018; identificationReferences: (ThripsWiki 2020); **Event:** samplingProtocol: sweeping and shaking; eventDate: 15/09/2011; **Record Level:** collectionID: thrips; institutionCode: YAU5082020; collectionCode: terebrantia; basisOfRecord: preserved specimen

##### Ecological interactions

###### Feeds on

flowers and fruits, collected from a wide range of host plants.

##### Distribution

Described from USA (Massachusetts) and worldwide distributed.

##### Notes

Vector of tomato spotted wilt virus (TSWV).

#### Frankliniella intonsa

(Trybom, 1895)

B5733F09-F569-51CA-A86E-5B6A89EA6220

https://thrips.info/wiki/Frankliniella_intonsa

Physopus vulgatissima var. albicornis Uzel, 1895: 96 | Physopusvulgatissimavar.fulvicornis Uzel, 1895: 96 | Physopusvulgatissimavar.nigropilosa Uzel, 1895: 96
Physapus brevistylis Karny, 1908: 278
Frankliniella breviceps Bagnall, 1911: 2
Frankliniella vicina Karny, 1922: 94 | Frankliniellaintonsavar.maritima Priesner, 1925: 165
Frankliniella formosae Moulton, 1928: 324 | Frankliniellaformosaef.tricolor Moulton, 1928: 325 | Frankliniellaintonsavar.rufula Keler, 1936: 104 | Frankliniellaintonsaf.norashensis Jakhontov & Jurbanov, 1957: 1279.

##### Materials

- **Type status:** Other material. **Occurrence:** recordedBy: L.Y.J; individualID: 2017-III-10; individualCount: 10; sex: 2 males, 8 females; lifeStage: adults; occurrenceID: YAU5082020Tt67; **Taxon:** scientificNameAuthorship: *Frankliniellaintonsa* (Trybom); **Location:** country: China; stateProvince: Yunnan; municipality: Xishuangbanna; locality: Menghai, Jinghong; decimalLatitude: 21.910225; decimalLongitude: 101.272866; **Identification:** identifiedBy: Li Yajin; dateIdentified: 2018; identificationReferences: (ThripsWiki 2020); **Event:** samplingProtocol: sweeping and shaking; eventDate: 10/03/2017; **Record Level:** collectionID: thrips; institutionCode: YAU5082020; collectionCode: terebrantia; basisOfRecord: preserved specimen

##### Ecological interactions

###### Feeds on

flowers and on leaves, collected from Cucurbitaceae, Fabaceae, Brassicaceae and Solanaceae.

##### Distribution

Described from Finland and worldwide distributed.

#### Frankliniella occidentalis

(Pergande, 1895)

B4C490D9-9B51-5CA3-9D69-9F76CB03B175

https://thrips.info/wiki/Frankliniella_occidentalis

Euthrips occidentalis Pergande, 1895: 392
Euthrips tritici californicus Moulton, 1911: 16
Euthrips helianthi Moulton, 1911: 40
Frankliniella tritici moultoni Hood, 1914: 38
Frankliniella nubila Treherne, 1924: 84. Synonymised by Nakahara (1997)
Frankliniella tritici maculata Priesner, 1925: 15. Synonymised by Nakahara (1997)
Frankliniella claripennis Morgan, 1925: 142
Frankliniella canadensis Morgan, 1925: 143. Synonym of *californicus* in Moulton (1948)
Frankliniella trehernei Morgan, 1925: 144
Frankliniella occidentalis brunnescens Priesner, 1932: 182
Frankliniella occidentalis dubia Priesner, 1932: 182
Frankliniella venusta Moulton, 1936: 172
Frankliniella conspicua Moulton, 1936: 173. Synonymised by Nakahara (1997)
Frankliniella chrysanthemi Kurosawa, 1941: 173
Frankliniella dahliae Moulton, 1948: 97
Frankliniella dianthi Moulton, 1948: 98. Synonymised by Mound and Marullo (1996)
Frankliniella syringae Moulton, 1948: 98. Synonymised by Mound and Marullo (1996)
Frankliniella umbrosa Moulton, 1948: 105. Synonymised by Nakahara (1997).

##### Materials

- **Type status:** Other material. **Occurrence:** recordedBy: K.B; individualID: 2015-X-6; individualCount: 6; sex: 1 male, 5 females; lifeStage: adults; occurrenceID: YAU5082020Tt68; **Taxon:** scientificNameAuthorship: *Frankliniellaoccidentalis* (Pergande); vernacularName: Western flowers thrips (WFT); **Location:** country: China; stateProvince: Yunnan; municipality: Xishuangbanna; locality: Jinghong (Mansha Village; decimalLatitude: 21.935976; decimalLongitude: 101.263955; **Identification:** identifiedBy: Li Yajin; dateIdentified: 2018; identificationReferences: (ThripsWiki 2020); **Event:** samplingProtocol: sweeping and shaking; eventDate: 06/10/2015; **Record Level:** collectionID: thrips; institutionCode: YAU5082020; collectionCode: terebrantia; basisOfRecord: preserved specimen

##### Ecological interactions

###### Feeds on

flowers and leaves, collected from potatoes and banana flowers.

##### Distribution

Described from USA (California) and worldwide distributed.

##### Notes

Vector of tomato spotted wilt virus (TSWV).

#### Isunidothrips serangga

Kudo, 1992

9B749F13-F079-5636-80A6-7FCC81453DEC

https://thrips.info/wiki/Isunidothrips_serangga

##### Materials

- **Type status:** Other material. **Occurrence:** recordedBy: X.Y.L; individualID: 2018-VI-2; individualCount: 1; sex: female; lifeStage: adults; occurrenceID: YAU5082020Tt69; **Taxon:** scientificNameAuthorship: *Isunidothripsseranggaserangga* Kudo; **Location:** country: China; stateProvince: Yunnan; municipality: Xishuangbanna; locality: Jinghong (Mansha Village; decimalLatitude: 21.920955; decimalLongitude: 101.289826; **Identification:** identifiedBy: Li Yajin; dateIdentified: 2018; identificationReferences: (ThripsWiki 2020); **Event:** samplingProtocol: sweeping and shaking; eventDate: 02/06/2018; **Record Level:** collectionID: thrips; institutionCode: YAU5082020; collectionCode: terebrantia; basisOfRecord: preserved specimen

##### Ecological interactions

###### Feeds on

leaves, collected from fronds of ferns and Cyperaceae.

##### Distribution

Described from Malaysia and recorded from southern China.

#### Lefroyothrips lefroyi

(Bagnall, 1913)

DA01F84A-564A-569B-A983-2EB7193BBD31

https://thrips.info/wiki/Lefroyothrips_lefroyi

Physothrips lefroyi Bagnall, 1913: 292
Taeniothrips cuscutae Priesner, 1938: 500 | Taeniothrips (Lefroyothrips) theiphilus Priesner, 1938: 501
Taeniothrips devii Arora & Bhatti, 1960: 141. Synonyms by Bhatti (1978).

##### Materials

- **Type status:** Other material. **Occurrence:** recordedBy: E.N, L.Y.J & Z.H.R; individualID: 2018-V-26|2017-X-22; individualCount: 732; sex: 121 males, 611 females; lifeStage: adults; occurrenceID: YAU5082020Tt70; **Taxon:** scientificNameAuthorship: *Lefroyothripslefroyi* (Bagnall); **Location:** country: China; stateProvince: Yunnan; municipality: Xishuangbanna; locality: Mengla (Tropical Botanical Garden); decimalLatitude: 21.706336; decimalLongitude: 101.511312; **Identification:** identifiedBy: Li Yajin; dateIdentified: 2018; identificationReferences: (ThripsWiki 2020); **Event:** samplingProtocol: sweeping and shaking; eventDate: 22/10/2017, 26/05/2018; **Record Level:** collectionID: thrips; institutionCode: YAU5082020; collectionCode: terebrantia; basisOfRecord: preserved specimen

##### Ecological interactions

###### Feeds on

flowers, collected from tea tree, mango and papaya.

##### Distribution

Described from India. Recorded from Indonesia, China and widely introduced.

#### Megalurothrips distalis

(Karny, 1913)

18AB792A-065A-5A89-B872-D0D4E4C175CD

https://thrips.info/wiki/Megalurothrips_distalis

Taeniothrips distalis Karny, 1913: 122
Physothrips brunneicornis Bagnall, 1916: 218
Taeniothrips infernalis Priesner, 1938: 472
Taeniothrips morosus Priesner, 1938: 476
Taeniothrips ditissimus Ananthakrishnan & Jagadish, 1966: 250.

##### Materials

- **Type status:** Other material. **Occurrence:** recordedBy: L.Y.J; individualID: 2017-V-21; individualCount: 26; sex: 7 males, 19 females; lifeStage: adults; occurrenceID: YAU5082020Tt71; **Taxon:** scientificNameAuthorship: *Megalurothripsdistalis* (Karny); **Location:** country: China; stateProvince: Yunnan; municipality: Xishuangbanna; locality: Jinghong (Mansha Village); decimalLatitude: 21.643996; decimalLongitude: 101.789571; **Identification:** identifiedBy: Li Yajin; dateIdentified: 2018; identificationReferences: (ThripsWiki 2020); **Event:** samplingProtocol: sweeping and shaking; eventDate: 21/05/2017; **Record Level:** collectionID: thrips; institutionCode: YAU5082020; collectionCode: terebrantia; basisOfRecord: preserved specimen

##### Ecological interactions

###### Feeds on

flowers, collected from *Erythrinavariegata* L., *Psidiumguava* and mango.

##### Distribution

Described from Japan and widely distributed.

##### Diagnosis

**Figure 18.**
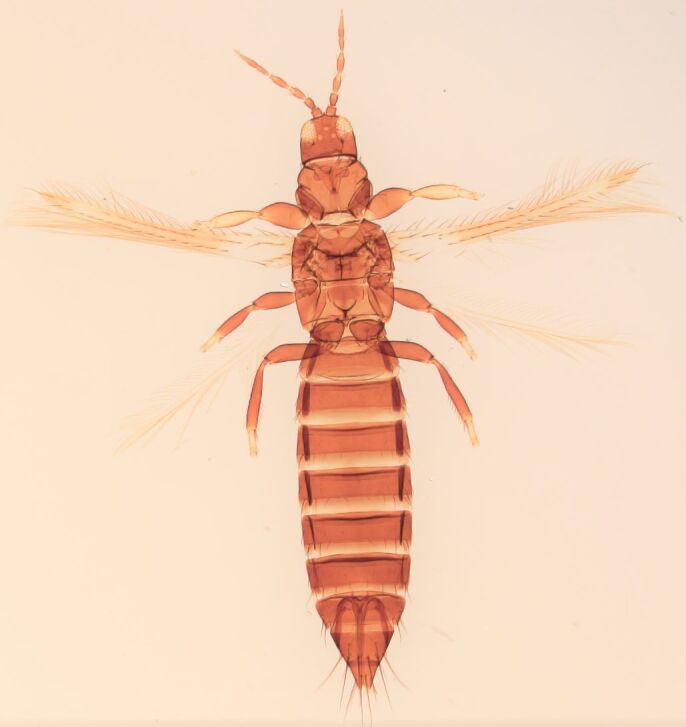
Female of *Megalurothripsdistalis* (Karny, 1913).

Female macropterous; Body dark brown (Fig. 18), head wider than long; eyes with five weakly-pigmented facets, ocellar setae I present, setae III elongate, presence of five pairs of postocular setae; antennae 8-segmented, segments III and IV with elongate forked sense cones, III-VI with some microtrichia on both surfaces, VI with an elongate sense cone at the base. Adult male with spear-shaped sternal discal setae.

#### Megalurothrips typicus

Bagnall, 1915

633BB536-855C-5616-BA91-314B62926BCD

https://thrips.info/wiki/Megalurothrips_typicus

Megalurothrips typicus Bagnall, 1915: 590
Megalurothrips setipennis Karny, 1925: 32
Taeniothrips varicornis Moulton, 1928: 292
Taeniothrips centrispinosus Priesner, 1938: 474.

##### Materials

- **Type status:** Other material. **Occurrence:** recordedBy: L.Y.J, X.Y.L & Z.H.R; individualID: 2017-X-23|2017-X-24|2017-X-22|2018-V-28; individualCount: 36; sex: 8 males, 28 females; lifeStage: adults; occurrenceID: YAU5082020Tt72; **Taxon:** scientificNameAuthorship: *Megalurothripstypicus* Bagnall; **Location:** country: China; stateProvince: Yunnan; municipality: Xishuangbanna; locality: Mengla (Tropical Botanical Garden); decimalLatitude: 21.851196; decimalLongitude: 100.953357; **Identification:** identifiedBy: Li Yajin; dateIdentified: 2018; identificationReferences: (ThripsWiki 2020); **Event:** samplingProtocol: sweeping and shaking; eventDate: 22/10/2017, 23/10/2017, 24/10/2017, 28/05/2018; **Record Level:** collectionID: thrips; institutionCode: YAU5082020; collectionCode: terebrantia; basisOfRecord: preserved specimen

##### Ecological interactions

###### Feeds on

flowers, collected from kalancho, mango and papaya.

##### Distribution

Described from Malaysia. Recorded from Indonesia, Taiwan and China.

#### Megalurothrips usitatus

(Bagnall, 1913)

EE32B86D-0D7B-5829-9FB8-A26A62B13E69

https://thrips.info/wiki/Megalurothrips_usitatus

Physothrips usitatus Bagnall, 1913: 293
Frankliniella nigricornis Schmutz, 1913: 1020
Frankliniella obscuricornis Schmutz, 1913: 1022
Frankliniella vitata Schmutz, 1913: 1023
Physothrips cinctipennis Bagnall, 1916: 217
Physothrips mjobergi Karny, 1920: 37
Taeniothrips longistylus Karny, 1922: 99.

##### Materials

- **Type status:** Other material. **Occurrence:** recordedBy: Y.X.Q; individualID: 2016-VIII-29|2016-VIII-25; individualCount: 39; sex: 10 males, 29 females; lifeStage: adults; occurrenceID: YAU5082020Tt73; **Taxon:** scientificNameAuthorship: *Megalurothripsusitatus* (Bagnall); **Location:** country: China; stateProvince: Yunnan; municipality: Xishuangbanna; locality: Jinghong (Mansha Village; decimalLatitude: 21.854416; decimalLongitude: 100.944158; **Identification:** identifiedBy: Li Yajin; dateIdentified: 2018; identificationReferences: (ThripsWiki 2020); **Event:** samplingProtocol: sweeping and shaking; eventDate: 25/08/2016, 29/06/2016; **Record Level:** collectionID: thrips; institutionCode: YAU5082020; collectionCode: terebrantia; basisOfRecord: preserved specimen

##### Ecological interactions

###### Feeds on

flowers, collected from Fabaceae, Poaceae and mango.

##### Distribution

Described from India and widely distributed.

#### Microcephalothrips abdominalis

(Crawford DL, 1910)

4380E1E3-C363-5292-94B9-145B871B34AF

https://thrips.info/wiki/Microcephalothrips_abdominalis

Thrips abdominalis Crawford DL, 1910: 157
Thrips femoralis Jones, 1912: 4
Thrips crenatus Watson, 1922: 35
Thrips microcephalus Priesner, 1923: 116 | Thrips (Ctenothripiella) gillettei Moulton, 1926: 126
Stylothrips brevipalpis Karny, 1926: 206
Paraphysopus burnsi Girault, 1927: 2
Thrips oklahomae Watson, 1931: 342
Microcephalothrips brevipalpis armatus Ananthakrishnan, 1956: 133
Aureothrips marigoldae Raizada, 1966: 278
Microcephalothrips chinensis Feng, 1998: 257
Microcephalothrips jigonshanensis Feng, 1998: 258
Microcephalothrips yanglinensis Feng, Zhang & Sha, 2002: 167.

##### Materials

- **Type status:** Other material. **Occurrence:** recordedBy: L.Y.J; individualID: 2018-VI-2; individualCount: 1; sex: female; lifeStage: adults; occurrenceID: YAU5082020Tt74; **Taxon:** scientificNameAuthorship: *Microcephalothripsabdominalis* (Crawford DL); **Location:** country: China; stateProvince: Yunnan; municipality: Xishuangbanna; locality: Mengla (Tropical Botanical Garden); decimalLatitude: 21.859515; decimalLongitude: 100.956519; **Identification:** identifiedBy: Li Yajin; dateIdentified: 2018; identificationReferences: (ThripsWiki 2020); **Event:** samplingProtocol: sweeping and shaking; eventDate: 02/06/2018; **Record Level:** collectionID: thrips; institutionCode: YAU5082020; collectionCode: terebrantia; basisOfRecord: preserved specimen

##### Ecological interactions

###### Feeds on

flowers and collected from sunflower.

##### Distribution

Described from Mexico and widely distributed.

#### Octothrips bhattii

(Wilson, 1972)

DFEE9300-F3EE-55D1-A3D3-E4DD187D5968

https://thrips.info/wiki/Octothrips_bhattii

Apollothrips bhattii Wilson, 1972: 52
Octothrips lygodii Mound, 2002: 219.

##### Materials

- **Type status:** Other material. **Occurrence:** recordedBy: L.Y.J; individualID: 2017-V-4; individualCount: 7; sex: 3 males, 4 females; lifeStage: adults; occurrenceID: YAU5082020Tt78; **Taxon:** scientificNameAuthorship: *Octothripsbhattii* (Wilson); **Location:** country: China; stateProvince: Yunnan; municipality: Xishuangbanna; locality: Jinghong (Nabanhe Protected Area); decimalLatitude: 22.011754; decimalLongitude: 100.785957; **Identification:** identifiedBy: Li Yajin; dateIdentified: 2018; identificationReferences: (ThripsWiki 2020); **Event:** samplingProtocol: sweeping and shaking; eventDate: 04/05/2017; **Record Level:** collectionID: thrips; institutionCode: YAU5082020; collectionCode: terebrantia; basisOfRecord: preserved specimen

##### Ecological interactions

###### Feeds on

leaves and collected from ferns.

##### Distribution

Described from India. Recorded from Hong Kong and southern China.

#### Organothrips longisetosus

(Zhang & Tong, 1992)

9454E6A2-7226-5D0E-B10A-7BF862B0657A

https://thrips.info/wiki/Organothrips_longisetosus

Graminothrips longisetosus Zhang & Tong, 1992: 84.

##### Materials

- **Type status:** Other material. **Occurrence:** recordedBy: E.N & L.H; individualID: 2018-VII-2; individualCount: 7; sex: 4 males, 5 females; lifeStage: adults; occurrenceID: YAU5082020Tt80; **Taxon:** scientificNameAuthorship: *Organothripslongisetosus* (Zhang & Tong); **Location:** country: China; stateProvince: Yunnan; municipality: Xishuangbanna; locality: Jinghong (Nabanhe Protected Area); decimalLatitude: 22.001969; decimalLongitude: 100.795012; **Identification:** identifiedBy: Li Yajin; dateIdentified: 2018; identificationReferences: (ThripsWiki 2020); **Event:** samplingProtocol: sweeping and shaking; eventDate: 02/07/2018; **Record Level:** collectionID: thrips; institutionCode: YAU5082020; collectionCode: terebrantia; basisOfRecord: preserved specimen

##### Ecological interactions

###### Feeds on

leaves and stem. Collected from *Arthraxonhispidus* (Poaceae).

##### Distribution

Described from Guangxi and distributed from southern China.

#### Plesiothrips perplexus

(Beach, 1896)

8EACD234-02C6-5056-9783-0E87F6779BEC

https://thrips.info/wiki/Plesiothrips_perplexus

Sericothrips perplexus Beach, 1896: 216
Thrips panicus Moulton, 1929: 61.

##### Materials

- **Type status:** Other material. **Occurrence:** recordedBy: Z.H.R; individualID: 2017-X-22; individualCount: 1; sex: female; lifeStage: adults; occurrenceID: YAU5082020Tt81; **Taxon:** scientificNameAuthorship: *Plesiothripsperplexus* (Beach); **Location:** country: China; stateProvince: Yunnan; municipality: Xishuangbanna; locality: Mengla (Tropical Botanical Garden); decimalLatitude: 21.963829; decimalLongitude: 100.64345; **Identification:** identifiedBy: Li Yajin; dateIdentified: 2018; identificationReferences: (ThripsWiki 2020); **Event:** samplingProtocol: sweeping and shaking; eventDate: 22/10/2017; **Record Level:** collectionID: thrips; institutionCode: YAU5082020; collectionCode: terebrantia; basisOfRecord: preserved specimen

##### Ecological interactions

###### Feeds on

flowers and collected from corns.

##### Distribution

Described from Iowa. Recorded from Hawaii and southern China.

#### Rhamphothrips aureus

(Ananthakrishnan, 1954)

87B817E6-59F8-5599-981D-9820E786BB62

https://thrips.info/wiki/Rhamphothrips_aureus

Perissothrips aureus Ananthakrishnan, 1954: 159
Perissothrips hartwigi Bhatti, 1967: 12.

##### Materials

- **Type status:** Other material. **Occurrence:** recordedBy: L.Y.J; individualID: 2017-III-10; individualCount: 2; sex: males; lifeStage: adults; occurrenceID: YAU5082020Tt82; **Taxon:** scientificNameAuthorship: *Rhamphothripsaureus* (Ananthakrishnan); **Location:** country: China; stateProvince: Yunnan; municipality: Xishuangbanna; locality: Mengla (Menglun); decimalLatitude: 22.043353; decimalLongitude: 100.917923; **Identification:** identifiedBy: Li Yajin; dateIdentified: 2018; identificationReferences: (ThripsWiki 2020); **Event:** samplingProtocol: sweeping and shaking; eventDate: 10/03/2017; **Record Level:** collectionID: thrips; institutionCode: YAU5082020; collectionCode: terebrantia; basisOfRecord: preserved specimen

##### Ecological interactions

###### Feeds on

leaves, collected from bamboo and Euphorbiaceae.

##### Distribution

Described from India and recorded from southern China.

#### Rhamphothrips bruceae

Li & Zhang, 2018

ADEE2BE3-E81A-5ECC-9356-876603219090

https://www.biotaxa.org/Zootaxa/article/view/zootaxa.4446.3.6/0

##### Materials

- **Type status:** Other material. **Occurrence:** recordedBy: X.Y,L; individualID: 2018-VI-1; individualCount: 1; sex: female; lifeStage: adults; occurrenceID: YAU5082020Tt83; **Taxon:** scientificNameAuthorship: *Rhamphothripsbruceae* Li & Zhang; **Location:** country: China; stateProvince: Yunnan; municipality: Xishuangbanna; locality: Jinghong (Naban He Protected Area); decimalLatitude: 22.010116; decimalLongitude: 100.958167; **Identification:** identifiedBy: Li Yajin; dateIdentified: 2018; identificationReferences: (ThripsWiki 2020); **Event:** samplingProtocol: sweeping and shaking; eventDate: 01/06/2018; **Record Level:** collectionID: thrips; institutionCode: YAU5082020; collectionCode: terebrantia; basisOfRecord: preserved specimen

##### Ecological interactions

###### Feeds on

leaves and collected from Brucea (Simaroubaceae).

##### Distribution

Described from China (Xishuangbanna) (Li et al. 2018a).

#### Rhamphothrips parviceps

(Hood, 1919)

F4F98330-B94A-520E-AA2E-E5DF90095D22

https://thrips.info/wiki/Rhamphothrips_parviceps

Perissothrips parviceps Hood, 1919: 92.

##### Materials

- **Type status:** Other material. **Occurrence:** recordedBy: S.S.Q; individualID: 2009-IV-26; individualCount: 1; sex: female; lifeStage: adults; occurrenceID: YAU5082020Tt84; **Taxon:** scientificNameAuthorship: *Rhamphothripsparviceps* (Hood); **Location:** country: China; stateProvince: Yunnan; municipality: Xishuangbanna; locality: Jinghong (Naban He Protected Area); decimalLatitude: 22.004755; decimalLongitude: 100.922522; **Identification:** identifiedBy: Li Yajin; dateIdentified: 2018; identificationReferences: (ThripsWiki 2020); **Event:** samplingProtocol: sweeping and shaking; eventDate: 26/04/2009; **Record Level:** collectionID: thrips; institutionCode: YAU5082020; collectionCode: terebrantia; basisOfRecord: preserved specimen

##### Ecological interactions

###### Feeds on

leaves and collected from Fabaceae.

##### Distribution

Described from India and recorded from China (Xishuangbanna) (Li et al. 2018a).

#### Rhamphothrips santokhi

Kulshrestha & Vijay Veer, 1984

EDD1504D-06C5-503F-BBED-31DC36331337

https://thrips.info/wiki/Rhamphothrips_santokhi

##### Materials

- **Type status:** Other material. **Occurrence:** recordedBy: L.Y.J; individualID: 2017-III-10; individualCount: 1; sex: female; lifeStage: adults; occurrenceID: YAU5082020Tt85; **Taxon:** scientificNameAuthorship: *Rhamphothripssantokhi* Kulshrestha & Vijay Veer; **Location:** country: China; stateProvince: Yunnan; municipality: Xishuangbanna; locality: Mengla (Bubang Village); decimalLatitude: 21.995104; decimalLongitude: 100.879979; **Identification:** identifiedBy: Li Yajin; dateIdentified: 2018; identificationReferences: (ThripsWiki 2020); **Event:** samplingProtocol: sweeping and shaking; eventDate: 10/03/2017; **Record Level:** collectionID: thrips; institutionCode: YAU5082020; collectionCode: terebrantia; basisOfRecord: preserved specimen

##### Ecological interactions

###### Feeds on

leaves and collected from Euphorbiaceae.

##### Distribution

Described from India and recorded from southern China.

#### Salpingothrips aimotofus

Kudo, 1972

91326844-F358-52C2-A239-036B79461F04

https://thrips.info/wiki/Salpingothrips_aimotofus

##### Materials

- **Type status:** Other material. **Occurrence:** recordedBy: Z.H.R; individualID: 2017-X-22; individualCount: 1; sex: female; lifeStage: adults; occurrenceID: YAU5082020Tt86; **Taxon:** scientificNameAuthorship: *Salpingothripsaimotofus* Kudo; **Location:** country: China; stateProvince: Yunnan; municipality: Xishuangbanna; locality: Mengla (Tropical Botanical Garden); decimalLatitude: 21.973654; decimalLongitude: 100.942069; **Identification:** identifiedBy: Li Yajin; dateIdentified: 2018; identificationReferences: (ThripsWiki 2020); **Event:** samplingProtocol: sweeping and shaking; eventDate: 22/10/2017; **Record Level:** collectionID: thrips; institutionCode: YAU5082020; collectionCode: terebrantia; basisOfRecord: preserved specimen

##### Ecological interactions

###### Feeds on

leaves and collected from *Puerariamontana*.

##### Distribution

Described from Japan. Recorded from Georgia and southern China.

#### Scirtothrips dorsalis

Hood, 1919

7F4975B6-5B84-5C87-91CC-B321F89326DD

https://thrips.info/wiki/Scirtothrips_dorsalis

Scirtothrips dorsalis Hood, 1919: 90
Heliothrips minutissimus Bagnall, 1919: 260
Anaphothrips andreae Karny, 1925: 24
Neophysopus fragariae Girault, 1927: 1. Synonymised by Mound & Palmer (1981) | Scirtothripsdorsalisvar.padmae Ramakrishna, 1942: 169.

##### Materials

- **Type status:** Other material. **Occurrence:** recordedBy: E.N & L.H; individualID: 2018-V-27; individualCount: 17; sex: 14 males, 3 females; lifeStage: adults; occurrenceID: YAU5082020Tt87; **Taxon:** scientificNameAuthorship: *Scirtothripsdorsalis* Hood; **Location:** country: China; stateProvince: Yunnan; municipality: Xishuangbanna; locality: Mengla (Tropical Botanical Garden); decimalLatitude: 22.005827; decimalLongitude: 100.924822; **Identification:** identifiedBy: Li Yajin; dateIdentified: 2018; identificationReferences: (ThripsWiki 2020); **Event:** samplingProtocol: sweeping and shaking; eventDate: 27/05/2018; **Record Level:** collectionID: thrips; institutionCode: YAU5082020; collectionCode: terebrantia; basisOfRecord: preserved specimen

##### Ecological interactions

###### Feeds on

leaves and collected from tea tree, pepper and beans.

##### Distribution

Described from Australia. Recorded from India, Indonesia and southern China.

#### Scolothrips asura

Ramakrishna & Margabandhu, 1931

7B0CEBF2-1C86-5713-8077-E4435CE219C3

https://thrips.info/wiki/Scolothrips_asura

Scolothrips asura Ramakrishna & Margabandhu, 1931: 1035
Scolothrips quadrinotata Han & Zhang, 1982: 53.

##### Materials

- **Type status:** Other material. **Occurrence:** recordedBy: L.Y.J; individualID: 2017-III-11; individualCount: 10; sex: 2 males, 8 females; lifeStage: adults; occurrenceID: YAU5082020Tt88; **Taxon:** scientificNameAuthorship: *Scolothripsasura* Ramakrishna & Margabandhu; **Location:** country: China; stateProvince: Yunnan; municipality: Xishuangbanna; locality: Mengla (Tropical Botanical Garden); decimalLatitude: 22.109802; decimalLongitude: 100.87078; **Identification:** identifiedBy: Li Yajin; dateIdentified: 2018; identificationReferences: (ThripsWiki 2020); **Event:** samplingProtocol: sweeping and shaking; eventDate: 11/03/2017; **Record Level:** collectionID: thrips; institutionCode: YAU5082020; collectionCode: terebrantia; basisOfRecord: preserved specimen

##### Ecological interactions

###### Feeds on

small arthropods.

##### Distribution

Described from India. Recorded from China, Japan, Taiwan, Thailand and northern Australia.

#### Scolothrips takahashii

Priesner, 1950

14F11055-39B1-58D0-850F-2F30021666AE

https://thrips.info/wiki/Scolothrips_takahashii

Scolothrips takahashii Priesner, 1950: 52 | Scolothrips priesneri Sakimura, 1954: 357.

##### Materials

- **Type status:** Other material. **Occurrence:** recordedBy: E.N & L.H; individualID: 2020-VI-24; individualCount: 33; sex: 9 males, 24 females; lifeStage: adults; occurrenceID: YAU5082020Tt89; **Taxon:** scientificNameAuthorship: *Scolothripstakahashii* Priesner; **Location:** country: China; stateProvince: Yunnan; municipality: Xishuangbanna; locality: Xishuangbanna (Different sites); decimalLatitude: 22.177291; decimalLongitude: 100.890327; **Identification:** identifiedBy: Li Yajin; dateIdentified: 2020; identificationReferences: (ThripsWiki 2020); **Event:** samplingProtocol: sweeping and shaking; eventDate: 24/06/2020; **Record Level:** collectionID: thrips; institutionCode: YAU5082020; collectionCode: terebrantia; basisOfRecord: preserved specimen

##### Ecological interactions

###### Feeds on

small arthropods.

##### Distribution

Described from Taiwan and Hawaii USA. Widely distributed in China.

#### Sorghothrips meishanensis

Chen, 1977

5D2D4E5F-3A3B-5EA2-932A-7413AEE2E636

https://thrips.info/wiki/Sorghothrips_meishanensis

##### Materials

- **Type status:** Other material. **Occurrence:** recordedBy: L.Y.J; individualID: 2017-III-11; individualCount: 2; sex: females; lifeStage: adults; occurrenceID: YAU5082020Tt90; **Taxon:** scientificNameAuthorship: *Sorghothripsmeishanensis* Chen; **Location:** country: China; stateProvince: Yunnan; municipality: Xishuangbanna; locality: Jinghong (Nabanhe Protected Area + Mansha Village); decimalLatitude: 22.177291; decimalLongitude: 100.861869; **Identification:** identifiedBy: Li Yajin; dateIdentified: 2018; identificationReferences: (ThripsWiki 2020); **Event:** samplingProtocol: sweeping and shaking; eventDate: 11/03/2017; **Record Level:** collectionID: thrips; institutionCode: YAU5082020; collectionCode: terebrantia; basisOfRecord: preserved specimen

##### Ecological interactions

###### Feeds on

leaves, collected from corns and sugar-cane.

##### Distribution

Described from Taiwan and recorded from south China.

#### Stenchaetothrips biformis

(Bagnall, 1913)

2502B5BD-347C-5B87-BC1D-C09203CB2255

https://thrips.info/wiki/Stenchaetothrips_biformis

Bagnallia biformis Bagnall, 1913: 237
Bagnallia adusta Bagnall, 1913: 238
Bagnallia melanurus Bagnall, 1913: 238 | Thrips (Bagnallia) oryzae Williams, 1916: 353
Thrips holorphnus Karny, 1925: 15
Plesiothrips o Girault, 1929: 1
Thrips dobrogensis Knechtel, 1964: 479
Chloethrips blandus zur Strassen, 1975: 78.

##### Materials

- **Type status:** Other material. **Occurrence:** recordedBy: S.S.Q & L.Y.J; individualID: 2009-IV-6|2017-X-21; individualCount: 16; sex: 10 males, 6 female; lifeStage: adults; occurrenceID: YAU5082020Tt91; **Taxon:** scientificNameAuthorship: *Stenchaetothripsbiformis* (Bagnall); **Location:** country: China; stateProvince: Yunnan; municipality: Xishuangbanna; locality: Mengla; decimalLatitude: 22.179968; decimalLongitude: 100.849508; **Identification:** identifiedBy: Li Yajin; dateIdentified: 2018; identificationReferences: (ThripsWiki 2020); **Event:** samplingProtocol: sweeping and shaking; eventDate: 06/04/2009, 21/10/2017; **Record Level:** collectionID: thrips; institutionCode: YAU5082020; collectionCode: terebrantia; basisOfRecord: preserved specimen

##### Ecological interactions

###### Feeds on

leaves, collected from bamboo and Poaceae

##### Distribution

Described from England and widely distributed from different parts of the world.

#### Stenchaetothrips brochus

Wang, 2000

5C9EAE6C-F9CD-5876-8108-DEC8BA24FFC8

https://thrips.info/wiki/Stenchaetothrips_brochus

##### Materials

- **Type status:** Other material. **Occurrence:** recordedBy: L.Y.J; individualID: 2017-IV-14; individualCount: 3; sex: 2 males, 1 females; lifeStage: adults; occurrenceID: YAU5082020Tt92; **Taxon:** scientificNameAuthorship: *Stenchaetothripsbrochus* Wang; **Location:** country: China; stateProvince: Yunnan; municipality: Xishuangbanna; locality: Jinghong (Mansha Village); decimalLatitude: 21.922967; decimalLongitude: 101.184971; **Identification:** identifiedBy: Li Yajin; dateIdentified: 2018; identificationReferences: (ThripsWiki 2020); **Event:** samplingProtocol: sweeping and shaking; eventDate: 14/04/2017; **Record Level:** collectionID: thrips; institutionCode: YAU5082020; collectionCode: terebrantia; basisOfRecord: preserved specimen

##### Ecological interactions

###### Feeds on

leaves and collected from bamboo.

##### Distribution

Described from Taiwan and distributed from southern China.

#### Stenchaetothrips cymbopogoni

Zhang & Tong, 1990

9BDE990C-10DC-5EEB-98F0-CAD4FAF5EAA3

https://thrips.info/wiki/Stenchaetothrips_cymbopogoni

##### Materials

- **Type status:** Other material. **Occurrence:** recordedBy: S.S.Q; individualID: 2010-XII-27; individualCount: 4; sex: females; lifeStage: adults; occurrenceID: YAU5082020Tt93; **Taxon:** scientificNameAuthorship: *Stenchaetothripscymbopogoni* Zhang & Tong; **Location:** country: China; stateProvince: Yunnan; municipality: Xishuangbanna; locality: Mengla (Tropical Botanical Garden + Bubang Village), Jinghong (Nabanhe Protected Area + Botanical Garden); decimalLatitude: 21.918541; decimalLongitude: 101.184828; **Identification:** identifiedBy: Li Yajin; dateIdentified: 2018; identificationReferences: (ThripsWiki 2020); **Event:** samplingProtocol: sweeping and shaking; eventDate: 27/12/2010; **Record Level:** collectionID: thrips; institutionCode: YAU5082020; collectionCode: terebrantia; basisOfRecord: preserved specimen

##### Ecological interactions

###### Feeds on

leaves, collected from mango, citronella and sugar-cane.

##### Distribution

Described from Hainan and distributed from south China.

#### Stenchaetothrips minutus

(Deventer, 1906)

C158B4E4-2EF0-53A5-AB39-20AFCAA070C6

https://thrips.info/wiki/Stenchaetothrips_minutus

Thrips minutus Deventer, 1906: 281
Thrips puttemansi Costa Lima, 1926: 32
Thrips saccharoni Moulton, 1928: 111
Fulmekiola saccharicida Ramakrishna & Margabandhu, 1939: 23.

##### Materials

- **Type status:** Other material. **Occurrence:** recordedBy: L.Y.J; individualID: 2017-III-10; individualCount: 3; sex: females; lifeStage: adults; occurrenceID: YAU5082020Tt94; **Taxon:** scientificNameAuthorship: *Stenchaetothripsminutus* (Deventer); **Location:** country: China; stateProvince: Yunnan; municipality: Xishuangbanna; locality: Mengla (Tropical Botanical Garden); decimalLatitude: 21.914651; decimalLongitude: 101.186983; **Identification:** identifiedBy: Li Yajin; dateIdentified: 2018; identificationReferences: (ThripsWiki 2020); **Event:** samplingProtocol: sweeping and shaking; eventDate: 10/03/2017; **Record Level:** collectionID: thrips; institutionCode: YAU5082020; collectionCode: terebrantia; basisOfRecord: preserved specimen

##### Ecological interactions

###### Feeds on

leaves, collected from bamboo, Poaceae and prunella.

##### Distribution

Described from Indonesia. Recorded from Hawaii and China.

#### Taeniothrips musae

(Zhang & Tong, 1990)

39D6CDC6-FD92-58B9-B765-4302549FACC5

https://thrips.info/wiki/Taeniothrips_musae

Javathrips musae Zhang & Tong, 1990: 193.

##### Materials

- **Type status:** Other material. **Occurrence:** recordedBy: Z.W.Q; individualID: not found; individualCount: 8; sex: 2 males, 6 females; lifeStage: adults; occurrenceID: YAU5082020Tt96; **Taxon:** scientificNameAuthorship: *Taeniothripsmusae* (Zhang & Tong); **Location:** country: China; stateProvince: Yunnan; municipality: Xishuangbanna; locality: Jinghong (Nabanhe Protected Area + Mansha Village); decimalLatitude: 21.916663; decimalLongitude: 101.193451; **Identification:** identifiedBy: Li Yajin; dateIdentified: 1987; identificationReferences: (ThripsWiki 2020); **Event:** samplingProtocol: sweeping and shaking; eventDate: 1987; **Record Level:** collectionID: thrips; institutionCode: YAU5082020; collectionCode: terebrantia; basisOfRecord: preserved specimen

##### Ecological interactions

###### Feeds on

leaves, bulbs, flowers and collected from Orchidaceae.

##### Distribution

Described from China (Xishuangbanna).

#### Tameothrips arundo

Tyagi, Kumar & Chauhan, 2015

6224D831-E70C-5C92-9BEE-C3672643ED8D

https://thrips.info/wiki/Tameothrips_arundo

##### Materials

- **Type status:** Other material. **Occurrence:** recordedBy: L.Y.J; individualID: 2017-III-10; individualCount: 11; sex: females; lifeStage: adults; occurrenceID: YAU5082020Tt97; **Taxon:** scientificNameAuthorship: *Tameothripsarundo* Tyagi, Kumar & Chauhan; **Location:** country: China; stateProvince: Yunnan; municipality: Xishuangbanna; locality: Different sites; decimalLatitude: 21.923101; decimalLongitude: 101.200925; **Identification:** identifiedBy: Li Yajin; dateIdentified: 2018; identificationReferences: (ThripsWiki 2020); **Event:** samplingProtocol: sweeping and shaking; eventDate: 10/03/2017; **Record Level:** collectionID: thrips; institutionCode: YAU5082020; collectionCode: terebrantia; basisOfRecord: preserved specimen

##### Ecological interactions

###### Feeds on

flowers and collected from arundo grasses.

##### Distribution

Described from India and recorded from south China.

#### Thrips andrewsi

(Bagnall, 1921)

5B4D7BF0-77AE-5ECC-9359-CB1DBADDD322

https://thrips.info/wiki/Thrips_andrewsi

Physothrips andrewsi Bagnall, 1921: 394
Taeniothrips ghoshi Bhatti, 1962: 35.

##### Materials

- **Type status:** Other material. **Occurrence:** recordedBy: L.Y.J; individualID: 2016-V-6; individualCount: 19; sex: 4 males, 15 females; lifeStage: adults; occurrenceID: YAU5082020Tt98; **Taxon:** scientificNameAuthorship: *Thripsandrewsi* (Bagnall); **Location:** country: China; stateProvince: Yunnan; municipality: Xishuangbanna; locality: Mengla (Tropical Botanical Garden); decimalLatitude: 21.919882; decimalLongitude: 101.191583; **Identification:** identifiedBy: Li Yajin; dateIdentified: 2018; identificationReferences: (ThripsWiki 2020); **Event:** samplingProtocol: sweeping and shaking; eventDate: 06/05/2016; **Record Level:** collectionID: thrips; institutionCode: YAU5082020; collectionCode: terebrantia; basisOfRecord: preserved specimen

##### Ecological interactions

###### Feeds on

flowers, collected from mango, longan and tea flowers.

##### Distribution

Described from India and worldwide distributed.

#### Thrips atactus

Bhatti, 1967

5E591735-15A6-59FD-99F8-16C788B5D695

https://thrips.info/wiki/Thrips_atactus

##### Materials

- **Type status:** Other material. **Occurrence:** recordedBy: L.Y.J & X.Y.L.; individualID: 2017-III-10|2017-III-11; individualCount: 7; sex: 2 males, 5 females; lifeStage: adults; occurrenceID: YAU5082020Tt99; **Taxon:** scientificNameAuthorship: *Thripsatactus* Bhatti; **Location:** country: China; stateProvince: Yunnan; municipality: Xishuangbanna; locality: Jinghong (Nabanhe Protected Area); decimalLatitude: 21.935842; decimalLongitude: 101.248356; **Identification:** identifiedBy: Li Yajin; dateIdentified: 2018; identificationReferences: (ThripsWiki 2020); **Event:** samplingProtocol: sweeping and shaking; eventDate: 10/03/2017, 11/03/2017; **Record Level:** collectionID: thrips; institutionCode: YAU5082020; collectionCode: terebrantia; basisOfRecord: preserved specimen

##### Ecological interactions

###### Feeds on

flowers, collected from Caricaceae, Rosaceae and Fagaceae.

##### Distribution

Described from India and widely distributed.

#### Thrips australis

(Bagnall, 1915)

B388C2A0-EE92-568A-A557-1242C0D2D800

https://thrips.info/wiki/Thrips_australis

Isoneurothrips australis Bagnall, 1915: 592
Thrips lacteicorpus Girault, 1926: 17
Thrips mediolineus Girault, 1926: 18
Anomalothrips amygdali Morgan, 1929: 5
Isoneurothrips marisabelae Ortiz, 1973: 119.

##### Materials

- **Type status:** Other material. **Occurrence:** recordedBy: L.Y.J; individualID: 2017-X-22; individualCount: 1; sex: female; lifeStage: adults; occurrenceID: YAU5082020Tt100; **Taxon:** scientificNameAuthorship: *Thripsaustralis* (Bagnall); **Location:** country: China; stateProvince: Yunnan; municipality: Xishuangbanna; locality: Jinghong (Botanical Garden); decimalLatitude: 21.926454; decimalLongitude: 101.254249; **Identification:** identifiedBy: Li Yajin; dateIdentified: 2018; identificationReferences: (ThripsWiki 2020); **Event:** samplingProtocol: sweeping and shaking; eventDate: 22/10/2017; **Record Level:** collectionID: thrips; institutionCode: YAU5082020; collectionCode: terebrantia; basisOfRecord: preserved specimen

##### Ecological interactions

###### Feeds on

flowers, collected from potato and eucalyptus.

##### Distribution

Described from Australia and widely distributed from where Eucalyptus hosts are distributed.

#### Thrips coloratus

(Schmutz, 1913)

2DEF598B-CABB-55CD-895E-94D1F86FAA57

https://thrips.info/wiki/Thrips_coloratus

Thrips colorata Schmutz, 1913: 1013
Thrips japonicus Bagnall, 1914: 288
Thrips melanurus Bagnall, 1926: 111
Thrips aligherini Girault, 1927: 1.

##### Materials

- **Type status:** Other material. **Occurrence:** recordedBy: S.S.Q; individualID: 2009-IV-17; individualCount: 56; sex: 17 males, 39 females; lifeStage: adults; occurrenceID: YAU5082020Tt101; **Taxon:** scientificNameAuthorship: *Thripscoloratus* (Schmutz); **Location:** country: China; stateProvince: Yunnan; municipality: Xishuangbanna; locality: Jinghong (Yexianggu); decimalLatitude: 21.930611; decimalLongitude: 101.258129; **Identification:** identifiedBy: Li Yajin; dateIdentified: 2018; identificationReferences: (ThripsWiki 2020); **Event:** samplingProtocol: sweeping and shaking; eventDate: 17/04/2009; **Record Level:** collectionID: thrips; institutionCode: YAU5082020; collectionCode: terebrantia; basisOfRecord: preserved specimen

##### Ecological interactions

###### Feeds on

flowers, collected from potatoes, mango and citrus.

##### Distribution

Described from Sri Lanka. Recorded from Japan, India, Australia and China.

#### Thrips flavus

Schrank, 1776

AD08A693-536D-522D-82C0-0240741F678B

https://thrips.info/wiki/Thrips_flavus

Thrips flavus Schrank, 1776: 31
Thrips melanopa Schrank, 1776: 31
Thrips ochraceus Curtis, 1841: 228
Physothrips flavidus Bagnall, 1916: 399
Thrips flavidus Bagnall, 1916: 402
Thrips flavosetosus Priesner, 1919: 105
Thrips obscuricornis Priesner, 1927: 423
Physothrips flavus Bagnall, 1928: 98
Thrips nilgiriensis Ramakrishna, 1928: 262
Taeniothrips clarus Moulton, 1928a: 287. Synonymised by Palmer (1992)
Thrips kyotoi Moulton, 1928b: 302 | Taeniothrips luteus Oettingen, 1935: 183
Taeniothrips sulfuratus Priesner, 1935: 358
Thrips biarticulata Priesner, 1935: 358
Taeniothrips saussureae Ishida, 1936: 70
Taeniothrips rhopalantennalis Shumsher, 1946: 181.

##### Materials

- **Type status:** Other material. **Occurrence:** recordedBy: L.Y.J; individualID: 2016-V-5; individualCount: 15; sex: 4 males, 11 females; lifeStage: adults; occurrenceID: YAU5082020Tt103; **Taxon:** scientificNameAuthorship: *Thripsflavus* Schrank; **Location:** country: China; stateProvince: Yunnan; municipality: Xishuangbanna; locality: Mengla; decimalLatitude: 21.937451; decimalLongitude: 101.257842; **Identification:** identifiedBy: Li Yajin; dateIdentified: 2018; identificationReferences: (ThripsWiki 2020); **Event:** samplingProtocol: sweeping and shaking; eventDate: 05/05/2016; **Record Level:** collectionID: thrips; institutionCode: YAU5082020; collectionCode: terebrantia; basisOfRecord: preserved specimen

##### Ecological interactions

###### Feeds on

flowers, leaves, collected from chinese rose, mango flowers and Fabaceae.

##### Distribution

Described from Austria and widely distributed.

#### Thrips hawaiiensis

(Morgan, 1913)

7AF08139-EDCA-583F-B919-91DCC654881E

https://thrips.info/wiki/Thrips_hawaiiensis

Euthrips hawaiiensis Morgan, 1913
Thrips sulphurea Schmutz, 1913: 1011
Thrips nigriflava Schmutz, 1913: 1012
Thrips albipes Bagnall, 1914: 25
Physothrips pallipes Bagnall, 1916: 400
Physothrips albipes Bagnall, 1916: 401
Bregmatothrips theifloris Karny, 1921: 66. Synonymised by Bhatti (1978)
Thrips versicolor Bagnall, 1926: 108
Thrips pallipes Bagnall, 1926: 110
Thrips io Girault, 1927: 351. Synonymised by Mound and Houston (1987)
Thrips partirufus Girault, 1927: 1. Synonymised by Mound & Houston, 1987: 9
Physothrips emersoni Girault, 1927: 2
Taeniothrips eriobotryae Moulton, 1928: 297. Synonymised by Bhatti (1970)
Physothrips lacteicolor Girault, 1928: 1
Physothrips marii Girault, 1928: 2 | Physothripsmjobergivar.darci Girault, 1930: 1. Synonymised by Mound and Houston (1987)
Thrips hawaiiensis form imitator Priesner, 1934: 267. Replacement name for *Physothripsalbipes* Bagnall
Taeniothrips pallipes var. florinatus Priesner, 1938: 489. Synonymised by Bhatti (1970)
Taeniothrips rhodomyrti Priesner, 1938: 492.

##### Materials

- **Type status:** Other material. **Occurrence:** recordedBy: E.N, L.Y.J, L.H & Z.H.R; individualID: 2017-III-11|2018-V-28|2014-V-28|2019-VIII-23; individualCount: 288; sex: 71 males, 217 females; lifeStage: adults; occurrenceID: YAU5082020Tt104; **Taxon:** scientificNameAuthorship: *Thripshawaiiensis* (Morgan); **Location:** country: China; stateProvince: Yunnan; municipality: Xishuangbanna; locality: Different sites; decimalLatitude: 21.932623; decimalLongitude: 101.271352; **Identification:** identifiedBy: Li Yajin; dateIdentified: 2019, 2018; identificationReferences: (ThripsWiki 2020); **Event:** samplingProtocol: sweeping and shaking; eventDate: 28/05/2014, 11/03/2017 28/05/2018, 23/08/2019; **Record Level:** collectionID: thrips; institutionCode: YAU5082020; collectionCode: terebrantia; basisOfRecord: preserved specimen

##### Ecological interactions

###### Feeds on

flowers, collected from Cannaceae, Irideae, Fabaceae, Cucurbitaceae, tea and mango.

##### Distribution

Described from Hawaii, USA and widely distributed.

#### Thrips orientalis

(Bagnall, 1915)

3D46A6A5-612B-5204-B06C-5DCCB9A4CE03

https://thrips.info/wiki/Thrips_orientalis

Isoneurothrips orientalis Bagnall, 1915: 593
Thrips setipennis Steinweden & Moulton, 1930: 25. Synonymised by Sakimura (1967)
Thrips hispidipennis Hood, 1932: 122.

##### Materials

- **Type status:** Other material. **Occurrence:** recordedBy: L.Y.J; individualID: 2017-X-25 & 2018-V-28; individualCount: 9; sex: 1 male, 8 females; lifeStage: adults; occurrenceID: YAU5082020Tt105; **Taxon:** scientificNameAuthorship: *Thripsorientalis* (Bagnall); **Location:** country: China; stateProvince: Yunnan; municipality: Xishuangbanna; locality: Mengla (Tropical Botanical Garden); decimalLatitude: 21.923369; decimalLongitude: 101.276527; **Identification:** identifiedBy: Li Yajin; dateIdentified: 2018; identificationReferences: (ThripsWiki 2020); **Event:** samplingProtocol: sweeping and shaking; eventDate: 25/10/2017, 28/05/2018; **Record Level:** collectionID: thrips; institutionCode: YAU5082020; collectionCode: terebrantia; basisOfRecord: preserved specimen

##### Ecological interactions

###### Feeds on

flowers and collected from jasmine.

##### Distribution

Described from Malaysia. Recorded from Tanzania, China and Japan.

#### Thrips palmi

Karny, 1925

85AFAE2D-2805-59BF-AA89-75E018F0CB61

https://thrips.info/wiki/Thrips_palmi

Thrips clarus Moulton, 1928: 294 | Thrips leucadophilus Priesner, 1936: 91
Thrips gossypicola Ramakrishna & Marghabandu, 1939: 41 | Chloethrips (Mictothrips) aureus Ananthakrishnan & Jagadish, 1967: 381. Synonymised by Bhatti (1970)
Thrips gracilis Ananthakrishnan & Jagadish, 1968: 361. Synonymised by Bhatti (1970).

##### Materials

- **Type status:** Other material. **Occurrence:** recordedBy: E.N, L.Y.J, L.H, Y,X.Q & Z.H.R; individualID: 2015-X-6|2015-X-7|2016-III-17|2016-V-11|2016-V-12|2017-IX-8|2017-IX-12|2017-VIII-9|2019-VIII-18; individualCount: 364; sex: 112 males, 252 females; lifeStage: adults; occurrenceID: YAU5082020Tt106; **Taxon:** scientificNameAuthorship: *Thripspalmi* Karny; vernacularName: melon thrips; **Location:** country: China; stateProvince: Yunnan; municipality: Xishuangbanna; locality: Mengla (Tropical Botanical Garden); decimalLatitude: 21.915456; decimalLongitude: 101.275664; **Identification:** identifiedBy: Li Yajin; dateIdentified: 2019, 2018; identificationReferences: (ThripsWiki 2020); **Event:** samplingProtocol: sweeping and shaking; eventDate: 06/10/2015, 07/10/2015, 11/05/2016, 12/05/2016, 17/03/2016, 12/05/2017, 09/08/2017, 08/09/2017, 18/08/2019; **Record Level:** collectionID: thrips; institutionCode: YAU5082020; collectionCode: terebrantia; basisOfRecord: preserved specimen

##### Ecological interactions

###### Feeds on

flowers, leaves. Collected from Apiaceae, pepper, eggplants, carrots and potatoes

##### Distribution

Described from Indonesia and widely distributed from the Tropics to Caribbean Regions.

#### Thrips subnudula

(Karny, 1926)

EF4388A5-84AC-526E-AB49-A61EBA197375

https://thrips.info/wiki/Thrips_subnudula

Ramaswamiahiella subnudula Karny, 1926: 208
Thrips pandu Ramakrishna, 1928: 264
Thrips setosus Moulton, 1929: 97 | Thrips temporatus Bailey, 1951: 19.

##### Materials

- **Type status:** Other material. **Occurrence:** recordedBy: S.S.Q; individualID: 2008-IV-23; individualCount: 15; sex: 2 males, 13 females; lifeStage: adults; occurrenceID: YAU5082020Tt107; **Taxon:** scientificNameAuthorship: *Thripssubnudula* (Karny); **Location:** country: China; stateProvince: Yunnan; municipality: Xishuangbanna; locality: Different sites; decimalLatitude: 21.911298; decimalLongitude: 101.285581; **Identification:** identifiedBy: Li Yajin; dateIdentified: 2018; identificationReferences: (ThripsWiki 2020); **Event:** samplingProtocol: sweeping and shaking; eventDate: 23/04/2008; **Record Level:** collectionID: thrips; institutionCode: YAU5082020; collectionCode: terebrantia; basisOfRecord: preserved specimen

##### Ecological interactions

###### Feeds on

flowers, collected from mango and amaranthus

##### Distribution

Described from India. Recorded from Pakistan, Bali, the Philippines, Uganda, Nigeria, Malaysia and China.

#### Thrips (Isoneurothrips) taiwanus

(Takahashi, 1936)

59369366-5EB8-5A46-AFAC-458D5DB731CC

https://www.gbif.org/species/4799922

Isoneurothrips parvispinus Karny, 1922: 106
Isoneurothrips jenseni Karny, 1925: 7. Synonymised by Priesner (1934)
Isoneurothrips pallipes Moulton, 1928: 296 | Thrips (Isoneurothrips) taiwanus Takahashi, 1936: 440.

##### Materials

- **Type status:** Other material. **Occurrence:** recordedBy: K.B; individualID: 2017-III-9; individualCount: 6; sex: 2 males, 4 females; lifeStage: adults; occurrenceID: YAU5082020Tt108; **Taxon:** scientificNameAuthorship: *Thripstaiwanus* (Takahashi); **Location:** country: China; stateProvince: Yunnan; municipality: Xishuangbanna; locality: Jinghong; decimalLatitude: 21.914786; decimalLongitude: 101.291474; **Identification:** identifiedBy: Li Yajin; dateIdentified: 2018; identificationReferences: (ThripsWiki 2020); **Event:** samplingProtocol: sweeping and shaking; eventDate: 09/03/2017; **Record Level:** collectionID: thrips; institutionCode: YAU5082020; collectionCode: terebrantia; basisOfRecord: preserved specimen

##### Ecological interactions

###### Feeds on

flowers and collected from Caricaceae.

##### Distribution

Described from Taiwan. Recorded from Thailand, Philippines, northern Australia and China.

#### Thrips tabaci

Lindeman, 1889

CD561FD3-8AED-56DE-A3F4-ADB6D53CEC3A

https://thrips.info/wiki/Thrips_tabaci

Thrips solanaceorum Widgalm in Portschinsky, 1883: 44
Limothrips allii Gillette, 1893: 15
Thrips communis Uzel, 1895: 176
Thrips communis annulicornis Uzel, 1895: 177
Thrips communis pulla Uzel, 1895: 177
Thrips flava obsoleta Uzel, 1895: 187
Thrips bremnerii Moulton, 1907: 59
Parathrips uzeli Karny, 1907: 48
Thrips bicolor Karny, 1907: 49
Thrips brachycephalus Enderlein, 1909: 441
Thrips hololeucus Bagnall, 1914: 24. Synonymised by Mound (1968)
Thrips adamsoni Bagnall, 1923: 58. Synonymy in Mound, 1968: 67
Thrips debilis Bagnall, 1923: 60
Thrips mariae Cotte, 1924: 2
Thrips frankeniae Bagnall, 1926: 654
Thrips seminiveus Girault, 1926: 1. Synonymised by Mound and Houston (1987)
Thrips tabaci f. irrorata Priesner, 1927: 436
Thrips tabaci f. nigricornis Priesner, 1927: 436
Thrips tabaci f. atricornis Priesner, 1927: 437
Thrips dorsalis Bagnall, 1927: 576. Synonymy in Mound (1968)
Thrips indigenus Girault, 1929: 29
Thrips dianthi Moulton, 1936: 104
Ramaswamiahiella kallarensis Ananthakrishnan, 1960: 564.

##### Materials

- **Type status:** Other material. **Occurrence:** recordedBy: E.N, L.Y.J & L.H; individualID: 2010-IV-19|2015-IV-23|2016-VII-27|2016-VIII-22|2019-X-27|2017-III-9; individualCount: 147; sex: 30 males, 117 females; lifeStage: adults; occurrenceID: YAU5082020Tt109; **Taxon:** scientificNameAuthorship: *Thripstabaci* (Widgalm in Portschinsk); vernacularName: Onion thrips; **Location:** country: China; stateProvince: Yunnan; municipality: Xishuangbanna; locality: Mengla; decimalLatitude: 21.92404; decimalLongitude: 101.28515; **Identification:** identifiedBy: Li Yajin; dateIdentified: 2019, 2018; identificationReferences: (ThripsWiki 2020); **Event:** samplingProtocol: sweeping and shaking; eventDate: 19/04/2010, 23/04/2015, 27/07/2016, 22/08/2016, 09/03/2017, 27/10/2019; **Record Level:** collectionID: thrips; institutionCode: YAU5082020; collectionCode: terebrantia; basisOfRecord: preserved specimen

##### Ecological interactions

###### Feeds on

flowers. Collected from onion, garlic, tobacco and mango.

##### Distribution

Described from Moldova and worldwide distributed.

#### Trachynotothrips striatus

Masumoto & Okajima, 2005

4BB094DE-C14B-597F-BAEB-50A708E132D8

https://thrips.info/wiki/Trachynotothrips_striatus

##### Materials

- **Type status:** Other material. **Occurrence:** recordedBy: L.Y.J; individualID: 2017-X-24; individualCount: 11; sex: female; lifeStage: adults; occurrenceID: YAU5082020Tt110; **Taxon:** scientificNameAuthorship: *Trachynotothripsstriatus* Masumoto & Okajima; **Location:** country: China; stateProvince: Yunnan; municipality: Xishuangbanna; locality: Jinghong; decimalLatitude: 21.926856; decimalLongitude: 101.317346; **Identification:** identifiedBy: Li Yajin; dateIdentified: 2018; identificationReferences: (ThripsWiki 2020); **Event:** samplingProtocol: sweeping and shaking; eventDate: 24/10/2017; **Record Level:** collectionID: thrips; institutionCode: YAU5082020; collectionCode: terebrantia; basisOfRecord: preserved specimen

##### Ecological interactions

###### Feeds on

leaves and collected from Poaceae.

##### Distribution

Described from Vietnam. Recorded from India and China.

##### Diagnosis

**Figure 19.**
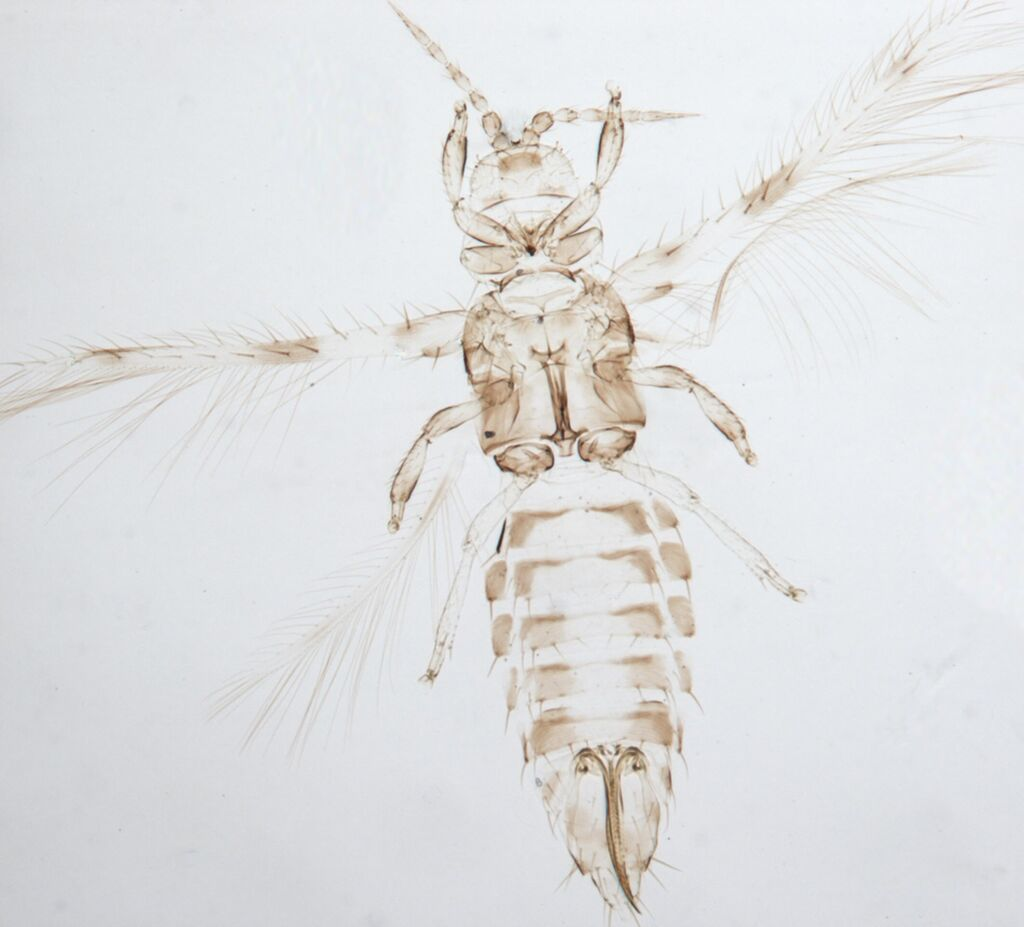
Female of *Trachynotothripsstriatus* Matsumoto and Okajima, 2005.

The female fully-winged; body mainly paler with brown markings (Fig. 19), head wider than long, constricted just behind compound eyes paler with shaded cheeks; antennal segments I and III yellowish-white, II and VI to VIII brown, IV yellowish-white with distal half brown, segment V yellowish-white with distal third brown; pronotum yellowish-white with two submarginal longitudinal brown bands; fore wings with alternate four white areas and three brown bands; abdominal terga II to VII sculptured. Male generally similar to female, but slightly smaller, sternites III-VIII with scattered small pore plates.

#### Trichromothrips alis

Bhatti, 1967

A9AD16DB-734B-5F32-8EBD-EFBD94F33A7D

https://thrips.info/wiki/Trichromothrips_alis

##### Materials

- **Type status:** Other material. **Occurrence:** recordedBy: L.Y.J; individualID: 2017-X-24; individualCount: 10; sex: males; lifeStage: adults; occurrenceID: YAU5082020Tt111; **Taxon:** scientificNameAuthorship: *Trichromothripsalis* Bhatti; **Location:** country: China; stateProvince: Yunnan; municipality: Xishuangbanna; locality: Mengla (Tropical Botanical Garden); decimalLatitude: 21.927891; decimalLongitude: 101.311502; **Identification:** identifiedBy: Li Yajin; dateIdentified: 2018; identificationReferences: (ThripsWiki 2020); **Event:** samplingProtocol: sweeping and shaking; eventDate: 24/10/2017; **Record Level:** collectionID: thrips; institutionCode: YAU5082020; collectionCode: terebrantia; basisOfRecord: preserved specimen

##### Ecological interactions

###### Feeds on

leaves and collected from cosmianthemum.

##### Distribution

Described from India and recorded from China.

#### Trichromothrips antidesmae

Li, Li & Zhang, 2019

0A54E5F2-791B-5F64-98F7-338EAC834633

https://thrips.info/wiki/Trichromothrips_antidesmae

##### Materials

- **Type status:** Other material. **Occurrence:** recordedBy: L.H & K.B; individualID: 2017-X-24|2017-III-12|2017-III-10; individualCount: 7; sex: 2 males, 5 females; lifeStage: adults; occurrenceID: YAU5082020Tt112; **Taxon:** scientificNameAuthorship: *Trichromothripsantidesmae* Li, Li & Zhang; **Location:** country: China; stateProvince: Yunnan; municipality: Xishuangbanna; locality: Mengla (Tropical Botanical Garden); decimalLatitude: 21.959907; decimalLongitude: 100.463502; **Identification:** identifiedBy: Li Yajin; dateIdentified: 2018; identificationReferences: (ThripsWiki 2020); **Event:** samplingProtocol: sweeping and shaking; eventDate: 10/03/2017, 12/03/2017, 24/10/2017; **Record Level:** collectionID: thrips; institutionCode: YAU5082020; collectionCode: terebrantia; basisOfRecord: preserved specimen

##### Ecological interactions

###### Feeds on

leaves and collected from antidesma.

##### Distribution

Described from China (Xishuangbanna) (Li et al. 2019).

##### Diagnosis

**Figure 20.**
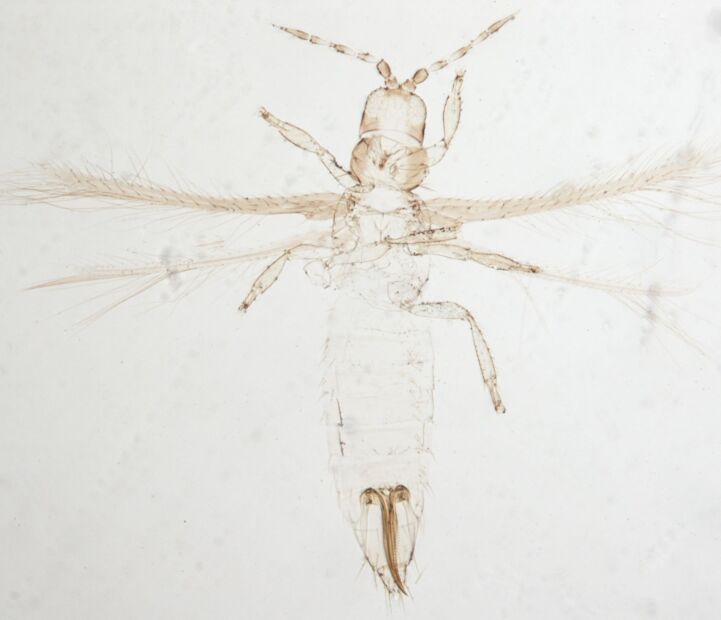
Female of *Trichromothripsantidesmae* Li, Li & Zhang, 2019.

Female macropterous; body and legs yellow (Fig. 20), head and pronotum with dark lateral margin, antennal segments I–II brown, III–V yellow with the apex brown, VI-VII brown, VIII pale brown; fore wing brown with apex pale, clavus brown; abdominal tergites with transverse striae laterally, tergites II-VII with three setae arranged in a straight line. Male generally similar in structure and colour similar to female, sternites without pore plates.

#### Trichromothrips assamensis

Tyagi & Kumar, 2017

46072FDB-CB60-5C6F-ADDC-BF5B5B07648D

https://thrips.info/wiki/Trichromothrips_assamensis

##### Materials

- **Type status:** Other material. **Occurrence:** recordedBy: X.Y.L; individualID: 2017-X-24|2018-VI-1; individualCount: 6; sex: females; lifeStage: adults; occurrenceID: YAU5082020Tt113; **Taxon:** scientificNameAuthorship: *Trichromothripsassamensis* Tyagi & Kumar; **Location:** country: China; stateProvince: Yunnan; municipality: Xishuangbanna; locality: Mengla (Tropical Botanical Garden); decimalLatitude: 21.959857; decimalLongitude: 100.46016; **Identification:** identifiedBy: Li Yajin; dateIdentified: 2018; identificationReferences: (ThripsWiki 2020); **Event:** samplingProtocol: sweeping and shaking; eventDate: 24/10/2017, 01/06/2018; **Record Level:** collectionID: thrips; institutionCode: YAU5082020; collectionCode: terebrantia; basisOfRecord: preserved specimen

##### Ecological interactions

###### Feeds on

leaves and collected from Poaceae.

##### Distribution

Described form India (Tyagi et al. 2017). Recorded from China (Xishuangbanna).

#### Trichromothrips crispator

(Karny, 1915)

7276203F-F2A2-5017-BF09-C9EE87996AB9

https://thrips.info/wiki/Trichromothrips_crispator

Physothrips crispator Karny, 1915: 35.

##### Materials

- **Type status:** Other material. **Occurrence:** recordedBy: X.Y.L; individualID: 2018-VI-1; individualCount: 6; sex: females; lifeStage: adults; occurrenceID: YAU5082020Tt114; **Taxon:** scientificNameAuthorship: *Trichromothripscrispator* (Karny); **Location:** country: China; stateProvince: Yunnan; municipality: Xishuangbanna; locality: Different sites; decimalLatitude: 21.963879; decimalLongitude: 100.457142; **Identification:** identifiedBy: Li Yajin; dateIdentified: 2018; identificationReferences: (ThripsWiki 2020); **Event:** samplingProtocol: sweeping and shaking; eventDate: 01/06/2018; **Record Level:** collectionID: thrips; institutionCode: YAU5082020; collectionCode: terebrantia; basisOfRecord: preserved specimen

##### Ecological interactions

###### Feeds on

leaves and collected from Acanthaceae.

##### Distribution

Described from Indonesia and recorded from China.

#### Trichromothrips falcus

(Bhatti, 1967)

02D2802C-E55E-516A-9FDB-CAD6BCF3F5DA

https://thrips.info/wiki/Trichromothrips_falcus

Dorcadothrips fasciatus Bhatti, 1967: 21
Trichromothrips falcus Bhatti, 1999: 3.

##### Materials

- **Type status:** Other material. **Occurrence:** recordedBy: S.S.Q; individualID: 2011-IX-30; individualCount: 4; sex: females; lifeStage: adults; occurrenceID: YAU5082020Tt115; **Taxon:** scientificNameAuthorship: *Trichromothripsfalcus* (Bhatti); **Location:** country: China; stateProvince: Yunnan; municipality: Xishuangbanna; locality: Mengla (Tropical Botanical Garden); decimalLatitude: 21.954225; decimalLongitude: 100.44859; **Identification:** identifiedBy: Li Yajin; dateIdentified: 2018; identificationReferences: (ThripsWiki 2020); **Event:** samplingProtocol: sweeping and shaking; eventDate: 30/09/2011; **Record Level:** collectionID: thrips; institutionCode: YAU5082020; collectionCode: terebrantia; basisOfRecord: preserved specimen

##### Ecological interactions

###### Feeds on

leaves and collected from Poaceae.

##### Distribution

Described from India and recorded from China.

#### Trichromothrips formosus

Masumoto & Okajima, 2005

4D18476E-722B-5FE9-A742-0AB58F7060C2

https://thrips.info/wiki/Trichromothrips_formosus

##### Materials

- **Type status:** Other material. **Occurrence:** recordedBy: Z.C.H; individualID: 2018-V-19; individualCount: 1; sex: male; lifeStage: adults; occurrenceID: YAU5082020Tt116; **Taxon:** scientificNameAuthorship: *Trichromothripsformosus* Masumoto & Okajima; **Location:** country: China; stateProvince: Yunnan; municipality: Xishuangbanna; locality: Mengla (Bubang Village); decimalLatitude: 21.962539; decimalLongitude: 100.446218; **Identification:** identifiedBy: Li Yajin; dateIdentified: 2018; identificationReferences: (ThripsWiki 2020); **Event:** samplingProtocol: sweeping and shaking; eventDate: 19/05/2018; **Record Level:** collectionID: thrips; institutionCode: YAU5082020; collectionCode: terebrantia; basisOfRecord: preserved specimen

##### Ecological interactions

###### Feeds on

leaves and collected from Chloranthaceae.

##### Distribution

Described from Japan. Recorded from south-eastern China and Taiwan.

#### Trichromothrips guizhouensis

Li, Li & Zhang, 2019

43105C9A-28C9-5239-AA9B-3E85F95F2A3F

https://thrips.info/wiki/Trichromothrips_guizhouensis

##### Materials

- **Type status:** Other material. **Occurrence:** recordedBy: L.Y.J; individualID: 2018-V-28|2018-V-30|2018-V-31; individualCount: 4; sex: 2 males, 2 females; lifeStage: adults; occurrenceID: YAU5082020Tt117; **Taxon:** scientificNameAuthorship: *Trichromothripsguizhouensis* Li, Li & Zhang; **Location:** country: China; stateProvince: Yunnan; municipality: Xishuangbanna; locality: Mengla (Tropical Botanical Garden); decimalLatitude: 21.963879; decimalLongitude: 100.359622; **Identification:** identifiedBy: Li Yajin; dateIdentified: 2018; identificationReferences: (ThripsWiki 2020); **Event:** samplingProtocol: sweeping and shaking; eventDate: 28/05/2018, 30/05/2018, 31/05/2018; **Record Level:** collectionID: thrips; institutionCode: YAU5082020; collectionCode: terebrantia; basisOfRecord: preserved specimen

##### Ecological interactions

###### Feeds on

leaves and collected from Sapotaceae.

##### Distribution

Distributed from Guizhou and distributed from southeast of China.

#### Trichromothrips indicus

(Bhatti, 1978)

BB1C3204-1885-506F-8F84-30A65E1A3528

https://thrips.info/wiki/Trichromothrips_indicus

Dorcadothrips indicus Bhatti, 1978: 423.

##### Materials

- **Type status:** Other material. **Occurrence:** recordedBy: S.S.Q; individualID: 2011-IX-30; individualCount: 3; sex: 2 males, 1 female; lifeStage: adults; occurrenceID: YAU5082020Tt118; **Taxon:** scientificNameAuthorship: *Trichromothripsindicus* (Bhatti); **Location:** country: China; stateProvince: Yunnan; municipality: Xishuangbanna; locality: Mengla; decimalLatitude: 22.045911; decimalLongitude: 100.479779; **Identification:** identifiedBy: Li Yajin; dateIdentified: 2018; identificationReferences: (ThripsWiki 2020); **Event:** samplingProtocol: sweeping and shaking; eventDate: 30/09/2011; **Record Level:** collectionID: thrips; institutionCode: YAU5082020; collectionCode: terebrantia; basisOfRecord: preserved specimen

##### Ecological interactions

###### Feeds on

leaves and corrected from Poaceae.

##### Distribution

Described from India and recorded from south China.

#### Trichromothrips trifasciatus

(Priesner, 1936)

3E859F9B-A9D7-5320-A477-4144531E78C8

https://thrips.info/wiki/Trichromothrips_trifasciatus

Taeniothrips trifasciatus Priesner, 1936: 323.

##### Materials

- **Type status:** Other material. **Occurrence:** recordedBy: L.Y.J; individualID: 2017-X-22; individualCount: 4; sex: females; lifeStage: adults; occurrenceID: YAU5082020Tt119; **Taxon:** scientificNameAuthorship: *Trichromothripstrifasciatus* (Priesner); **Location:** country: China; stateProvince: Yunnan; municipality: Xishuangbanna; locality: Mengla (Tropical Botanical Garden); decimalLatitude: 21.917631; decimalLongitude: 100.407825; **Identification:** identifiedBy: Li Yajin; dateIdentified: 2018; identificationReferences: (ThripsWiki 2020); **Event:** samplingProtocol: sweeping and shaking; eventDate: 22/10/2017; **Record Level:** collectionID: thrips; institutionCode: YAU5082020; collectionCode: terebrantia; basisOfRecord: preserved specimen

##### Ecological interactions

###### Feeds on

leaves and collected from potato leaves.

##### Distribution

Described from Indonesia and recorded from China (Xishuangbanna).

##### Diagnosis

**Figure 21.**
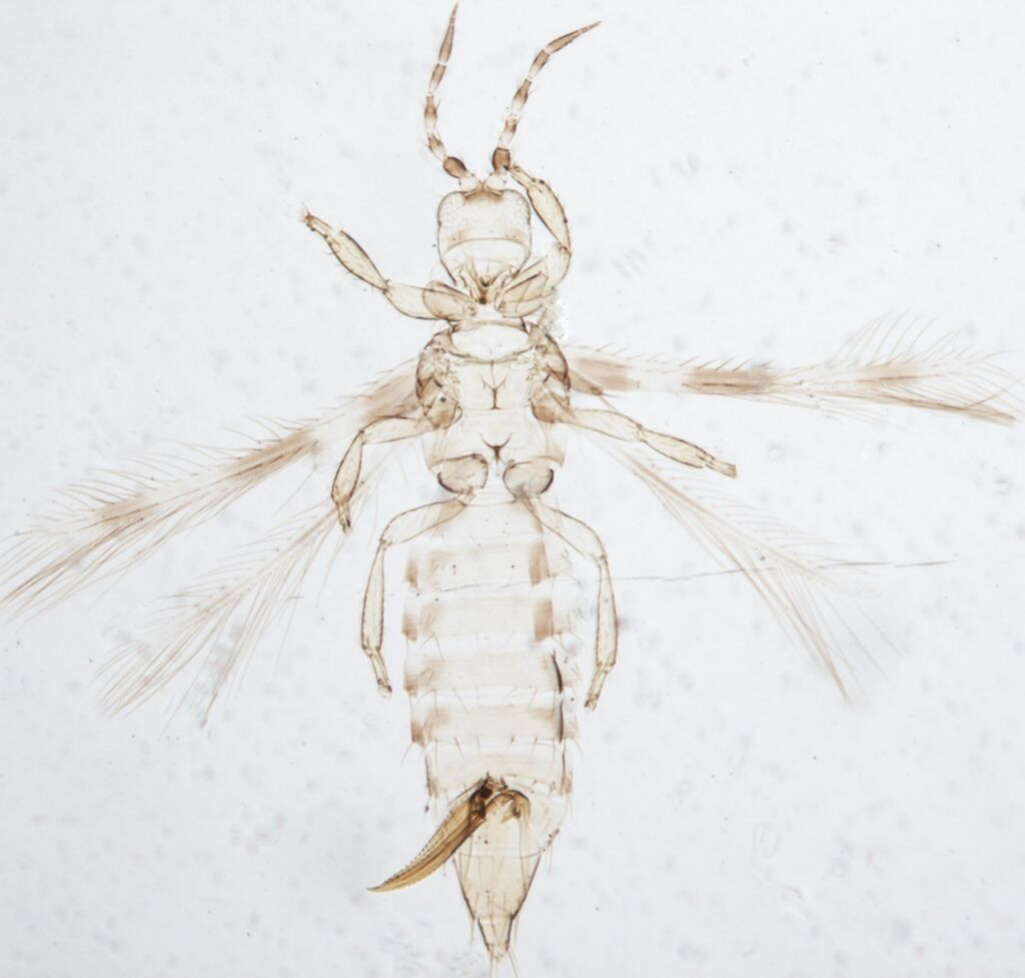
Female of *Trichromothripstrifasciatus* Priesner, 1936.

*Trichromothripstrifasciatus* differs from other species of the same genus by fore wing base not shaded along the anterior margin, basal half of antennal segments III–V yellow and abdominal tergites with some light brown patches (Fig. 21).

#### Tusothrips immaculatus

Reyes, 1994

82E36407-806C-5DE6-A74F-DB9FACE09DAE

https://thrips.info/wiki/Tusothrips_immaculatus

##### Materials

- **Type status:** Other material. **Occurrence:** recordedBy: L.Y.J & Y.Y.H; individualID: 2017-VIII-11|2017-X-24; individualCount: 9; sex: 6 males, 3 female; lifeStage: adults; occurrenceID: YAU5082020Tt120; **Taxon:** scientificNameAuthorship: *Tusothripsimmaculatus* Reyes; **Location:** country: China; stateProvince: Yunnan; municipality: Xishuangbanna; locality: Different sites; decimalLatitude: 21.961529; decimalLongitude: 100.462693; **Identification:** identifiedBy: Li Yajin; dateIdentified: 2018; identificationReferences: (ThripsWiki 2020); **Event:** samplingProtocol: sweeping and shaking; eventDate: 11/07/2017, 24/10/2017; **Record Level:** collectionID: thrips; institutionCode: YAU5082020; collectionCode: terebrantia; basisOfRecord: preserved specimen

##### Ecological interactions

###### Feeds on

leaves and collected from Rutaceae.

##### Distribution

Described from Philippines and recorded from China (Xishuangbanna).

#### Tusothrips sumatrensis

(Karny, 1925)

ECB4F4E5-13C2-5DED-BD20-62C8F659CD95

https://thrips.info/wiki/Tusothrips_sumatrensis

Anaphothrips sumatrensis Karny, 1925: 27 | Anaphothrips (Chaetanaphothrips) aureus Moulton, 1936: 266
Mycterothrips pseudosetiprivus Ramakrishna & Margabandhu, 1939: 42
Taeniothrips calopgomi Zhang, 1981: 324. Synonymised by Mirab-balou and Tong (2015).

##### Materials

- **Type status:** Other material. **Occurrence:** recordedBy: E.N, L.Y.J & X.Y.L; individualID: 2017-X-21| 2018-VI-1| 2018-VI-2; individualCount: 12; sex: 2 males, 10 females; lifeStage: adults; occurrenceID: YAU5082020Tt121; **Taxon:** scientificNameAuthorship: *Tusothripssumatrensis* (Karny); **Location:** country: China; stateProvince: Yunnan; municipality: Xishuangbanna; locality: Different sites; decimalLatitude: 21.96147; decimalLongitude: 100.462909; **Identification:** identifiedBy: Li Yajin; dateIdentified: 2018; identificationReferences: (ThripsWiki 2020); **Event:** samplingProtocol: sweeping and shaking; eventDate: 21/10/2017, 01/06/2018, 02/06/2018; **Record Level:** collectionID: thrips; institutionCode: YAU5082020; collectionCode: terebrantia; basisOfRecord: preserved specimen

##### Ecological interactions

###### Feeds on

leaves, collected from Urticaceae and Acanthaceae.

##### Distribution

Described from Japan and recorded from China.

#### Yoshinothrips pasekamui

Kudo, 1985

83DDFB65-5184-5CB2-A6B5-D5B8F529AD13

https://thrips.info/wiki/Yoshinothrips_pasekamui

##### Materials

- **Type status:** Other material. **Occurrence:** recordedBy: E.N & X.Y.L; individualID: 2018-VI-2| 2018-V-28; individualCount: 2; sex: females; lifeStage: adults; occurrenceID: YAU5082020Tt122; **Taxon:** scientificNameAuthorship: *Yoshinothripspasekamui* Kudo; **Location:** country: China; stateProvince: Yunnan; municipality: Xishuangbanna; locality: Jinghong; decimalLatitude: 21.959819; decimalLongitude: 100.460016; **Identification:** identifiedBy: Li Yajin; dateIdentified: 2018; identificationReferences: (ThripsWiki 2020); **Event:** samplingProtocol: sweeping and shaking; eventDate: 28/05/2018, 02/06/2018; **Record Level:** collectionID: thrips; institutionCode: YAU5082020; collectionCode: terebrantia; basisOfRecord: preserved specimen

##### Ecological interactions

###### Feeds on

leaves and collected from bamboo.

##### Distribution

Described from Japan and recorded from China (Xishuangbanna).

## Analysis

### Surveyed plant families

During this survey, 48% of the plants surveyed were herbaceous (deciduous or evergreen plants), 38% shrubby (annuals or biennials plants), 13% woody (perennial plants) and 1% for others (fungi, rocks and mosses). Fabaceae, Poaceae and Asteraceae showed a high population, whereas Bryophytes (mosses) and Pteridophyta (ferns) showed the low population dynamics of thrips species.

### Thysanoptera (suborder Terebrantia): species composition and distribution

**Figure 22.**
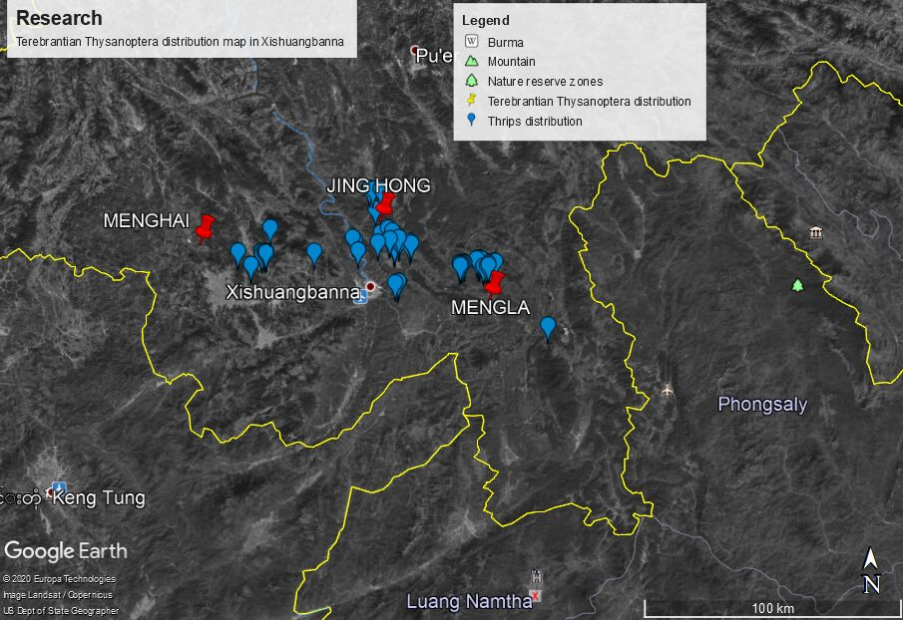
Google map showing distribution of Terebrantian (Thysanoptera) in the surveyed three counties, with which Jinghong is marked with the highest distributional points.

A total of 116 species in 55 genera within the families Aeolothripidae and Thripidae were recorded. Thripidae is represented with 115 species, whereas Aelothripidae is represented with a single species. The genus *Thrips* is represented with 11 species and *Trichromothrips* with 10 species, both being the most commonly encountered taxa. *Dichromomothripsnakahari* Mound, 1976 (subfamily Thripinae) and *Phibalothripsrugosus* Kudo, 1979 (subfamily Panchaetothripinae) are newly recorded in China. A distribution map (Fig. 22) of Terebrantian (Thysanoptera) in Xishuangbanna is provided with supplementary material for details (Suppl. material 1).

## Discussion

In this study, we found that Xishuangbanna shows the high thrips species composition with asymmetric geographical distribution as demonstrated in the world distribution of Thysanoptera (Mound and Marullo 1998) and (Mound 1983). Recorded species are representing 40.3% of the 313 species of the Terebrantian (Thysanoptera) in China. In addition, this place is the home of some rare genera and species, such as *Araliacothrips* Li, Li & Zhang, *Filicopsothripspulcher* Li, Yuan & Zhang, *Amomothrips* Bhatti and different predatory species of the family Aeolothripidae. *Dichromomothripsnakahari* Mound, 1976 (subfamily Thripinae), described from Indonesia (Mound 1976) and *Phibalothripsrugosus* Kudo, 1979 (subfamily Panchaetothripinae), described from Kuala Lumpur, Malaysia (ThripsWiki 2020), are newly-recorded species in Chinese fauna. Our results suggest that Xishuangbanna is the richest area in Thysanoptera species composition to be taken into consideration for biodiversity conservation. However, some species from the references were lacking sufficient information for the records and deposition to be the common challenge in the taxonomic study (Kuznetsova and Ivanova 2020) and suggest that detailed inventories on the specimens records and modernised open, readily accessible online or digitised data are required for sustainable taxonomic studies, as suggested by Specht et al. (2018). Besides, further investigations are suggested to explore the species diversity and variation within Thysanoptera ecosystem services provided in this area for sustainable biodiversity conservation.

## Supplementary Material

34153DD7-3951-5FA4-BC83-E70C45DA6290 10.3897/BDJ.9.e72670.suppl1 Supplementary material 1 Supplementary material Data type Occurences Brief description Supplementary data of 116 species representing 55 genera of thrips collected from Xishuangbanna, China. File: oo_611387.xls https://binary.pensoft.net/file/611387 Ntirenganya Elie, Li Yajin, Xie Yanlan, Zhou Yanli, Zhang Hongrui
